# Bioactive polyketides and meroterpenoids from the mangrove-derived fungus *Talaromyces flavus* TGGP35

**DOI:** 10.3389/fmicb.2024.1342843

**Published:** 2024-02-01

**Authors:** Jin Cai, Xueming Zhou, Bin Wang, Xuelong Zhang, Mengyao Luo, Longtao Huang, Ruoxi Wang, Yonghao Chen, Xiaoyang Li, Youping Luo, Guangying Chen, Fei Cao, Guolei Huang, Caijuan Zheng

**Affiliations:** ^1^Key Laboratory of Tropical Medicinal Resource Chemistry of Ministry of Education, College of Chemistry and Chemical Engineering, Hainan Normal University, Haikou, Hainan, China; ^2^Key Laboratory of Tropical Medicinal Plant Chemistry of Hainan Province, Haikou, Hainan, China; ^3^Key Laboratory of Pharmaceutical Quality Control of Hebei Province, Key Laboratory of Medicinal Chemistry and Molecular Diagnostics of Education Ministry of China, College of Pharmaceutical Sciences, Hebei University, Baoding, China

**Keywords:** *Talaromyces flavus*, polyketide, lactones, meroterpenoid, bioactivities

## Abstract

Six new polyketides, which includes three new lactones (talarotones A–C) **(1–3)**, one new polyketide (talarotide A) **(4)**, two new polyenes (talaroyenes A, B) **(5, 6)**, together with one new meroterpenoid (talaropenoid A) **(7)** and 13 known compounds **(8–20)** were isolated from the mangrove-derived fungus *Talaromyces flavus* TGGP35. The structure and configuration of the compounds **1**–**7** were elucidated from the data obtained from HR-ESI-MS, IR, 1D/2D NMR spectroscopy, Mo_2_ (OAc)_4_-induced electronic circular dichroism (ECD), CD spectroscopy, and modified Mosher's method. Compounds **5** and **20** displayed antioxidant activity with IC_50_ values of 0.40 and 1.36 mM, respectively. Compounds **3**, **6**, **11**, **16**, and **17** displayed cytotoxic activity against human cancer cells Hela, A549, and had IC_50_ values ranging from 28.89 to 62.23 μM. Compounds **7**, **10**–**12**, and **14**–**18** exhibited moderate or potent anti-insect activity against newly hatched larvae of *Helicoverpa armigera* Hubner, with IC_50_ values in the range 50–200 μg/mL. Compound **18** showed antibacterial activity against *Ralstonia solanacearum* with the MIC value of 50 μg/mL.

## Graphical Abstract

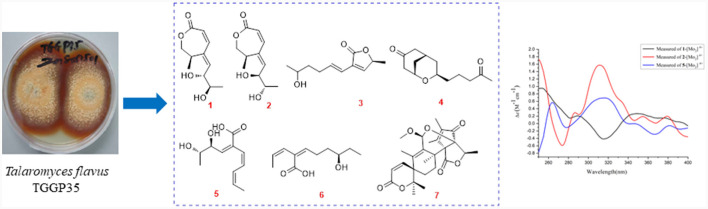

## 1 Introduction

The genus *Talaromyces* is identified as the sexual state of *Penicillium* and belongs to the Trichocomaceae family (Chaiyosang et al., 2021). The fungus *Talaromyces*, which is broadly dispersed in the natural environment (Zhang K. et al., 2022), demonstrates significant potential as a biological resource in food industry, ecology, agriculture, and medicine (Nicoletti et al., 2018; Devi et al., 2020; Prieto et al., 2021; Yadav et al., 2022; Aggarwal et al., 2023; De Eugenio et al., 2023; Xue et al., 2023). To further understand its characteristics, the biosynthetic pathways, biological activity (Chen et al., 2022; Lei et al., 2022; Lv et al., 2023; Nicoletti et al., 2023), and structure–activity relationship (Xie et al., 2022; Zhang M. et al., 2022) in *Talaromyces* have been extensively probed. These studies have also shown that *Talaromyces* exhibits numerous beneficial bioactivities and exceptional biosynthetic capabilities, which makes it suitable for wide-ranging industrial applications.

The genus *Talaromyces* has the capability to produce novel secondary metabolites with potent biological activities (Lei et al., 2022), such as antimicrobial depsidone (talaronin E) (Nicoletti et al., 2023) and polyketide (tanicutone B) (Wang et al., 2023), cytotoxic cytochalasan (talachalasin A) (Ding et al., 2023), dimeric oxaphenalenone aminoglycoside (glyclauxin D) (Samarasekera et al., 2023), antiviral cytochalasan (talachalasin B) (Ding et al., 2023), anti-inflammatory α-pyrone derivative (talarolactones E and F) (Li et al., 2023), phthalides (amestolkins A and B) (Huang et al., 2023), and insecticidal alkaloid (talaroenamine D) (Zang et al., 2015). Therefore, *Talaromyces* can be used to synthesize novel compounds (Kumari et al., 2018; Lan and Wu, 2020).

**Figure 1:**
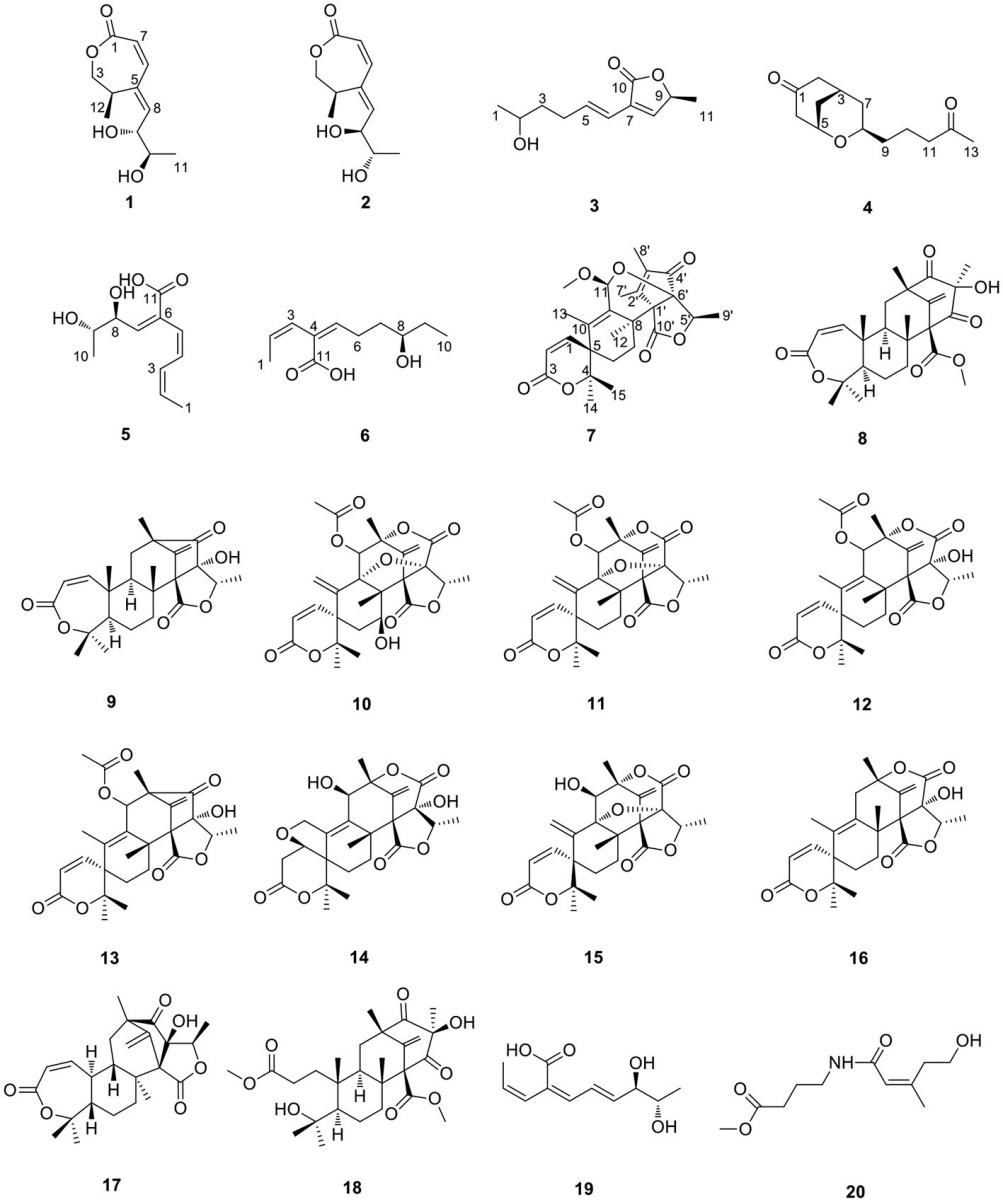
The structures of compounds **1–20**.

As a part of our ongoing investigation to identify secondary metabolites from marine-derived fungus with a novel structure and potent bioactivity (Bai et al., 2019a,b, 2021; Liao et al., 2019), we obtained six new isocoumarins from the fungus *Talaromyces flavus* TGGP35 (separated from the medicinal mangrove *Acanthus ilicifolius*) (Cai et al., 2022). With the aim of isolating more compounds with potent bioactivity from *T. flavus* TGGP35, the solid-state fermentation condition was changed (sea salt was replaced by sodium bromide). During our investigations, we also found that EtOAc extract, a modified fermentation extract from *T. flavus* TGGP35, showed potent anti-insect activity against newly hatched larvae of *Helicoverpa armigera* Hubner with IC_50_ value of 200 μg/mL. We carried out bioassay-guided separation following the isolation of the EtOAc extract and isolated three new lactones (talarotones A–C) (**1**–**3**), one new polyketide (talarotide A) (**4**), two new polyenes (talaroyenes A and B) (**5,6**), one new meroterpenoid (talaropenoid A) (**7**), along with 13 known compounds (**8**–**20**) (Figure 1), and characterized them. In this paper, we describe the isolation, determination of the structure, and the bioactivities of these compounds.

## 2 Results and discussion

**Figure 2:**
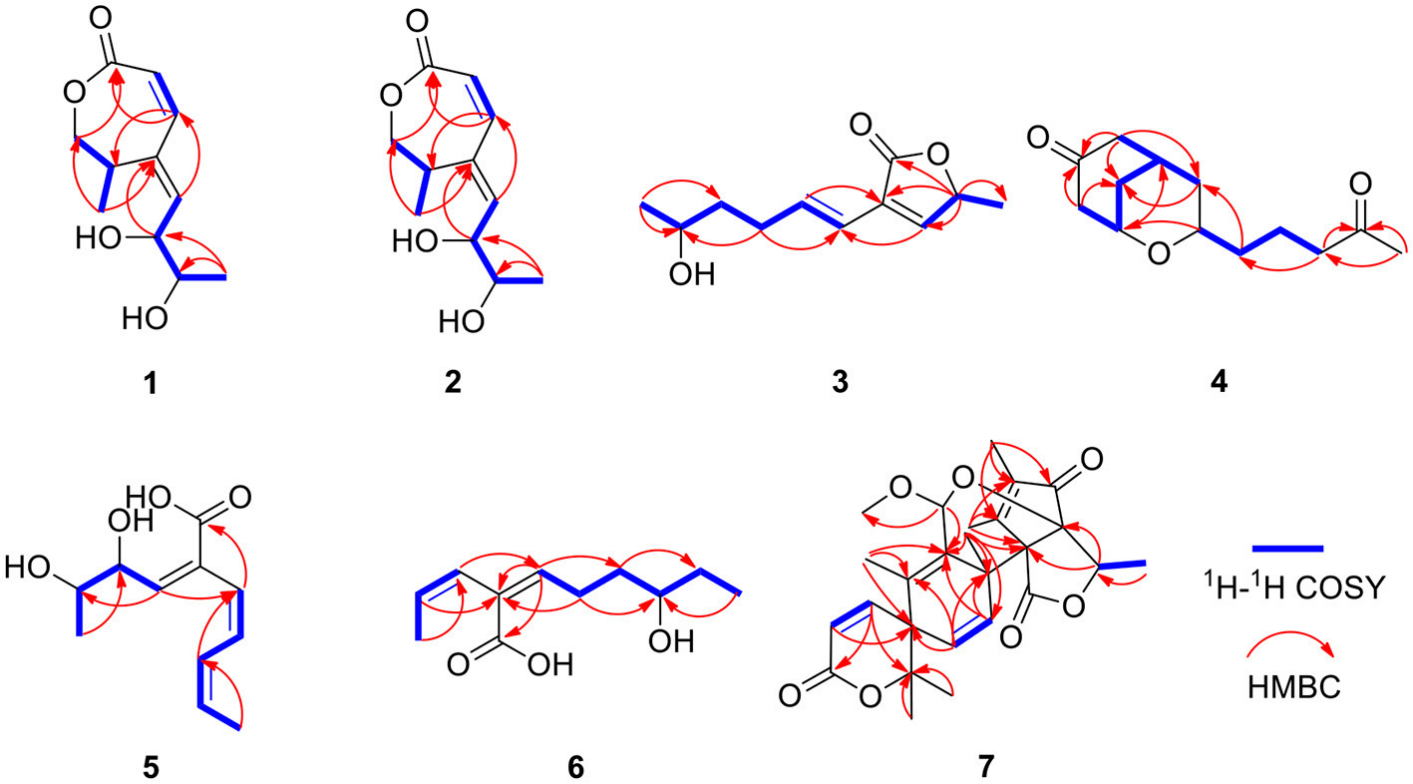
^1^H-^1^H COZY and key HMBC correlations for compounds **1**–**7**.

**Figure 3:**
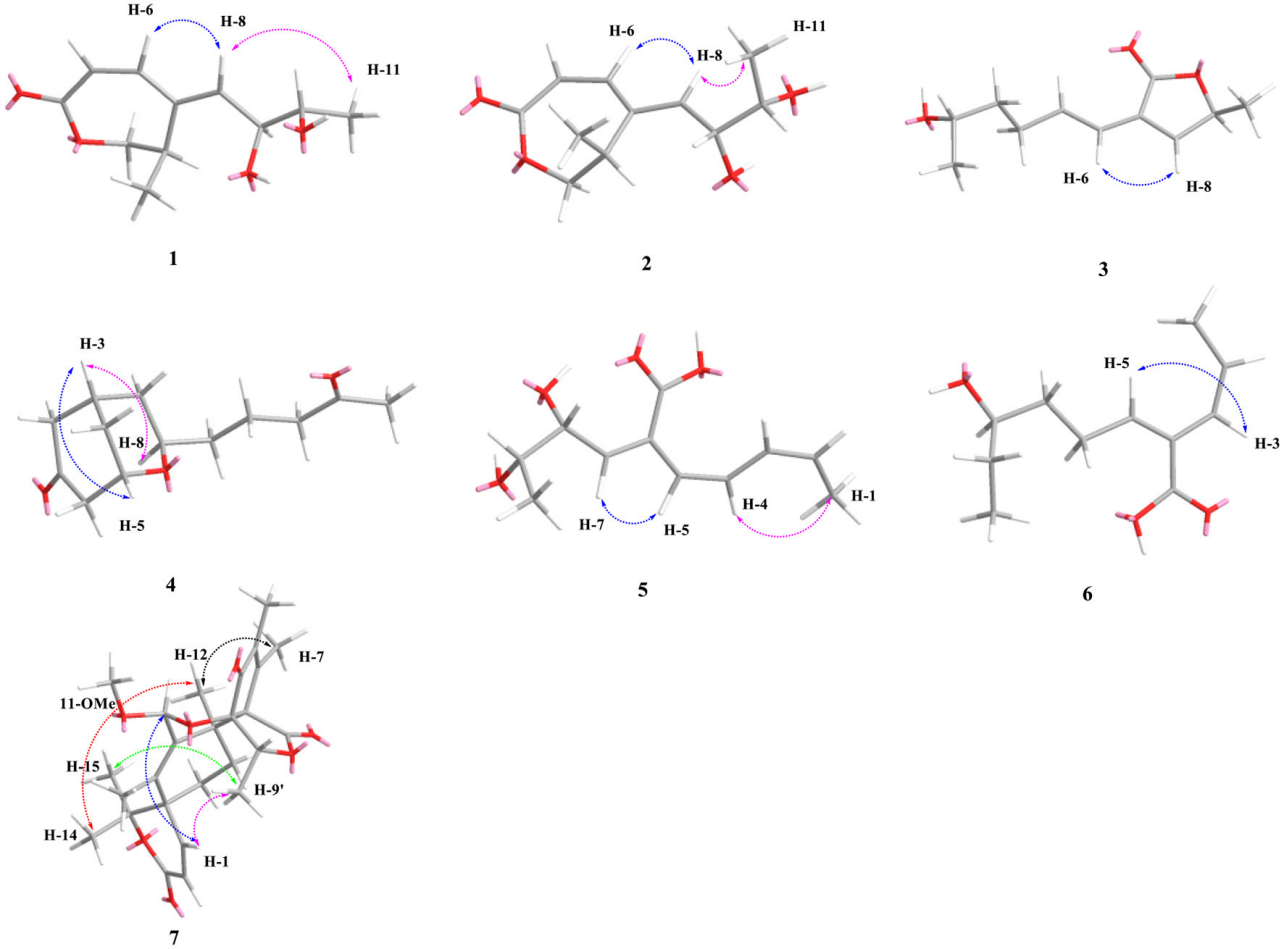
Key NOESY correlations of **1**–**7**.

Compound **1** was obtained as a yellow oil. From the HR-ESI-MS data [*m/z* 235.0939 [M + Na]^+^ (C_11_H_16_O_4_Na^+^, calcd. for 235.0941)], its molecular formula was determined as C_11_H_16_O_4_ (four degrees of unsaturation). The IR spectrum showed the presence of a hydroxyl group (3,534, 3,426 cm^−1^), an ester group (1,727 cm^−1^) and an olefine group (1,627, 1,618 cm^−1^) and compound **1**. The ^1^H-NMR data (Table 1) revealed three olefinic protons at δ_H_ [6.98 (dd, *J* = 12.0, 2.0 Hz), 6.53 (dd, *J* = 12.0, 2.0 Hz) and 6.37 (dd, *J* = 15.2, 4.8 Hz)], four oxygenated hydrogen groups at δ_H_ [5.06 (s), 4.66 (s), 4.03 (dd, *J* = 5.2, 4.8 Hz), and 3.59 (m)], one methine group at δ_H_ 3.35 (m), one methylene group at δ_H_ [4.40 (dd, *J* = 8.8, 7.6 Hz) and 3.95 (dd, *J* = 8.8, 2.4 Hz)], two methyl groups at δ_H_ 1.18 (d, *J* = 6.8 Hz) and δ_H_ 0.97 (d, *J* = 6.4 Hz)]. The ^13^C NMR data (Table 2), combined with DEPT 135° spectrum, displayed 11 carbon resonances, including one ester carbonyl at δ_C_ (171.2), four olefinic carbons at δ_C_ (145.6, 134.9, 129.7, and 124.5), two oxygenated methine carbons at δ_C_ (74.6 and 69.3), one oxygenated methylene carbon at δ_C_ (72.4), one methine carbon at δ_C_ (31.5), and two methyl carbons at δ_C_ (20.0 and 18.2). Using ^1^H–^1^H COZY correlations of H-3/H-4/H-12, H-6/H-7, and H-8/H-9/H-10/H-11, together with the key HMBC correlations from H-12 to C-3/C-5, H-3/H-6 to C-1, H-6 to C-4, H-8 to C-6, H-9 to C-5, H-11 to C-10/C-9, we established the planar structure of **1** (Figure 2). The relative configuration of the cyclohexanone moiety in **1** was deduced from the coupling constants and nuclear Overhauser effect spectroscopy (NOESY). The coupling constant of ^3^*J*_H − 6, H − 7_ = 12.0 Hz indicated that H-6 and H-7 have a *cis*-form of diaxial relationship. The NOESY correlations of H-6 with H-8 and H-8 with H-11 (Figure 3) pointed to the *Z* configuration of the double bond, with H-8 and H-11 lying on the same side of the molecular structure. The above results also indicated that the configurations of these two double bonds were 6*Z*, 8*Z*, respectively. The hydroxy groups at C-9 and C-10 were determined to be oriented at a threo configuration from the coupling constant (*J*) value of 5.2 Hz between H-9 and H-10 as previously described for asperochratide D and plecmillins G-H (Wang et al., 2016; Zou et al., 2020).

**Table 1:** ^1^H NMR spectroscopic data (400 MHz) (δ in ppm, *J* in Hz) for **1**–**6**.

**Position**	**1^a^**	**2^a^**	**3^b^**	**4^b^**	**5^a^**	**6^b^**
1			1.21, d (6.0)		1.47, d (6.8)	1.55, d (6.8)
2			3.84, td (6.4, 6.0)	2.53, d (3.6)	5.75, m	5.83, dd (11.2, 6.8)
3	4.40, dd (8.8, 7.6) 3.95, dd (8.8, 2.4)	4.39, dd (8.8, 7.6) 3.95, dd (8.8, 2.8)	1.60, m	2.48, m	6.02, dd (11.2, 2.0)	5.99, d (11.2)
4	3.35, m	3.43, m	2.26, m	2.03, m 1.74, m	6.27, m	
5			6.13, d (16.4)	4.40, m	7.15, d (8.8)	6.96, t (7.2)
6	6.98, dd (12.0, 2.0)	6.97, dd (11.2, 2.4)	6.81, m	2.74, m 2.47, m		2.24, dd (15.4, 7.2)
7	6.53, dd (12.0, 2.0)	6.49, dd (11.2,1.2)		1.56, m	6.26, d (8.8)	1.61, m
8	6.37, dd (15.2, 4.8)	6.40, dd (15.2, 4.8)	7.05, s	3.47, m	3.97, dd (8.8, 5.2)	3.53, m
9	4.03, dd (5.2, 4.8)	3.92, t (5.2)	5.03, d (6.8)	1.38, m	3.55, m	1.47, m
10	3.59, m	3.51, m		1.63, m 1.51, m	0.95, d (6.4)	0.93, t (7.2)
11	0.97, d (6.4)	1.05, d (6.4)	1.42, d (6.0)	2.38, t (7.2)		
12	1.18, d (6.8)	1.18, d (7.2)				
13				2.10, s		
9-OH	5.06, s	5.03, s				
10-OH	4.66, s	4.61, s				

^a^DMSO-*d*_6_, ^b^CDCl_3_.

**Table 2:** ^13^C NMR spectroscopic data (100 MHz) for **1**–**6**.

**Position**	**1^a^**	**2^a^**	**3^b^**	**4^b^**	**5^a^**	**6^b^**
1	171.2, C	171.7, C	23.7, CH_3_	210.2, C	14.9, CH_3_	15.2, CH_3_
2			67.6, CH	47.0, CH_2_	128.6, CH	130.4, CH
3	72.4, CH_2_	72.9, CH_2_	38.2, CH_2_	27.9, CH	124.2, CH	122.9, CH
4	31.5, CH	32.0, CH	29.8, CH_2_	31.6, CH_2_	143.1, CH	128.6, C
5	129.7, C	130.3, C	118.9, CH	70.0, CH	138.2, CH	146.4, CH
6	134.9, CH	135.5, CH	138.1, CH	47.2, CH_2_	127.3, C	26.3, CH_2_
7	124.5, CH	124.7, CH	129.4, C	37.5, CH_2_	126.5, CH	35.4, CH_2_
8	145.6, CH	146.9, CH	147.4, CH	67.2, CH	74.7, CH	72.9, CH
9	74.6, CH	75.6, CH	77.4, CH	36.1, CH_2_	69.4, CH	30.3, CH_2_
10	69.3, CH	70.1, CH	172.1, C	19.6, CH_2_	18.2, CH_3_	10.0, CH_3_
11	18.2, CH_3_	19.7, CH_3_	19.3, CH_3_	43.5, CH_2_	168.3, C	172.4, C
12	20.0, CH_3_	20.4, CH_3_		209.0, C		
13				29.9, CH_3_		

^a^DMSO-*d*_6_, ^b^CDCl_3_.

**Figure 4:**
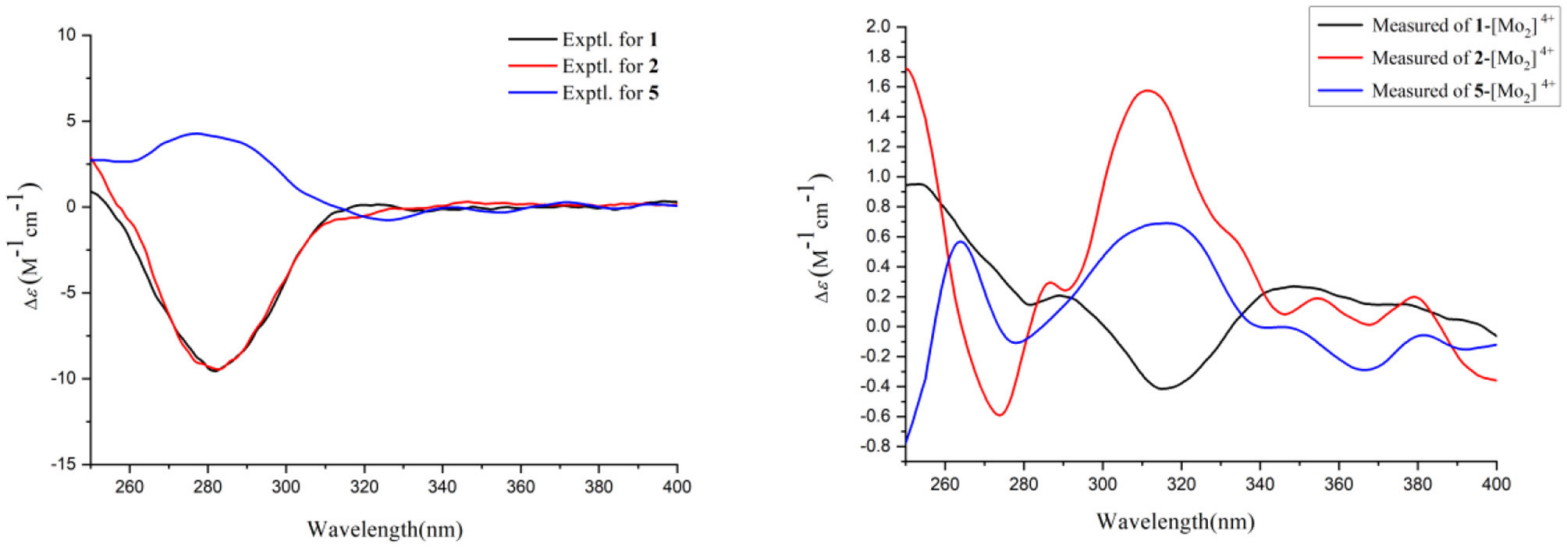
Experimental CD spectra of **1**–**2** and **5** and Experimental ECD spectra of the ${\text{Mo}}_{2}^{4+}$ complex of **1**–**2** and **5** with the inherent CD spectral subtracted.

**Figure 5:**
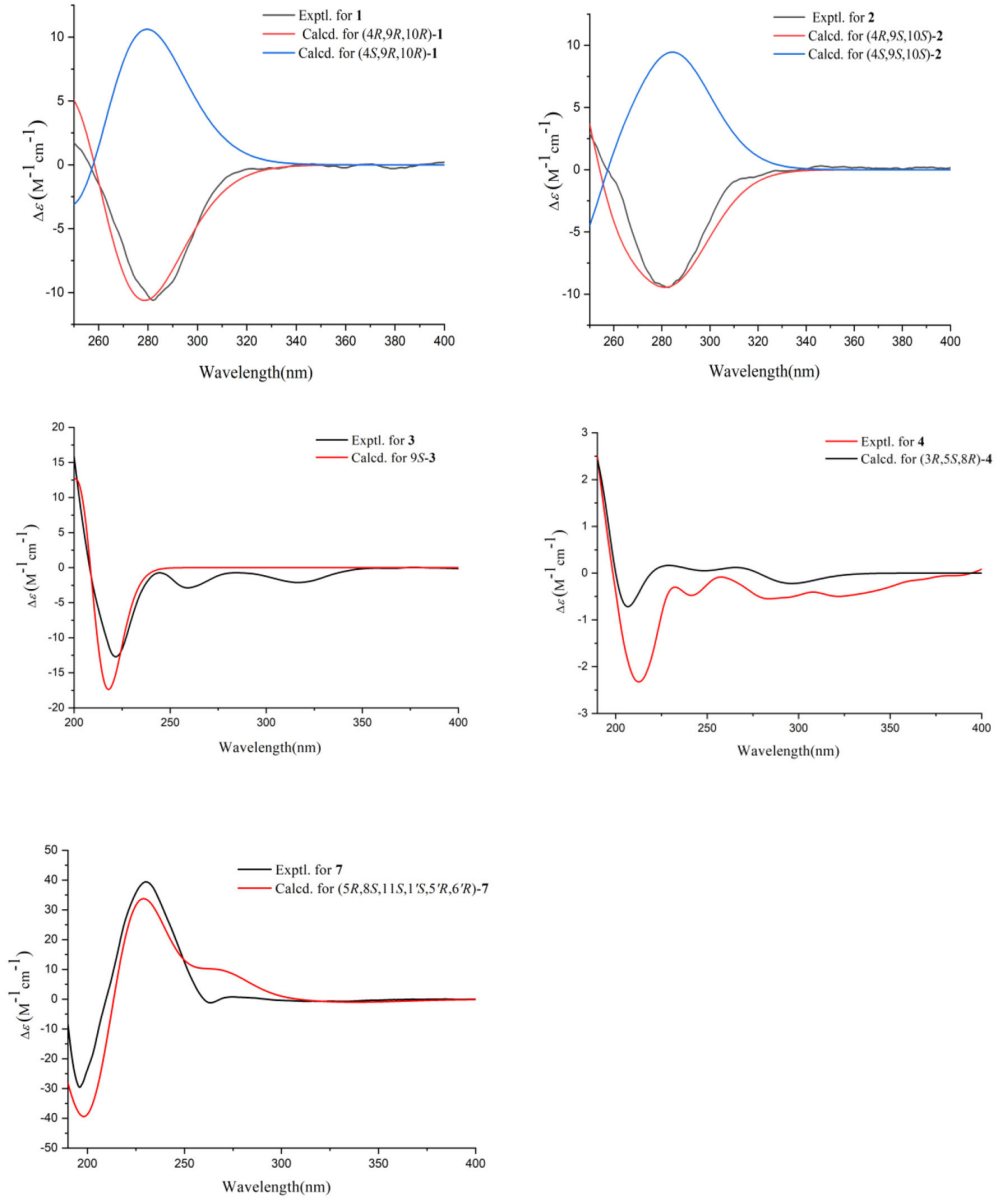
Experimental ECD spectral of **1**–**4** and **7**.

The absolute configurations of C-9 and C-10 in **1** was determined by the *in situ* dimolybdenum CD method developed by Snatzke and Frelek (Liu et al., 2010; Wang et al., 2013; Dewapriya et al., 2018; Bai et al., 2019c) (Figure 4). According to the empirical rule proposed in the Snatzke's method (Dewapriya et al., 2018), a metal complex having a 1,2-diol moiety and Mo_2_(OAc)_4_ was generated as an auxiliary chromophore after the addition of Mo_2_(OAc)_4_ to a DMSO solution of **1**. The observation of Cotton effect at ~300 nm in the induced spectrum originates from the chirality of the vic-diol, as exhibited by the O–C–C–O torsion angle in the favored conformation, which led to the assignment of the absolute configuration. A negative Cotton effect observed at 316 nm (Δε = −0.42) in the induced CD spectrum of **1** confirmed the 9*R*, 10*R* configurations (Figure 4). To determine the absolute configuration of **1**, the theoretical ECD spectra of two possible stereoisomers of 4*R*, 9*R*, 10*R* and 4*S*, 9*R*, 10*R* were created by the TDDFT calculations, and the calculated ECD curve of the isomer 4*R*, 9*R*, 10*R* was found to have a good agreement with the experimental one (Figure 5). Therefore, the absolute configuration of **1** was determined as 4*R*, 9*R*, 10*R*, and compound was named talarotone A.

The HR-ESI-MS data showed that compound **2** also has same molecular formula C_11_H_16_O_4_ as **1**. Analysis of 1D NMR data (Tables 1, 2) and HR-ESI-MS data suggested that **2** has a similar structure as **1** with the same carbon skeleton. A further investigation of **2** by DEPT135°, ^1^H-^1^H COZY, HMQC, and HMBC spectra established its planar structure, which is similar to **1**. The NOESY correlations of H-6/H-8 and H-8/H-11 (Figure 3), combined with the coupling constants of ^3^*J*_H − 6, H − 7_ = 11.2 Hz, pointed to the 6*Z*, 8*Z* configurations of the two double bonds. The coupling constants of H-8/H-9 (5.2 Hz) suggested that 9,10-diols are oriented at a threo configuration (Wang et al., 2016; Zou et al., 2020). Likewise, the absolute configurations of C-9 and C-10 in **2** were also determined using an *in situ* dimolybdenum CD method (Dewapriya et al., 2018; Bai et al., 2019a). The positive Cotton effect at 311 nm (Δε = +1.58) confirmed the 9*S*, 10*S* configurations for C-9 and C-10 (Figure 4). The absolute configuration at C-4 was determined to be *R* through TDDFT ECD calculation (Figure 5). Thus, the absolute configuration of **2** was 4*R*, 9*S*, 10*S*, and the compound was named talarotone B.

Compound **3** was isolated as a yellow oil. Using the HR-ESI-MS peak at *m/z* 197.1181 [M + H]^+^ (C_11_H_17_${\text{O}}_{3}^{+}$, calcd. for 197.1172), its molecular formula was determined to be C_11_H_16_O_3_ (four degrees of unsaturation). Its IR spectrum revealed the presence of hydroxyl group (3,475 cm^−1^), ester group (1,712 cm^−1^), and olefine group (1,636, 1,618 cm^−1^). The ^1^H and ^13^C NMR data (Tables 1, 2) of **3** suggested the presence of one ester carbonyl group at δ_C_ 172.1, two olefine groups at (δ_H_ 6.13, δ_C_ 118.9; δ_H_ 6.81, δ_C_ 138.1; δ_C_ 129.4; δ_H_ 7.05, δ_C_ 147.4), two oxygenated methine groups at (δ_H_ 3.84, δ_C_ 67.6; δ_H_ 5.03, δ_C_ 77.4), two methylene groups at (δ_H_ 1.60, δ_C_ 38.2; δ_H_ 2.26, δ_C_ 29.8), and two methyl groups at (δ_H_ 1.21, δ_C_ 23.7; δ_H_ 1.42, δ_C_ 19.3). The ^1^H–^1^H COZY correlations showed the fragments of H-1/H-2/H-3/H-4/H-5/H-6 and H-8/H-9/H-11, and on integrating it with the key HMBC correlations from H-1 to C-2/C-3, H-4 to C-2/C-6, H-5 to C-7, H-8 to C-6, H-9 to C-7/8/10/11 (Figure 2), the whole structure of **3** was arrived at. The C-5 and C-6 atoms in **3** were found to be oriented in a *trans* configuration, which was determined based on a large coupling constant of ^3^*J*_H − 5, H − 6_ = 16.4 Hz. The NOESY correlation of H-6 and H-8 indicated a *Z* configuration of the double bond (Figure 3). Mosher's method was used to determine the absolute configuration of C-2 (Bai et al., 2019a). Unfortunately, because of the excessive humidity in the environment, this reaction was unsuccessful and we did not have enough amount of the compound to perform this reaction again. The absolute configuration on C-9 was determined as *S* by ECD calculation (Figure 5). Therefore, **3** was identified as talarotone C.

Compound **4** was isolated as a yellow oil, with the molecular formula of C_13_H_20_O_3_ (four degrees of unsaturation), which was determined from its HR-ESI-MS data. The IR data showed absorption bands at 1,708, 1,638, 1,617 cm^−1^ indicating the presence of carbonyl group. The ^1^H NMR data (Table 1) of **4** revealed two oxygenated methine groups at [δ_H_ 4.40 (m) and 3.47 (m)], one methine group at δ_H_ 2.48 (m), one methyl group at δ_H_ 2.10 (s), seven methylene groups at [δ_H_ 2.74 (m) and 2.47 (m), δ_H_ 2.53 (d, *J* = 3.6 Hz), δ_H_ 2.38 (t, *J* = 7.2 Hz), δ_H_ 2.03 (m) and 1.74 (m), δ_H_ 1.63 (m) and 1.51 (m), δ_H_ 1.56 (m), δ_H_ 1.38 (m)]. The ^13^C NMR data (Table 2) consist of signals for two carbonyl groups at δ_C_ (210.2 and 209.0), two oxygenated methine groups at δ_C_ (70.0 and 67.2), one methine group at δ_C_ 27.9, one methyl group at δ_C_ 29.9, seven methylene groups at δ_H_ (47.2, 47.0, 43.5, 37.5, 36.1, 31.6, and 19.6). The ^1^H–^1^H COZY correlations suggested the presence of a 2-pentane and a butane fragment as H-6/H-5/H-4/H-3/H-2/H-7 and H-8/H-9/H-10/H-11 in **4** (Figure 2). The key HMBC correlations from H-6/H-2 to C-1, H-2/6/7 to C-4, H-2/9 to C-7, H-5 to C-3, H-8 to C-5, H-11 to C-9, H-11/13 to C-12 and H-13 to C-11 led to the confirmation of a planar structure of **4** (Figure 2). The relative configuration of **4** was determined from NOESY and 1D NOE spectra. The irradiation of H-5 resulted in the enhancement of H-3 in the selective 1D NOE spectrum. This observation, combined with the correlations of H-5/H-8 to H-3 in the NOESY spectrum (Figure 3), indicated that H-5, H-8, and H-3 were placed on the same side of the molecule. The absolute configuration of **4** was determined as 3*R*, 5*S*, 8*R* by comparing the experimental and calculated ECD spectra using TDDFT (Figure 5). Based on these findings, the structure of **4** was named talarotide A.

Compound **5** was isolated as a yellow oil and its molecular formula of C_11_H_16_O_4_ (four degrees of unsaturation) was from determined from its HR-ESI-MS data. The IR spectrum of **5** exhibited absorptions at 3,514, 3,443, 1,711, and 1,618 cm^−1^, corresponding to the hydroxyl group, ester group, and olefine group, respectively. The ^1^H NMR data (Table 1) of **5** exhibited five olefinic protons at δ_H_ [7.15 (d, *J* = 8.8 Hz), 6.27 (m), 6.26 (d, *J* = 8.8 Hz), 6.02 (dd, *J* = 11.2, 2.0 Hz) and 5.75 (m)], two oxygenated methine groups at δ_H_ [3.97 (dd, *J* = 8.8, 5.2 Hz) and 3.55 (m)], two methyl groups at δ_H_ [1.47 (d, *J* = 6.8 Hz) and δ_H_ 0.95 (d, *J* = 6.4 Hz)]. The ^13^C NMR data (Table 2), combined with the DEPT data, displayed 11 resonances for one carboxyl carbon at δ_C_ 168.3, six olefinic carbons at δ_C_ (143.1, 138.2, 128.6, 127.3, 126.5, and 124.2), two oxygenated methine groups at δ_C_ (74.7 and 69.4), and two methyl groups at δ_C_ (18.2 and 14.9). The analysis of the COZY correlations revealed the presence of two structural fragments as CH_3_(10)-CH(9)-CH(8)-CH(7) and CH(5)-CH(4)-CH(3)-CH(2)-CH_3_(1) (Figure 2). The linkages of these two fragments were elucidated by the HMBC correlations of H-1 to C-3, H-3/H-7 to C-5, H-7 to C-9 and H-10 to C-8 (Figure 2). The coupling constant (^3^*J*_H − 2, H − 3_ = 11.2 Hz and ^3^*J*_H − 4, H − 5_ = 8.8 Hz) indicated that the corresponding protons are positioned on the same side. The NOESY correlations from H-7 to H-5, H-1 to H-4 indicated that the three double bonds have a 2*Z*, 4*Z*, 6*Z* configuration (Figure 3). The coupling constant analysis (^3^*J*_H − 7, 8_ = 5.2 Hz) showed that **5** has a threo-8,9-diol configuration. Similar to compounds **1** and **2**, the absolute configurations of C-8 and C-9 in **5** were also assigned using an *in situ* dimolybdenum CD method (Dewapriya et al., 2018; Trang et al., 2022). The induced positive Cotton effect at 316 nm (Δε = +0.69), which indicates the O–C–C–O torsion angle, was consistent with positive helicity, which demonstrated the 8*S*, 9*S* configurations for **5** (Figure 4). The compound was named talaroyene A.

**Figure 6:**
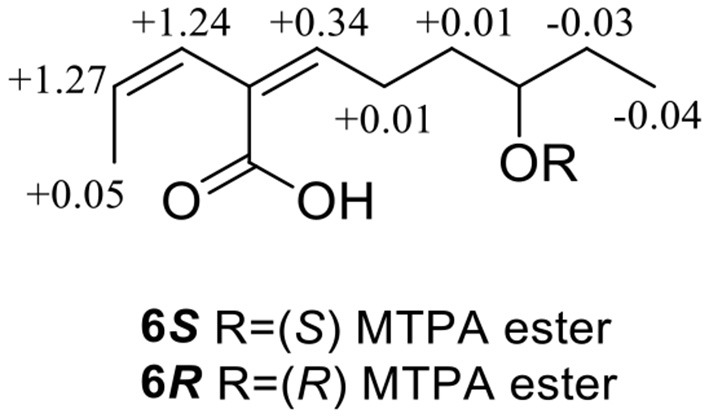
Δδ (= δ_*S*_ –δ_*R*_) values for (*S*)- and (*R*)-MTPA esters of **6**.

Compound **6** was obtained as a yellow oil, with a molecular formula of C_11_H_18_O_3_ (three degrees of unsaturation) as deduced from HR-ESI-MS data [*m/z* 197.1181 [M - H]^−^, (C_11_H_17_${\text{O}}_{3}^{-}$), calcd. for 197.1172]. The hydroxyl group (3,428 cm^−1^) and olefine group (1,692 and 1,619 cm^−1^) were observed in the IR spectrum. The ^1^H-NMR data (Table 1) showed three olefinic groups at δ_H_ [6.96 (t, *J* = 7.2 Hz), 5.99 (d, *J* = 11.2 Hz) and 5.83 (dd, *J* = 11.2, 6.8 Hz)], one oxygenated methine group at δ_H_ 3.53 (m), three methylene groups at δ_H_ [2.24 (td, *J* = 15.4, 7.2 Hz), 1.61 (m) and 1.47 (m)], two methyl groups at δ_H_ [1.55 (d, *J* = 6.8 Hz) and 0.93 (t, *J* = 7.2 Hz)]. The ^13^C-NMR data (Table 2) displayed 11 resonances, including one ester carbonyl carbon at δ_C_ 172.4, four olefinic carbons at δ_C_ (146.4, 130.4, 128.6, and 122.9), one oxygenated methine carbon at δ_C_ 72.9, three methylene carbons at δ_C_ (35.4, 30.3, and 26.3) and two methyl carbons at δ_C_ (15.2 and 10.0). The ^1^H–^1^H COZY spectrum of H-1/H-2/H-3 and H-5/H-6/H-7/H-8/H-9/H-10, combined with the key HMBC correlations from H-1 to C-3, H-2 to C-4, H-3 to C-5, H-5 to C-4/C-7/C-11, H-6 to C-4/C-8, H-7 to C-9 and H-10 to C-8, fully supported the structural connection of **6** (Figure 2). These 2D NMR data fully support the structural fragment of **6** containing a 6-hydroxy-2-propenyl-2-octenoic acid. The coupling constant of ^3^*J*_H − 2, H − 3_ = 11.2 Hz, combined with the NOESY correlation of H-3 with H-5 (Figure 3), pointed to the (2*Z*, 4*Z*) configuration of the double bonds. Mosher's method revealed the absolute configuration of C-8 in **6** was *R* (Bai et al., 2019a) (Figure 6). Thus, the absolute configuration of **6** was defined as 2*Z*, 4*Z*, 8*R*, and the compound was named talaroyene B.

Compound **7** was isolated as a white powder. Its molecular formula was deduced as C_26_H_32_O_7_ (11 degrees of unsaturation) from the HR-ESI-MS spectral data. The ^1^H and ^13^C NMR data (Table 3) revealed that **7** had a austin meroterpenoid skeleton and also a similar structure to brasilianoid G (Zhang et al., 2019). The obvious differences though were the presence of one methoxyl group at [δ_H_ 3.57 (s), δ_C_ 56.3 (CH_3_)] and one methyl group at [δ_H_ 1.62 (s), δ_C_ 15.8 (CH_3_)], and the absence of an aldehyde group at [δ_H_ 9.44 (s), δ_C_ 199.9 (C)] in **7**. The key HMBC correlations from H-11 to C-9/11-OMe, H-13 to C-5/C-9/C-10 pointed out that the aldehyde group for C-11 in brasilianoid G was replaced by the methoxyl group in **7**, and the double bond at [δ_H_ 5.24 (brs) and 5.74 (brs), δ_C_ 128.8 (CH_2_)] for C-13 in brasilianoid G was replaced by a methyl group in **7**. The ^1^H–^1^H COZY and HMBC spectra established the complete structure of **7** (Figure 2). The ROESY correlations of H-1 with H-9′/11-OMe, H-9′ with H-15, and H-12 with H-14, and H-14 with H-7′, confirmed the relative configuration of **7** (Figure 3). The absolute configuration of **7** was determined as 5*R*, 8*S*, 11*S*, 1′*S*, 5′*R*, 6′*R* by ECD quantum chemical calculations (Figure 5). Thus, the structure of **7** was named talaropenoid A.

**Table 3:** ^1^H and ^13^C NMR spectroscopic data (400/100 MHz) for **7** in CDCl_3_.

**Position**	**δ_H_**	**δ_C_**
1	6.65, d (9.6)	146.5, CH
2	6.11, d (9.6)	120.3, CH
3		164.3, C
4		85.7, C
5		45.6, C
6	1.58, m	25.6, CH_2_
7	1.68, m	26.0, CH_2_
8		43.2, C
9		136.7, C
10		138.4, C
11	5.42, s	99.2, CH
11-OMe	3.57, s	56.3, CH_3_
12	1.10, s	23.1, CH_3_
13	1.62, s	15.8, CH_3_
14	1.33, s	23.2, CH_3_
15	1.40, s	25.8, CH_3_
1′		62.8, C
2′		160.2, C
3′		138.4, C
4′		199.8, C
5′	4.23, q (6.4)	78.0, CH
6′		84.4, C
7′	2.15, s	15.5, CH_3_
8′	1.84, s	8.8, CH_3_
9′	1.52, d (6.8)	14.2, CH_3_
10′		172.4, C

On comparing the physical and spectroscopic data with the literature, the 13 known compounds, consisting of 11 meroterpenoids, one lianene, and one alkaloid, were identified as preaustinoid A2 (**8**) (Geris dos Santos and Rodrigues-Fo, 2003), asperaustin C (**9**) (Wen et al., 2019), 7-hydroxyde-hydroaustin (**10**) (Arunpanichlert et al., 2015), dehydroaustin (**11**) (Hayashi et al., 1994), austin (**12**) (Hayashi et al., 1994), 11β-acetoxyisoaustinone (**13**) (Arunpanichlert et al., 2015), furanoaustinol (**14**) (Park et al., 2018), dehydroaustinol (**15**) (Marquez-Fernandez et al., 2007), austinolide (**16**) (Fill et al., 2007), brasilianoid B (**17**) (Zhang et al., 2018), preaustinoid D (**18**) (Duan et al., 2016), pinophol D (**19**) (Luo et al., 2021), and alteamide (**20**) (Wang et al., 2021).

**Scheme 1:**
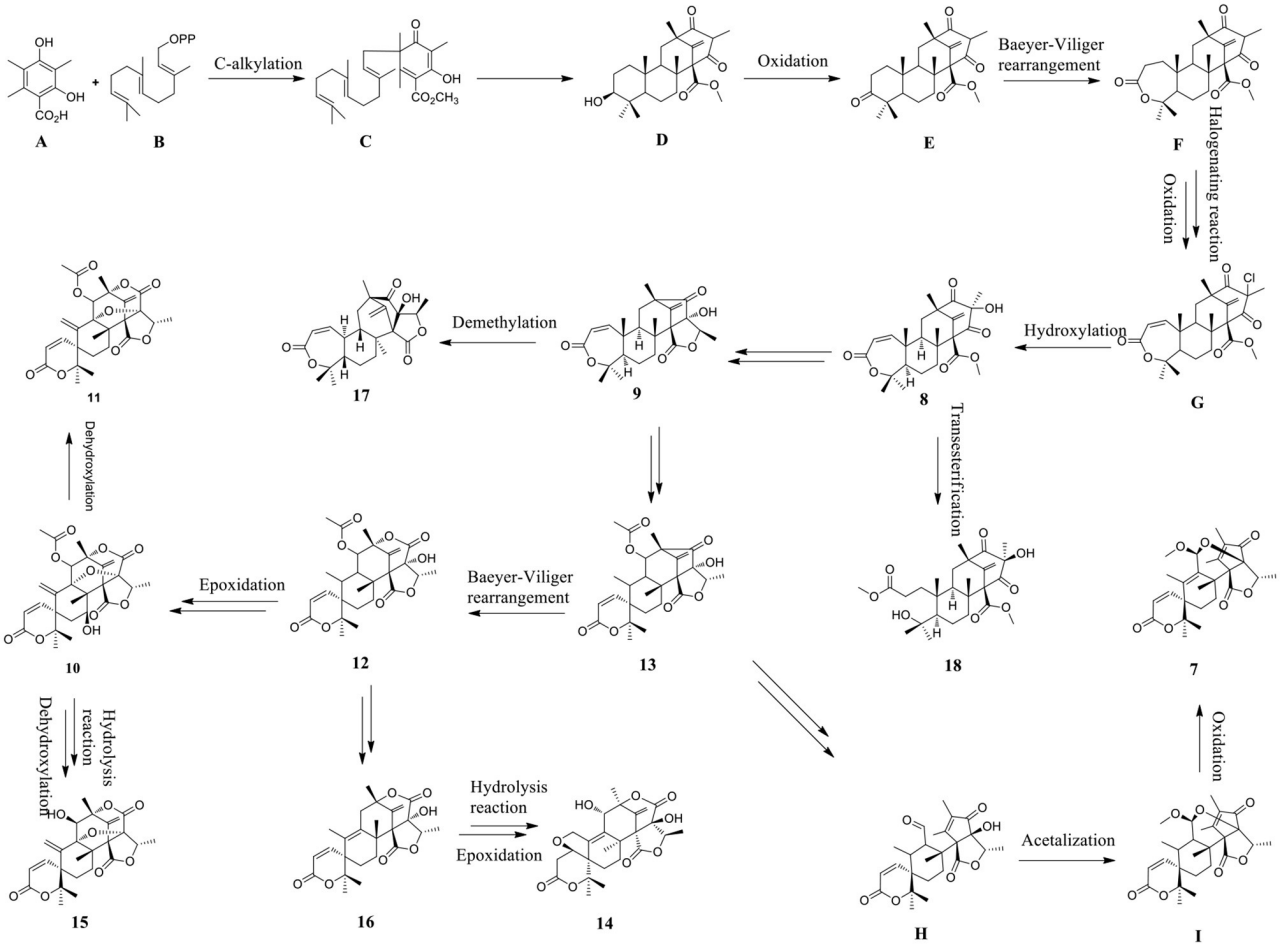
Plausible biosynthetic pathways of compounds **7**–**18**.

The plausible biosynthetic pathways for autsin meroterpenoid derivatives **7**–**18** were proposed (Scheme 1). Austin meroterpenoids were synthesized through the polyisoprene pathway using the precursor farnesyl diphosphate (B) by the alkylation of intermediate 3,5-dimethylthiacyllic acid (A), resulting in the formation of an intermediate (C) (Arunpanichlert et al., 2015). A series of meroterpenoid precursors (D–G) were synthesized based on the intermediate (C), and compound **8** was produced by the hydroxylation of precursor (G). Compound **8** was converted into **9**, **13**, and **17** and **18** by demethylation, transesterification, and Baeyer–Villiger rearrangement reaction, respectively. Compound **13** serves as the starting point for the formation of **7**, **10**–**12**, and **14**–**16** through Baeyer–Villiger rearrangement, epoxidation, oxidation, dehydroxylation, hydrolysis, and aldol condensation reaction, respectively.

Compounds **5** and **20** were found exhibit a strong antioxidant activity with IC_50_ values of 0.40 and 1.36 mM, respectively, while the IC_50_ value of the positive control trolox is 0.29 mM.

Compounds **3**, **6**, **11**, and **16** and **17** showed cytotoxic effects on Hela and A549 human cancer cells, with their IC_50_ values ranging from 28.89 to 62.23 μM (Table 4). All compounds exhibited no activity against the gastric cancer cell line RKO even at a concentration of 100 μM.

**Table 4:** The cytotoxic activity for compounds **3**, **6**, **11**, and **16** and **17** (IC_50_ in μM).

**Compound**	**Hela cell lines**	**A549 cell lines**
**3**	62.23 ± 0.23	
**6**	57.14 ± 0.15	
**11**		28.89 ± 0.37
**16**		2.73 ± 0.65
**17**	34.72 ± 0.84	
Adriamycin hydrochloride^a^	3.16 ± 0.024	2.56 ± 0.012

^a^Adriamycin hydrochloride was used as a positive control.

Compounds **7**, **10**–**12**, and **14**–**18** displayed moderate or strong anti-insect activity against newly hatched larvae of *H. armigera* Hubner, with their IC_50_ values ranging from 50 to 200 μg/mL (Table 5), while the IC_50_ value of the positive control azadirachtin is 50 μg/mL. Other compounds showed no growth inhibition activity against newly hatched larvae of *H. armigera* Hubner even at a concentration of 200 μg/mL.

**Table 5:** The anti-insect activity of compounds **7**, **10**–**12**, and **14**–**18**.

**Compound**	**7**	**10**	**11**	**12**	**14**	**15**	**16**	**17**	**18**	**Azadirachtin^a^**
IC_50_ (μg/mL)	100	50	100	50	200	50	200	100	100	50

^a^Azadirachtin was used as a positive control.

Structure-activity relationships of antiinsect activity against the newly hatched larvae of *H. armigera* Hubner with regard to interaction with meroterpenoids has been discussed. The substitution of hydroxyl and acetyl groups and the ether ring moiety on the skeleton of meroterpenoids influences their anti-insect activity. Compound **10**, which has a hydroxyl group on C-7, exhibits a better anti-insect activity superior to that of **11**, suggesting the hydroxyl group at C-7 contributes to an increased growth inhibition potency. The growth inhibition activity data of **12** and **13** indicates that the central ether ring moiety was a non-essential functional group for anti-insect activity in the structure of meroterpenoids. Compound **15**, which has a hydroxyl group on C-13, showed a higher anti-insect activity than compounds **11** and **16**, suggesting that 13-OH group enhances anti-insect activity, and the acetylation of 13-OH decreases the anti-insect activity.

The antibacterial activity of all compounds was assessed against *Staphylococcus aureus, Staphylococcus epidermidis, Pseudomonas aeruginosa, Escherichia coli*, and *Ralstonia solanacearum*. Compound **18** displayed a weak antibacterial activity against *R. solanacearum* even at an MIC value of 50 μg/mL, while the MIC value of the positive control streptomycin is 12.5 μg/mL. Other compounds showed no antibacterial activity against the bacterial species tested even at a concentration of 100 μg/mL.

These results suggest that anstin meroterpenoids are capable of contributing to the development of novel biopesticides such as microbial insecticides and antibiotics.

## 3 Materials and methods

### 3.1 General experimental procedures

The melting points of the isolated compounds were determined on a WRX-4 micromelting point apparatus (Shanghai YiCe Apparatus and Equipment Co., Ltd., Shanghai, China). CD spectra of the compounds were recorded on a Mos-500 spectrometer. IR spectra were recorded on a Thermo Nicolet 6700 (using KBr disks) spectrophotometer. PERSEE TU-1990 spectrophotometer was used for recording the UV spectra. Optical rotations were measured using a JASCO P-1020 digital polarimeter (JASCO, Tokyo, Japan). 1D and 2D NMR spectra were recorded from a Bruker AV spectrometer (400 MH_Z_ for ^1^H and 100 MH_Z_ for ^13^C) and a JNM-ECZS spectrometer (600 HM_Z_ for ^1^H and 150 MH_Z_ for ^13^C). HR-ESI-MS spectra were obtained from a Q-TOF Ultima Global GAA076 LC mass spectrometer. ESI-MS spectra were recorded on a MAT-95-MS mass spectrometer. Agilent 1100 prep-HPLC system with an Agilent C18 analytical (9.4 × 250 mm, 5 μm) HPLC column was utilized for performing high-performance liquid chromatography (HPLC). Silica gel (100–200 and 200–300 mesh, Qingdao Marine Chemical Factory, Qingdao, China) were employed in column chromatography (CC) and Sephadex LH-20 gel column (Amersham Blosclences manage) were used for recording CC. Biological activities were tested in ultra-clean workbench (Suzhou Sujing Company) and these results were tested with a full wavelength multifunctional microplate reader (BioTek, USA). Methanol, ethyl acetate, petroleum ether, chloroform, dimethyl sulfoxide, and other conventional chemical reagents used in the experimental investigations (Guangzhou Xilong Chemical Reagent Factory) (Cai et al., 2022).

### 3.2 Fungal materials

The fungus TGGP35 was isolated from the stem of the mangrove plant *Acanthus ilicifolius* and the sequence data have been deposited in GenBank (accession number MT071116). The fungal strain was identified as *Talaromyces flavus* (Eurotiales: Trichocomaceae) (Cai et al., 2022).

### 3.3 Fermentation, extraction, and isolation

The fungal strain TGGP35 was grown on solid rice cultures in 1 L Erlenmeyer flasks (100 flasks; 50 mL of rice and 1.0 gram of sodium bromide per Erlenmeyer flask, autoclave sterilization) at 28°C for 32 days. The fermentation was extracted three times with ethyl acetate (EtOAc), followed by vacuum concentration, thus generating EtOAc extracts weighing 90.7 g.

All the EtOAc extracts were subjected to silica gel column chromatography (CC) using a gradient elution of petroleum ether/EtOAc (*v/v*, gradient 100:0–0:100) and EtOAc/MeOH (*v/v*, gradient 100:0–70:30), which resulted in the separation of 15 fractions (Fr. A-Fr. O). Fr. L (30.9 g) was separated by silica gel CC (200–300 mesh) using a gradient elution of petroleum ether/EtOAc system (9:1–0:1) to obtain six fractions (Fr. L.1–Fr. L.6) by TLC analysis, and then subfraction Fr. L.2 was in semi-preparative HPLC (MeOH-H_2_O, 25:75, ν/ν) to obtain compounds **1** (14.3 mg), **2** (10.4 mg), **3** (12.1 mg), **8** (12.6 mg), and **18** (5.8 mg). Subfraction Fr. L.3 was further separated by semi-preparative HPLC (MeOH-H_2_O, 30:70, ν/ν) to obtain compound **6** (6.2 mg). Subfraction Fr. L.4 was further separated by semi-preparative HPLC (MeOH-H_2_O, 34:66, ν/ν) to provide compounds **4** (5.3 mg), **5** (4.2 mg), and **20** (5.7 mg). Fr. D2 (2.1 g) was put through a Sephadex LH-20 column (petroleum ether-CHCl_3_-MeOH, 2:1:1, *v/v*) and subjected to semi-preparative HPLC (MeOH-H_2_O, 60:40, ν/ν) to obtain compound **19** (6.2 mg). Fr. M (20.3 g) was separated by silica gel CC (200–300 mesh) using a gradient elution of petroleum ether/EtOAc system (5:1–0:1) to obtain six fractions (Fr. M.1-Fr. M.6), and then subfraction Fr. M.2 was further separated by semi-preparative HPLC (MeOH-H_2_O, 20:80, ν/ν) to generate compounds **7** (4.2 mg), **9** (5.9 mg), **12** (8.3 mg), **14** (13.1 mg), and **17** (6.8 mg). Subfraction Fr. M.3 was further separated by semi-preparative HPLC (MeOH-H_2_O, 10:90, ν/ν) to obtain compounds **10** (11.4 mg), **11** (3.5 mg), **13** (12.4 mg), **15** (10.5 mg), and **16** (7.6 mg).

### 3.4 Spectroscopic data

**Talarotone A** (**1**): yellow oil; ${\text{[α]}}_{\text{D}}^{25}$ −34.4 (*c* 0.10, MeOH); UV (MeOH) λ_max_ (log ε) 265, 218 nm; IR (KBr) ν_max_ 3,534, 3,426, 1,727, 1,627, 1,618 cm^−1^; CD (*c* 0.05, MeOH) λ_max_ (Δε) 281.4 (−9.67) nm; ^1^H and ^13^C NMR data see Tables 1, 2; HR-ESI-MS *m/z*: 235.0939 [M + Na]^+^, (C_11_H_16_O_4_Na^+^, calcd. for 235.0941).

**Talarotone B** (**2**): yellow oil; ${\text{[α]}}_{\text{D}}^{25}$ −32.4 (*c* 0.10, MeOH); UV (MeOH) λ_max_ (log ε) 268, 220 nm; IR (KBr) ν_max_ 3,521, 3,420, 1,717, 1,618, 1,384 cm^−1^; CD (*c* 0.05, MeOH) λ_max_ (Δε) 281 (−9.28) nm; ^1^H and ^13^C NMR data see Tables 1, 2; HR-ESI-MS *m/z*: 235.0939 [M + Na]^+^, (C_11_H_16_O_4_Na^+^, calcd. for 235.0941).

**Talarotone C** (**3**): yellow oil; ${\text{[α]}}_{\text{D}}^{25}$ +12.6 (*c* 0.10, MeOH); UV (MeOH) λ_max_ (log ε) 310, 221 nm; IR (KBr) ν_max_ 3,475, 1,712, 1,636, 1,618 cm^−1^; CD (*c* 0.05, MeOH) λ_max_ (Δε) 209 (−2.52) nm; ^1^H and ^13^C NMR data see Tables 1, 2; HR-ESI-MS *m/z*: 197.1181 [M + H]^+^, (C_11_H_17_${\text{O}}_{3}^{+}$, calcd. for 197.1172).

**Talarotide A** (**4**): yellow oil; ${\text{[α]}}_{\text{D}}^{25}$ −23.6 (*c* 0.10, MeOH); UV (MeOH) λ_max_ (log ε) 306, 219 nm; IR (KBr) ν_max_ 1,708, 1,638, 1,617 cm^−1^; CD (*c* 0.05, MeOH) λ_max_ (Δε) 245 (+3.93), 264 (−11.89) nm; ^1^H and ^13^C NMR data see Tables 1, 2; HR-ESI-MS *m/z*: 225.1473 [M + H]^+^, (C_13_H_21_${\text{O}}_{3}^{+}$, calcd. for 225.1465).

**Talaroyene A** (**5**): yellow oil; ${\text{[α]}}_{\text{D}}^{25}$ +18.4 (*c* 0.10, MeOH); UV (MeOH) λ_max_ (log ε) 218, 200 nm; IR (KBr) ν_max_ 3,514, 3,443, 1,711, 1,618, 1,385 cm^−1^; CD (*c* 0.05, MeOH) λ_max_ (Δε) 279.71 (+4.29) nm; ^1^H and ^13^C NMR data see Tables 1, 2; HR-ESI-MS *m/z*: 211.0937 [M – H]^−^, (C_11_H_15_${\text{O}}_{4}^{-}$, calcd. for 211.0965).

**Talaroyene B** (**6**): yellow oil; ${\text{[α]}}_{\text{D}}^{25}$ −24.2 (*c* 0.10, MeOH); UV (MeOH) λ_max_ (log ε) 307, 217 nm; IR (KBr) ν_max_ 3,428, 1,692, 1,619, 614 cm^−1^; ^1^H and ^13^C NMR data see Tables 1, 2; HR-ESI-MS *m/z*: 197.1181 [M – H]^−^, (C_11_H_17_${\text{O}}_{3}^{-}$, calcd. for 197.1172).

(*S*)-MTPA ester of **6**: ^1^H NMR (CDCl_3_, 600 MHz): δ_H_ 7.01 (1H, t, *J* = 7.2 Hz, H-5), 5.99 (1H, d, *J* = 12.0 Hz, H-3), 5.86 (1H, dd, *J* = 12.0, 6.8 Hz, H-2), 2.36 (2H, m, H-6), 1.63 (2H, m, H-7), 1.57 (3H, d, *J* = 6.6 Hz, H-1), 1.47 (2H, m, H-9), 0.95 (3H, d, *J* = 7.2 Hz, H-10); ESI-MS *m/z* 654.4 [M + H]^+^.

(*R*)-MTPA ester of **6**: ^1^H NMR (CDCl_3_, 600 MHz): δ_H_ 7.35 (1H, m, H-5), 7.22 (1H, d, *J* = 9.6 Hz, H-3), 7.13 (1H, dd, *J* = 9.6, 2.4 Hz, H-2), 2.37 (2H, m, H-6), 1.64 (2H, m, H-7), 1.62 (3H, d, *J* = 7.2 Hz, H-1), 1.43 (2H, m, H-9), 0.92 (3H, d, *J* = 7.2 Hz, H-10); ESI-MS *m/z* 692.1 [M + K]^+^.

**Talaropenoid A** (**7**): White powder; ${\text{[α]}}_{\text{D}}^{25}$ −22.6 (*c* 0.10, MeOH); mp. 182.3–188.7°C; UV (MeOH) λ_max_ (log ε) 245 nm; IR (KBr) ν_max_ 3,549, 3,413, 1,617, 1,613, 603 cm^−1^; CD (*c* 0.05, MeOH) λ_max_ (Δε) 230 (+39.65) nm; ^1^H and ^13^C NMR data see Tables 1, 2; HR-ESI-MS *m/z*: 445.0281 [M – H]^−^, (C_26_H_31_${\text{O}}_{7}^{-}$, calcd. for 455.2064).

### 3.5 Biological assays

#### 3.5.1 Antioxidant activity

The antioxidant activity assay was performed following a previously reported method (Zeng et al., 2022). The assay was performed in a 96-well microplate by adding 10 μL of the sample solution to 200 μL of ABTS working solution. Concentration gradients of 2.0, 1.0, 0.5, and 0.25 mg/mL were prepared for all the test groups, including a positive control. The blank control consisted of PBS buffer, DMSO served as the negative control, and Trolox was used as the positive control, with the IC_50_ value of 0.29 mM. The measurement of antioxidant effect was done using a full wavelength multifunctional microplate reader at a specific wavelength of 734 nm. The inhibition rate of each sample was calculated using the formula: inhibition rate = [(*A*_blank_ – *A*_compound_)/*A*_blank_]^*^100%. Finally, the IC_50_ value was calculated using the SPSS software.

#### 3.5.2 Cytotoxic activity

All compounds were assessed for their cytotoxic activity against three human cell lines (A549, Hela, RKO) using the MTT method (Mosmann, 1983). The sample concentrations were prepared in five gradient levels: 100, 80, 60, 40, and 20 μM. The experiment was repeated more than three times, and the experimental data were measured using a full-wavelength microplate reader with a test wavelength of 492 nm. DMSO was used as the negative control, and doxorubicin hydrochloride served as the positive control. The inhibition rate of each sample was calculated using the formula: inhibition rate = [(OD_compound_ – OD_DMSO_)/OD_DMSO_]^*^100%. The IC_50_ value was determined using GraphPad Prism software.

#### 3.5.3 Anti-insect activity

The growth inhibition activity against newly hatched larvae of *Helicoverpa armigera* Hubner was tested using methods from literature (Bai et al., 2019a). The tested compounds and the positive control azadirachtin were dissolved in DMSO at a concentration of 1 mg/mL. The activity was evaluated by adding serial dilutions of the tested compounds and azadirachtin (concentrations of 200, 100, 50, 25, and 12.5 μL/well) as an artificial diet for the newly hatched larvae. Each treatment was replicated three times, and the bioassay diet was placed in six-well plates. The larvae were then incubated at a controlled temperature of 25 ± 1°C at a relative humidity of 80%. DMSO was used as the negative control, azadirachtin was used as the positive control, and the artificial diet was used as the blank control. The mortality rate of the larvae was recorded on the 2nd, 4th, 6th, and 8th day after treatment.

#### 3.5.4 Antibacterial activity

The antibacterial activity of **1**–**20** was assessed against five pathogenic bacteria, including *Staphylococcus aureus, S. epidermidis, Escherichia coli, Pseudomonas aeruginosa*, and *Ralstonia solanacearum* by the microplate assay method (Pierce et al., 2008). The broth medium containing pathogenic bacteria was used as the blank group, DMSO as the negative control, and ciprofloxacin and streptomycin were used as positive control.

## 4 Conclusions

In summary, 20 secondary metabolites, including six new polyketides (**1**–**6**) and one new meroterpenoid (**7**), and 13 known compounds were isolated from mangrove-derived fungus *T. flavus* TGGP35. Compounds **5** and **20** demonstrated moderate antioxidant capability, with IC_50_ values of 0.40 and 1.36 mM, respectively. Compounds **3, 6**, **11**, and **16** and **17** exhibited weak cytotoxic activity on Hela and A549 human cancer cells, with IC_50_ values ranging from 28.89 to 62.23 μM. Compounds **7**, **10**–**12**, and **14**–**18** displayed moderate or significant anti-insect activity, with the IC_50_ values ranging from 50 to 200 μg/mL. Compound **18** showed antimicrobial activity against *R. solanacearum*, with an MIC value of 50 μg/mL. The biosynthetic pathway and structure–activity relationship with regard to the anti-insect activity of meroterpenoids were explained in detail.

## Data availability statement

The datasets presented in this study can be found in online repositories. The names of the repository/repositories and accession number(s) can be found in the article/Supplementary material.

## Ethics statement

Ethical approval was not required for the studies on humans in accordance with the local legislation and institutional requirements because only commercially available established cell lines were used. Ethical approval was not required for the studies on animals in accordance with the local legislation and institutional requirements because only commercially available established cell lines were used.

## Author contributions

JC: Data curation, Writing – original draft. XZho: Methodology, Writing – original draft. BW: Methodology, Writing – original draft. XZha: Methodology, Writing – original draft. ML: Methodology, Writing – original draft. LH: Methodology, Writing – original draft. RW: Methodology, Writing – original draft. YC: Methodology, Writing – original draft. XL: Methodology, Writing – original draft. YL: Methodology, Writing – original draft. GC: Methodology, Writing – original draft. FC: Validation, Writing – review & editing. GH: Validation, Writing – review & editing. CZ: Validation, Writing – review & editing.
