# Supplementary material for: Bioactive polyketides and meroterpenoids from the mangrove-derived fungus Talaromyces flavus TGGP35
Source: Front Microbiol. 2024 Feb 1;15:1342843. doi: 10.3389/fmicb.2024.1342843 (PMC10867163; doi:10.3389/fmicb.2024.1342843)
Supplement: Supplementary file 1 [file Data_Sheet_1.PDF]

frontiers

Supporting information

Bioactive polyketides and meroterpenoid from the mangrove-derived fungus *Talaromyces flavus* TGGP35

Jin Cai[1,2,†], Xueming Zhou[1,2,†], Bin Wang[1,2], Xuelong Zhang[1,2], Mengyao Luo[1,2], Longtao Huang[1,2], Ruoxi Wang[1,2], Yonghao Chen[1,2], Xiaoyang Li[1,2], Youping Luo[1,2], Guangying Chen[1,2], Fei Cao[3,*], Guolei Huang[1,2,*] and Caijuan Zheng[1,2,*]

[1]Key Laboratory of Tropical Medicinal Resource Chemistry of Ministry of Education, College of Chemistry and Chemical Engineering, Hainan Normal University, Haikou, Hainan, 571158, People's Republic of China

[2]Key Laboratory of Tropical Medicinal Plant Chemistry of Hainan Province, Haikou, Hainan, 571158, People's Republic of China

[3]Key Laboratory of Pharmaceutical Quality Control of Hebei Province, Key Laboratory of Medicinal Chemistry and Molecular Diagnostics of Education Ministry of China, College of Pharmaceutical Sciences, Hebei University, Baoding 071002, China

† These authors contributed equally to this work.

*Correspondence: caijuan2002@163.com (C.-J. Zheng); huangguolei1982@163.com (G.-L. Huang); caofei542927001@163.com (F. Cao)

List of contents

Figure S1. ^{1}H NMR spectrum of **1** in DMSO-d_6
Figure S2. ^{13}C NMR spectrum of **1** in DMSO-d_6
Figure S3. 135°-DEPT spectrum of **1** in DMSO-d_6
Figure S4. HMQC spectrum of **1** in DMSO-d_6
Figure S5. ^{1}H-^{1}H COSY spectrum of **1** in DMSO-d_6
Figure S6. HMBC spectrum of **1** in DMSO-d_6
Figure S7. NOESY spectrum of **1** in DMSO-d_6
Figure S8. HR-ESI-MS spectrum of **1**

Figure S9. ^{1}H NMR spectrum of **2** in DMSO-d_6
Figure S10. ^{13}C NMR spectrum of **2** in DMSO-d_6
Figure S11. 135°-DEPT spectrum of **2** in DMSO-d_6
Figure S12. HMQC spectrum of **2** in DMSO-d_6
Figure S13. ^{1}H-^{1}H COSY spectrum of **2** in DMSO-d_6
Figure S14. HMBC spectrum of **2** in DMSO-d_6
Figure S15. NOESY spectrum of **2** in DMSO-d_6
Figure S16. HR-ESI-MS spectrum of **2**

Figure S17. ^{1}H NMR spectrum of **3** in $CDCl_3$
Figure S18. ^{13}C NMR spectrum of **3** in $CDCl_3$
Figure S19. 135°-DEPT spectrum of **3** in $CDCl_3$
Figure S20. HMQC spectrum of **3** in $CDCl_3$
Figure S21. ^{1}H-^{1}H COSY spectrum of **3** in $CDCl_3$
Figure S22. HMBC spectrum of **3** in $CDCl_3$
Figure S23. NOESY spectrum of **3** in $CDCl_3$
Figure S24. HR-ESI-MS spectrum of **3**

Figure S25. ^{1}H NMR spectrum of **4** in $CDCl_3$
Figure S26. ^{13}C NMR spectrum of **4** in $CDCl_3$
Figure S27. 135°-DEPT spectrum of **4** in $CDCl_3$
Figure S28. HMQC spectrum of **4** in $CDCl_3$
Figure S29. ^{1}H-^{1}H COSY spectrum of **4** in $CDCl_3$
Figure S30. HMBC spectrum of **4** in $CDCl_3$
Figure S31. NOESY spectrum of **4** in $CDCl_3$
Figure S32. 1D NOE spectrum of **4** in $CDCl_3$
Figure S33. HR-ESI-MS spectrum of **4**

Figure S34. ^{1}H NMR spectrum of **5** in DMSO-d_6
Figure S35. ^{13}C NMR spectrum of **5** in DMSO-d_6

Figure S36. 135º-DEPT spectrum of **5** in DMSO-d_6
Figure S37. HMQC spectrum of **5** in DMSO-d_6
Figure S38. ^{1}H-^{1}H COSY spectrum of **5** in DMSO-d_6
Figure S39. HMBC spectrum of **5** in DMSO-d_6
Figure S40. NOESY spectrum of **5** in DMSO-d_6
Figure S41. HR-ESI-MS spectrum of **5**

Figure S42. ^{1}H NMR spectrum of **6** in $CDCl_3$
Figure S43. ^{13}C NMR spectrum of **6** in $CDCl_3$
Figure S44. 135º-DEPT spectrum of **6** in $CDCl_3$
Figure S45. HMQC spectrum of **6** in $CDCl_3$
Figure S46. ^{1}H-^{1}H COSY spectrum of **6** in $CDCl_3$
Figure S47. HMBC spectrum of **6** in $CDCl_3$
Figure S48. NOESY spectrum of **6** in $CDCl_3$
Figure S49. HR-ESI-MS spectrum of **6**
Figure S50. ^{1}H NMR ($CDCl_3$, 600 MHz) of *S*-MTPA ester of **6**
Figure S51. ESI-MS spectrum of *R*-MTPA ester of **6**
Figure S52. ^{1}H NMR ($CDCl_3$, 600 MHz) of *R*-MTPA ester of **6**
Figure S53. ESI-MS spectrum of *S*-MTPA ester of **6**

Figure S54. ^{1}H NMR spectrum of **7** in $CDCl_3$
Figure S55. ^{13}C NMR spectrum of **7** in $CDCl_3$
Figure S56. 135º-DEPT spectrum of **7** in $CDCl_3$
Figure S57. HMQC spectrum of **7** in $CDCl_3$
Figure S58. ^{1}H-^{1}H COSY spectrum of **7** in $CDCl_3$
Figure S59. HMBC spectrum of **7** in $CDCl_3$
Figure S60. ROESY spectrum of **7** in $CDCl_3$
Figure S61. HR-ESI-MS spectrum of **7**

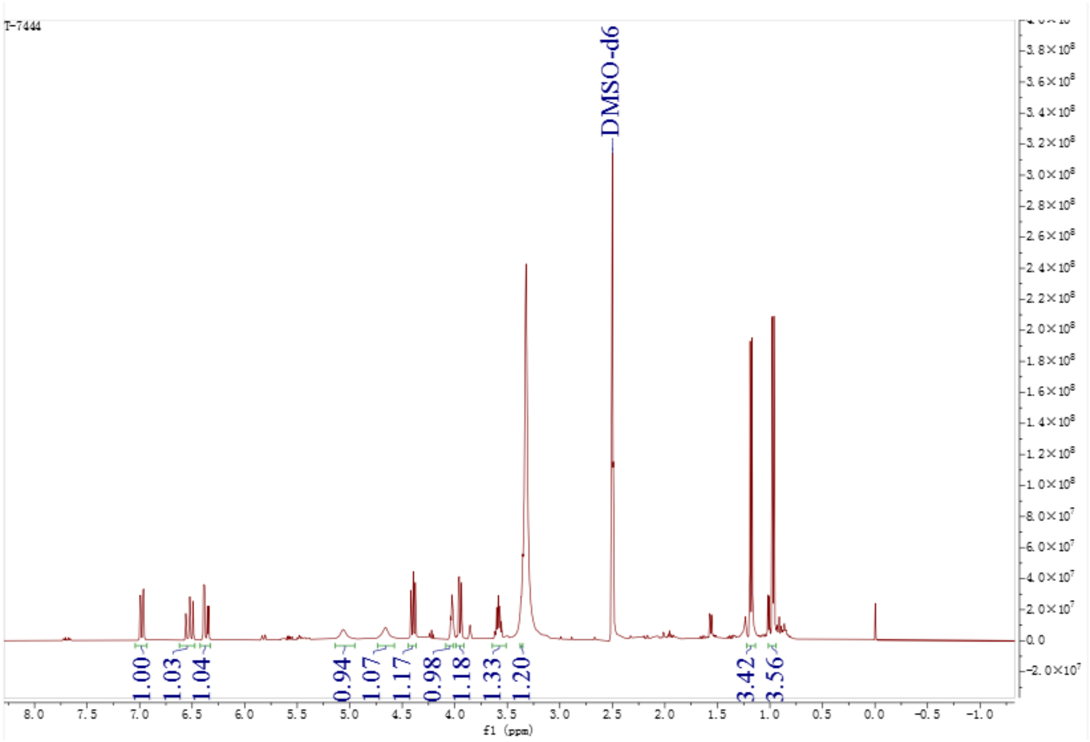

Figure S1. ^{1}H NMR spectrum of **1** in DMSO-d_6

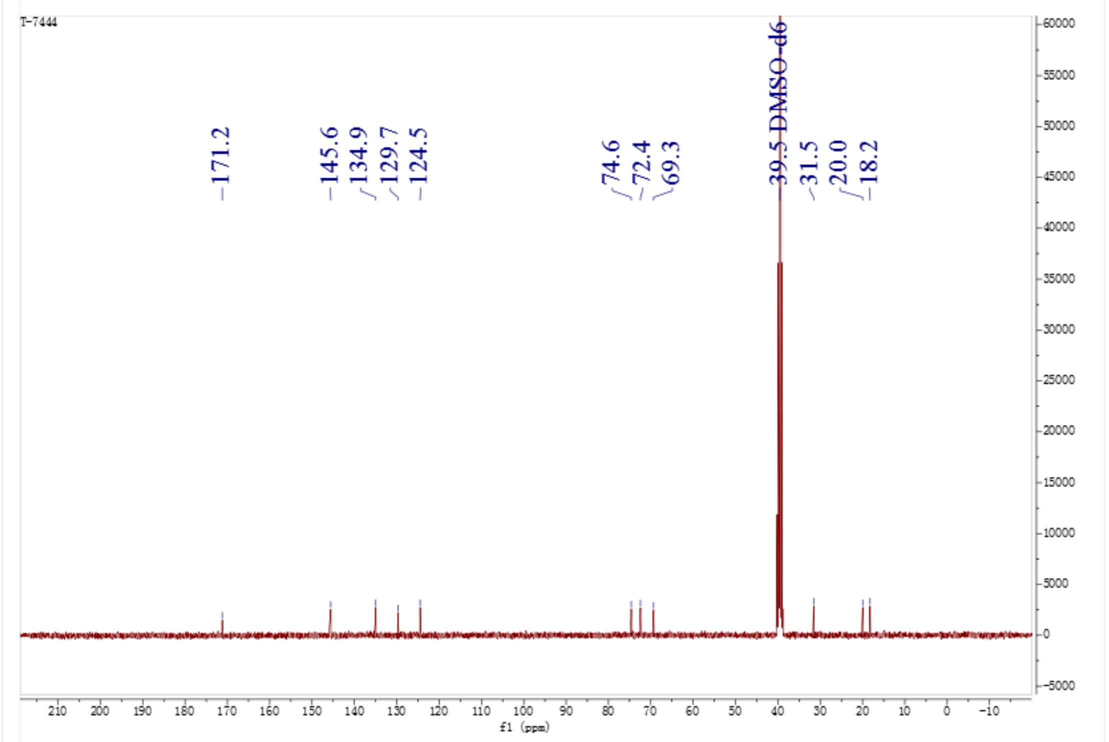

Figure S2. ^{13}C NMR spectrum of **1** in DMSO-d_6

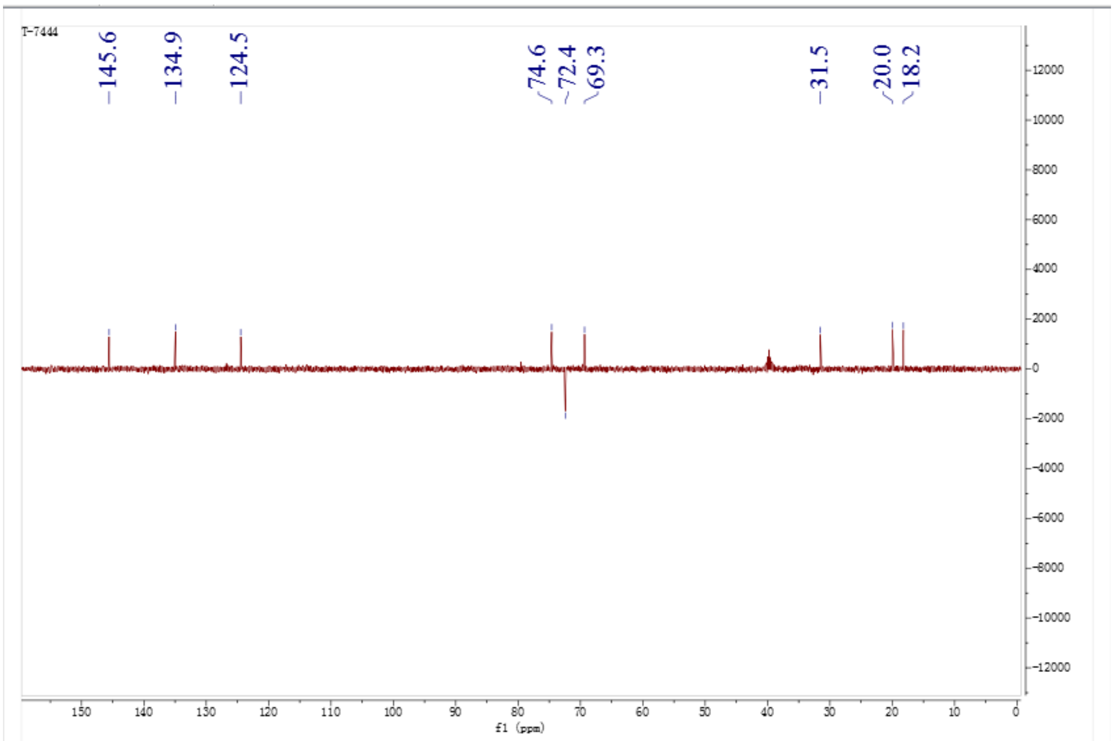

Figure S3. 135°-DEPT spectrum of **1** in DMSO-d_6

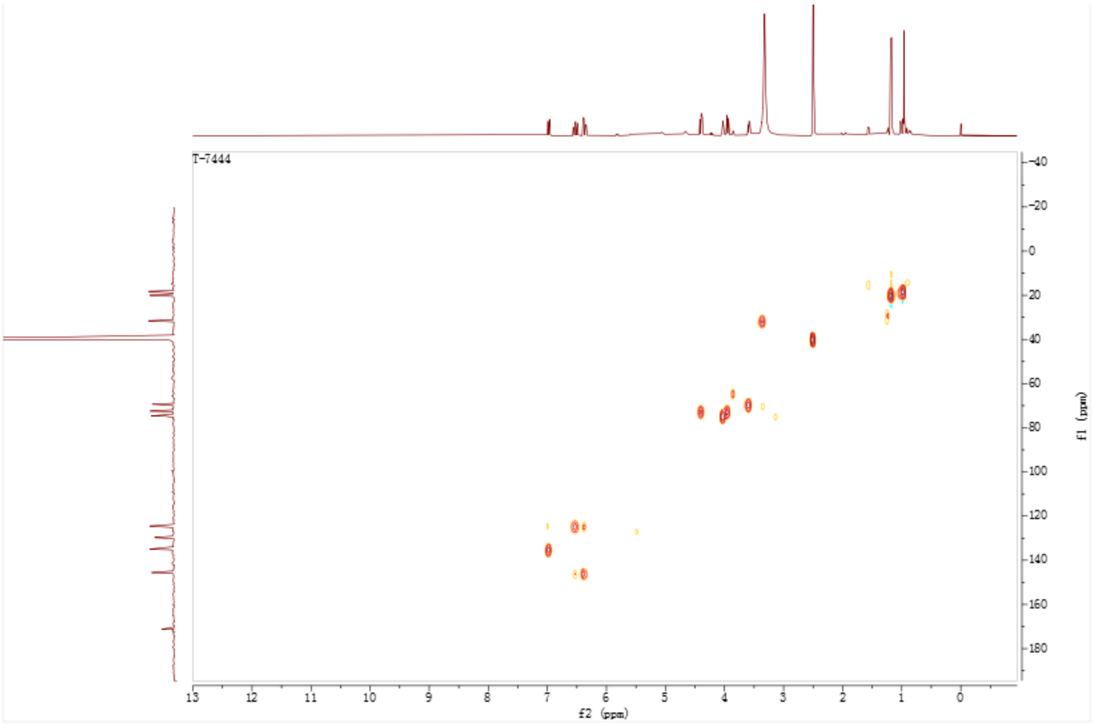

Figure S4. HMQC spectrum of **1** in DMSO-d_6

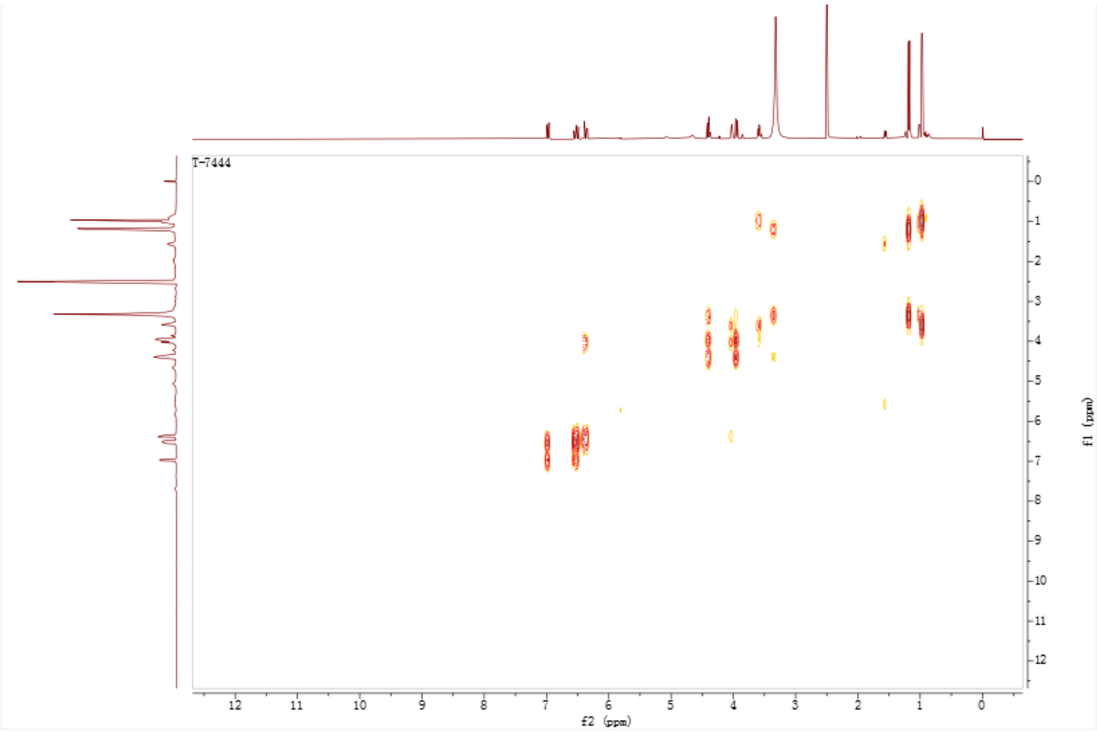

Figure S5. ^1H-^1H COSY spectrum of **1** in DMSO-d_6

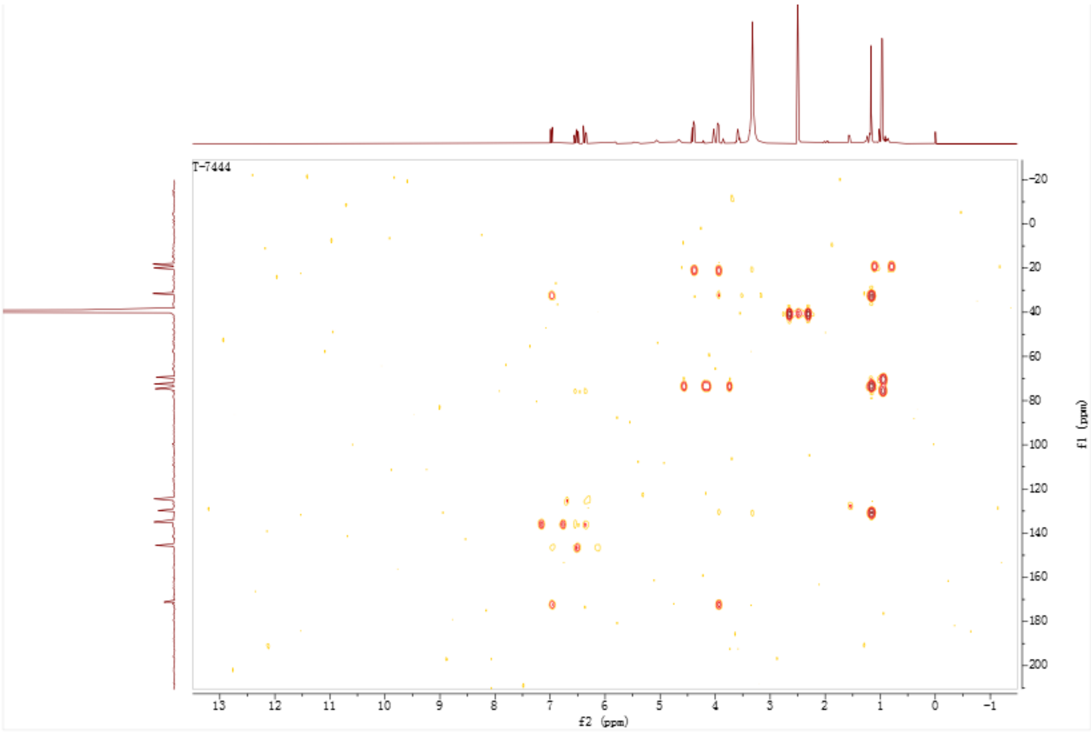

Figure S6. HMBC spectrum of **1** in DMSO-d_6

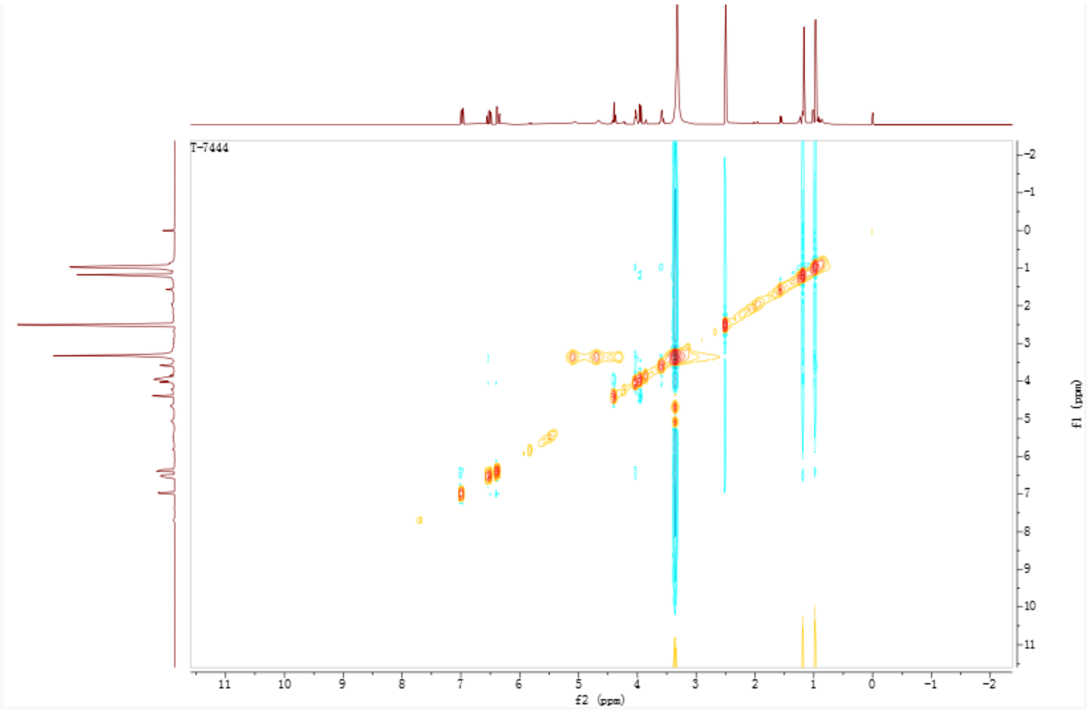

Figure S7. NOESY spectrum of **1** in DMSO-d_6

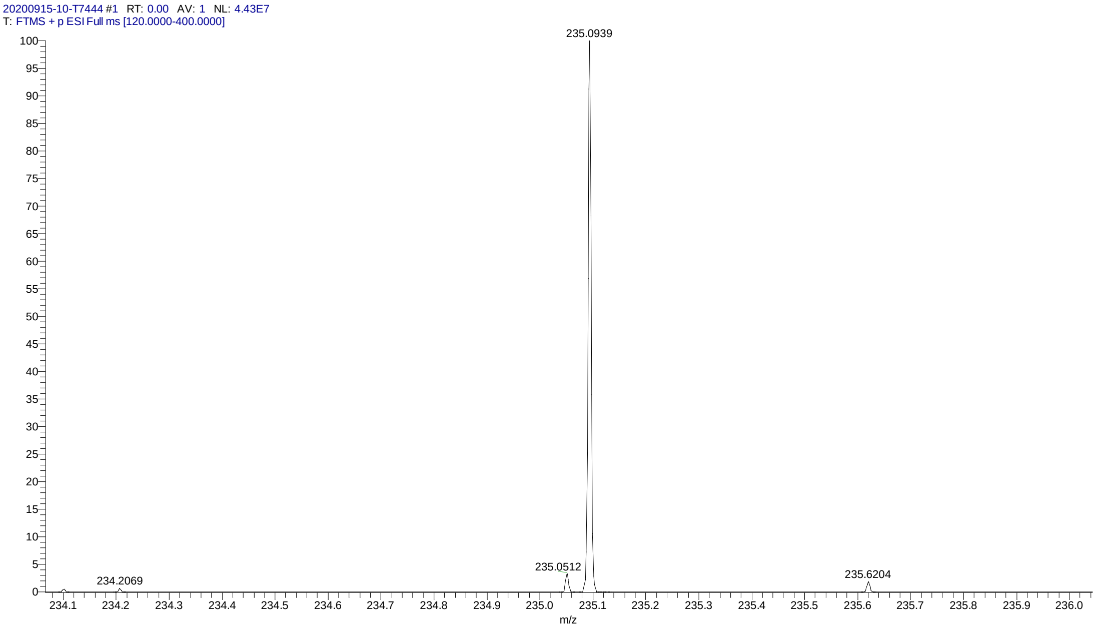

Figure S8. HR-ESI-MS spectrum of **1**

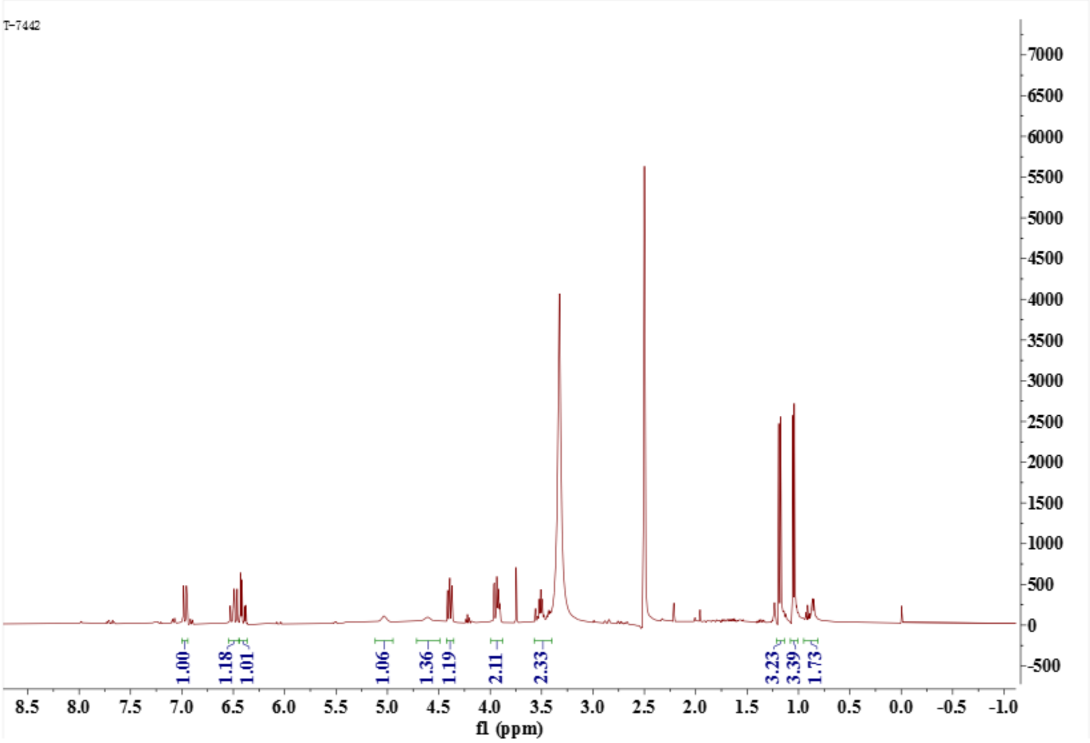

Figure S9. ^{1}H NMR spectrum of **2** in DMSO-d_6

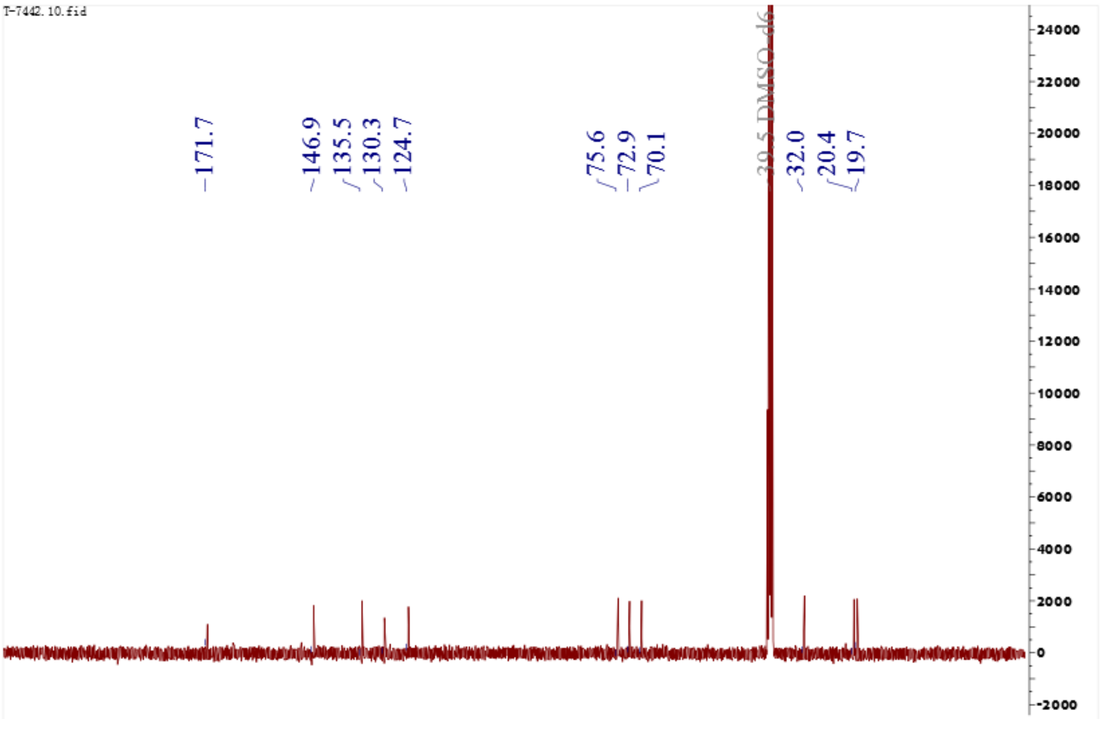

Figure S10. ^{13}C NMR spectrum of **2** in DMSO-d_6

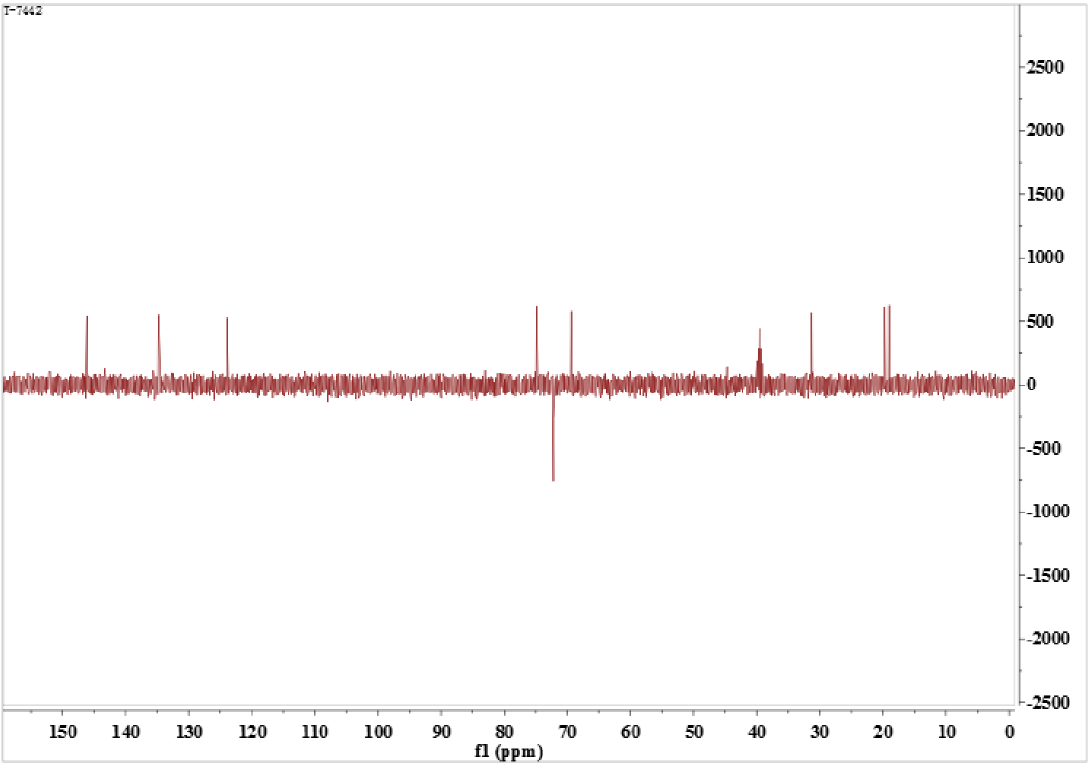

Figure S11. 135°-DEPT spectrum of **2** in DMSO-d_6

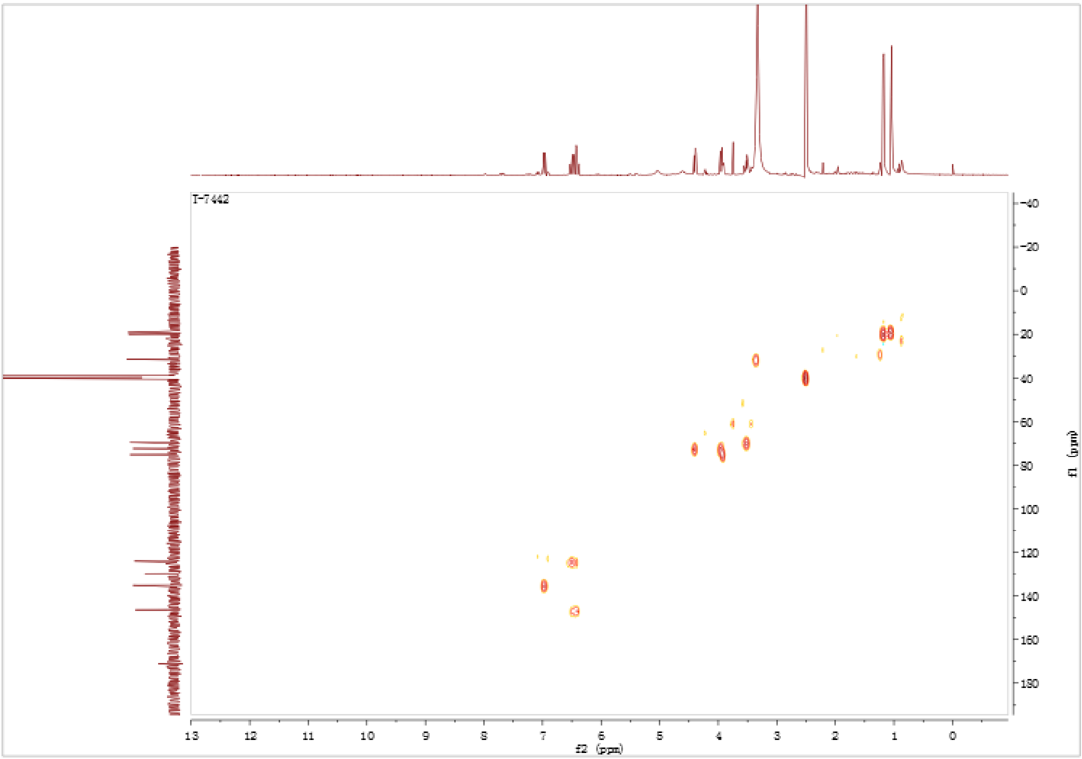

Figure S12. HMQC spectrum of **2** in DMSO-d_6

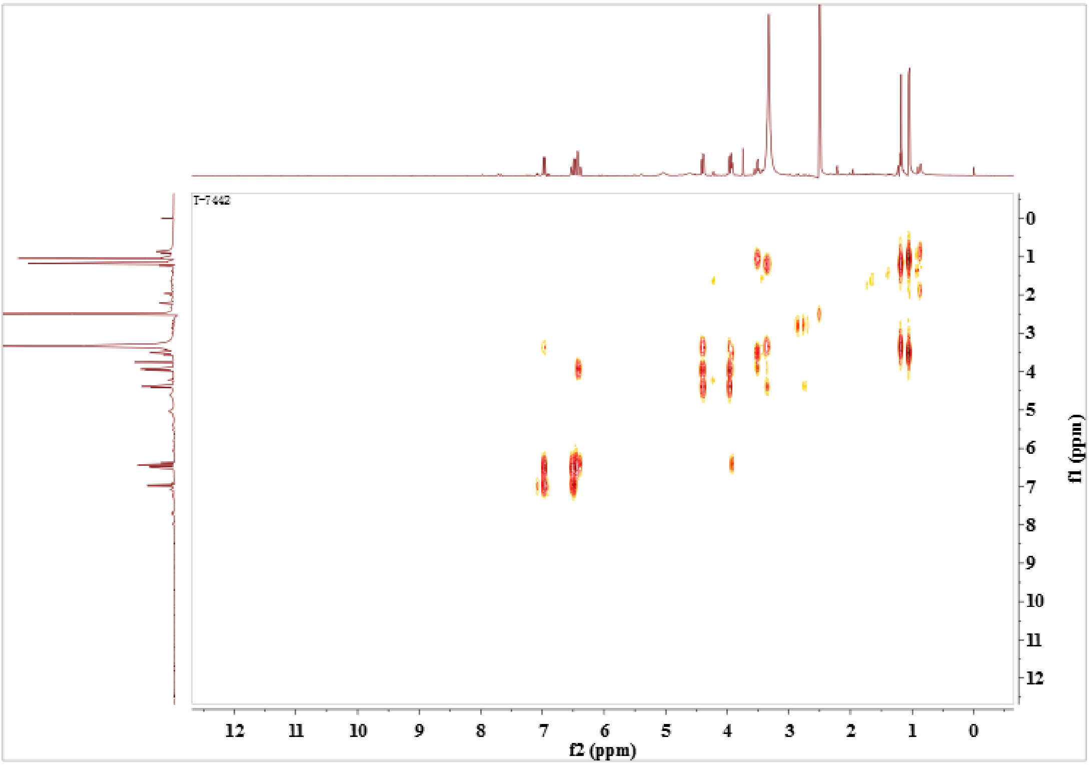

Figure S13. ^1H-^1H COSY spectrum of **2** in DMSO-d_6

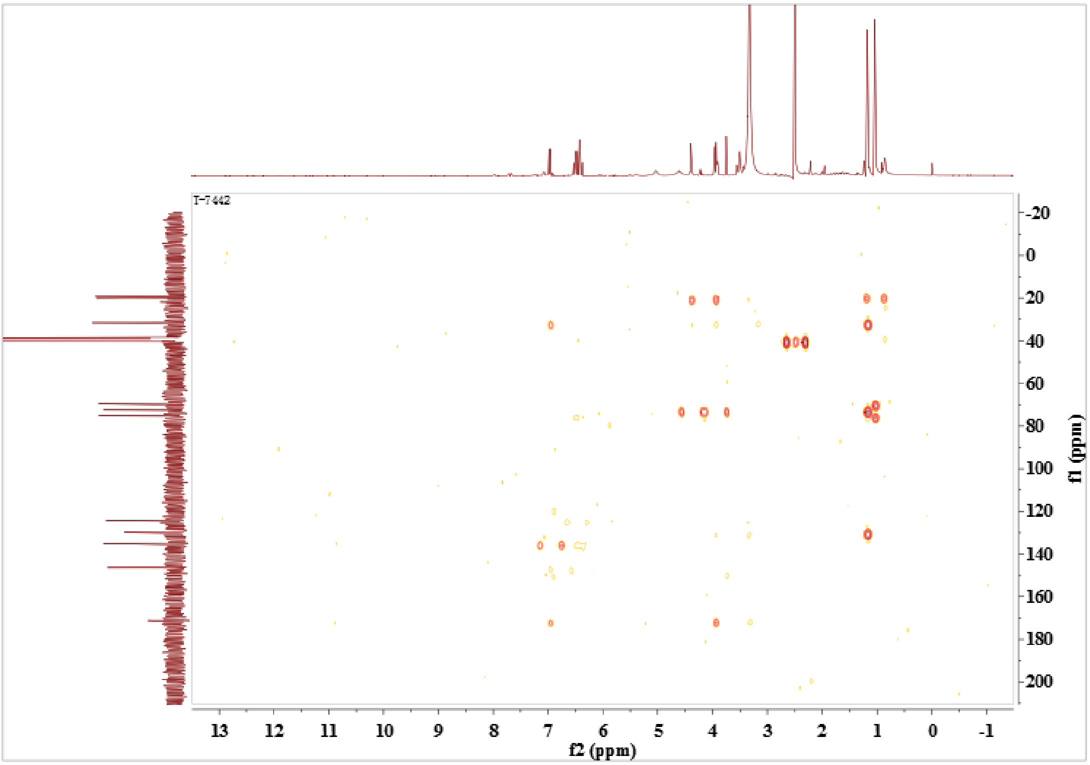

Figure S14. HMBC spectrum of **2** in DMSO-d_6

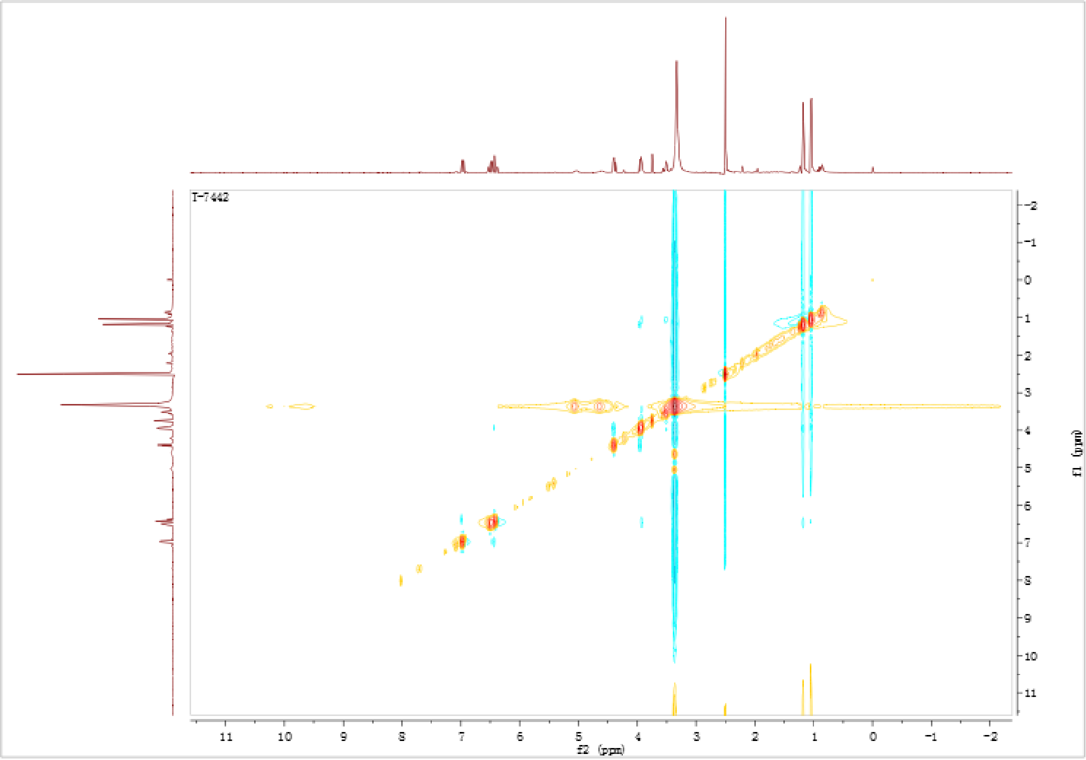

Figure S15. NOESY spectrum of **2** in DMSO-d_6

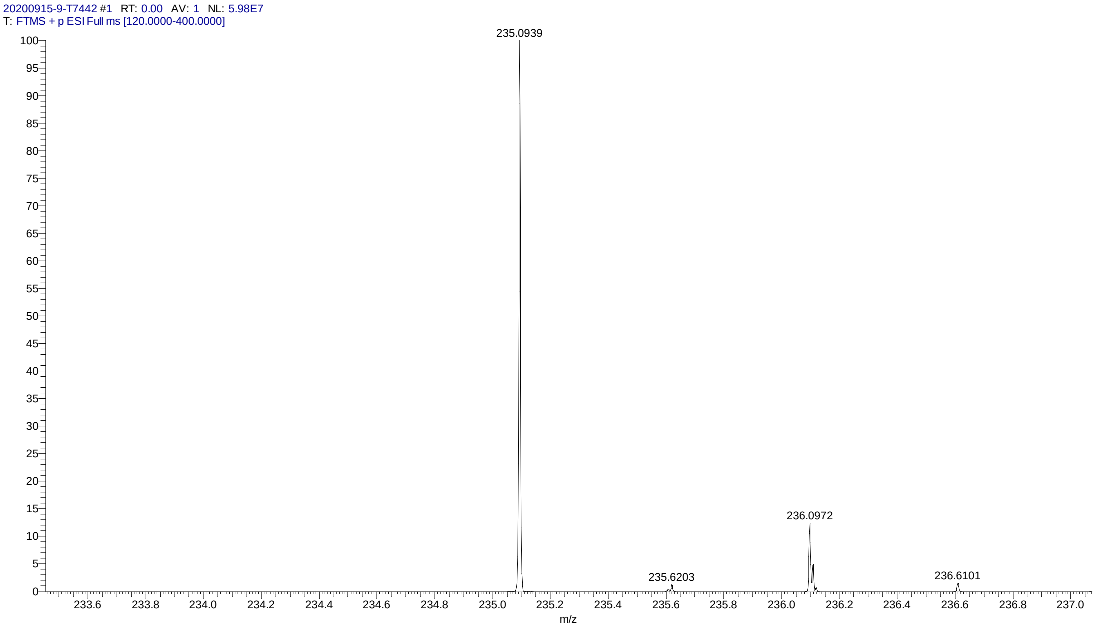

Figure S16. HR-ESI-MS spectrum of **2**

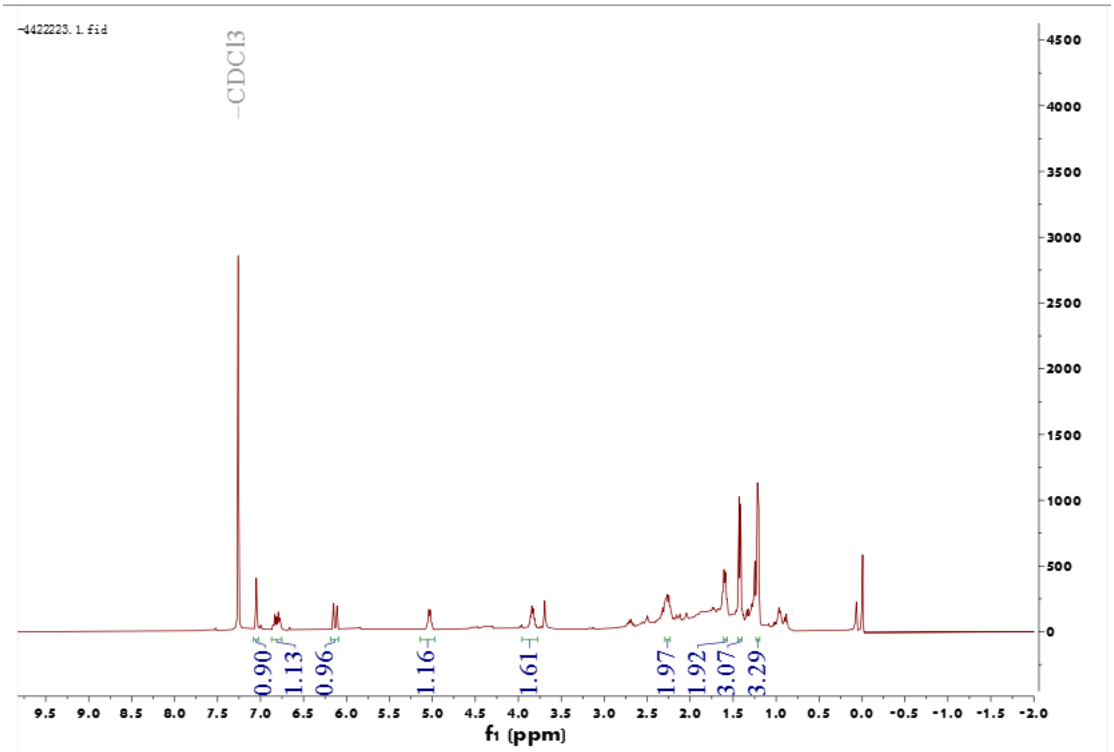

Figure S17. ^{1}H NMR spectrum of **3** in $CDCl_3$

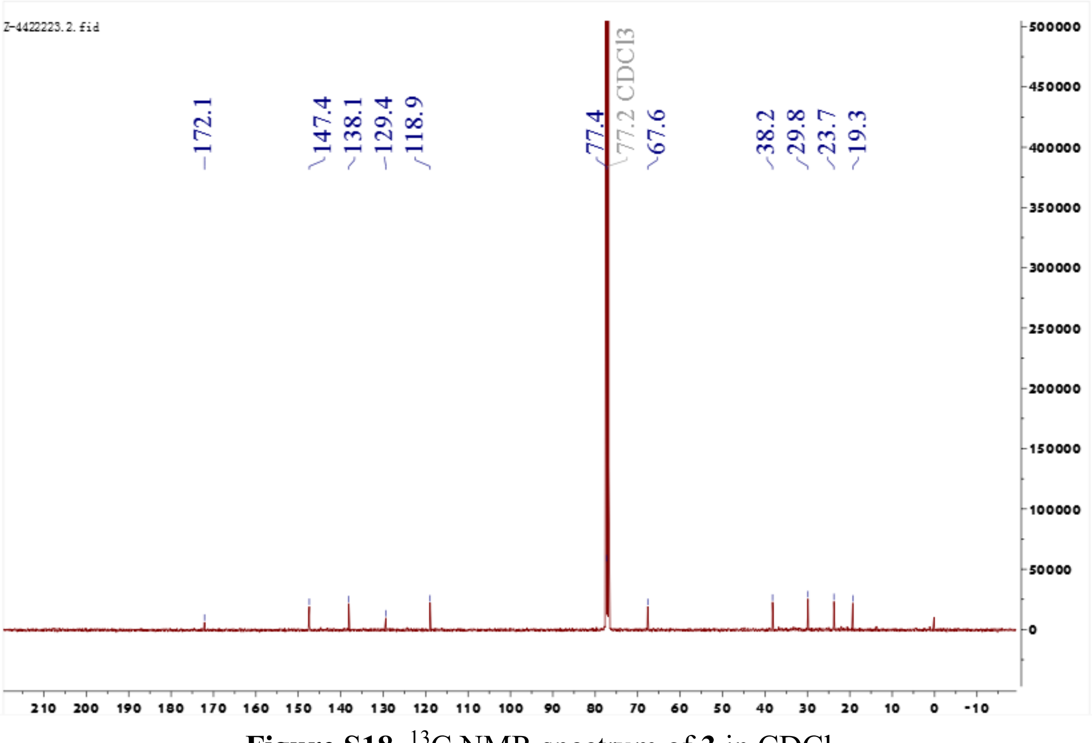

Figure S18. ^{13}C NMR spectrum of **3** in $CDCl_3$

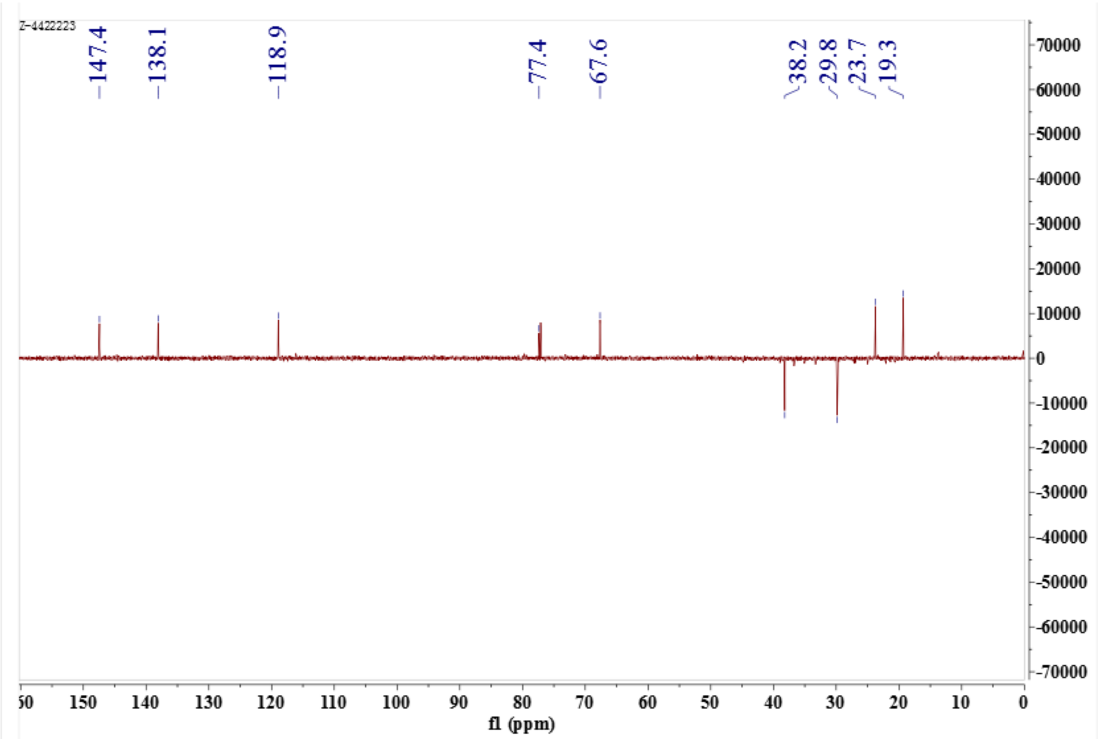

Figure S19. 135°-DEPT spectrum of **3** in $CDCl_3$

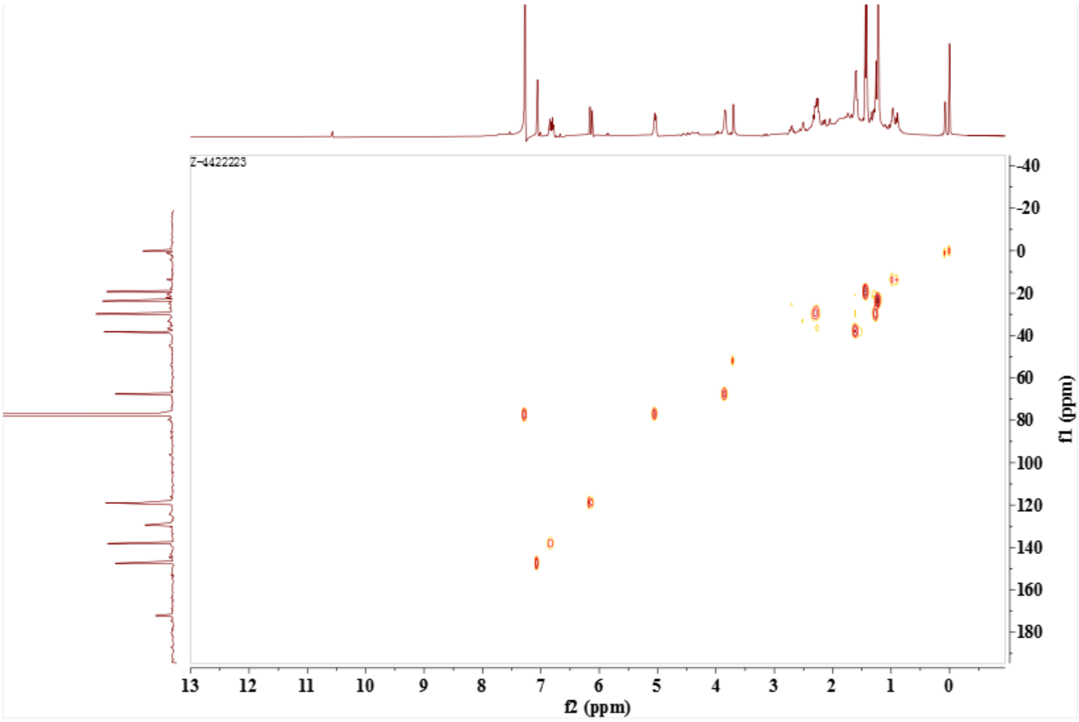

Figure S20. HMQC spectrum of **3** in $CDCl_3$

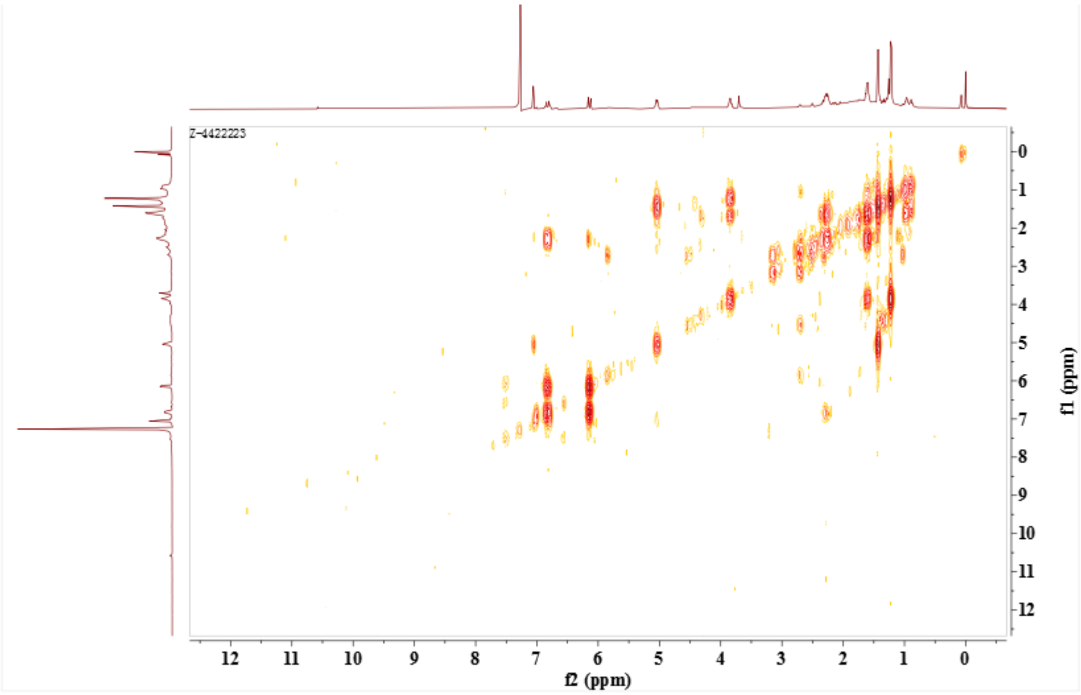

Figure S21. ^{1}H-^{1}H COSY spectrum of **3** in $CDCl_3$

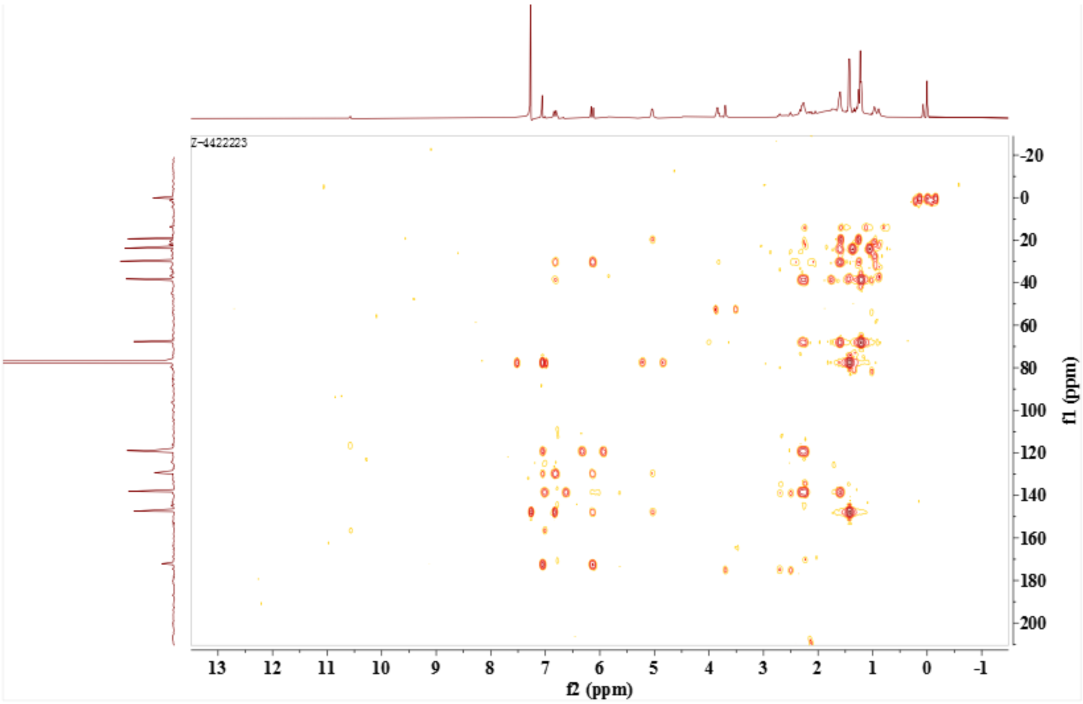

Figure S22. HMBC spectrum of **3** in $CDCl_3$

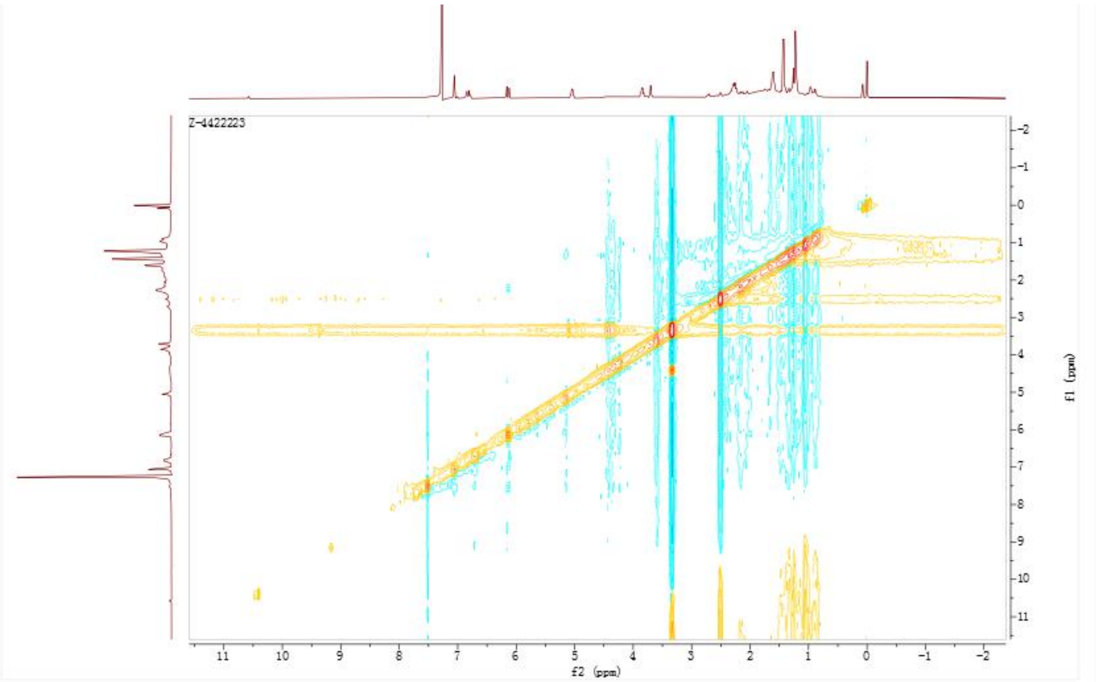

Figure S23. NOESY spectrum of **3** in $CDCl_3$

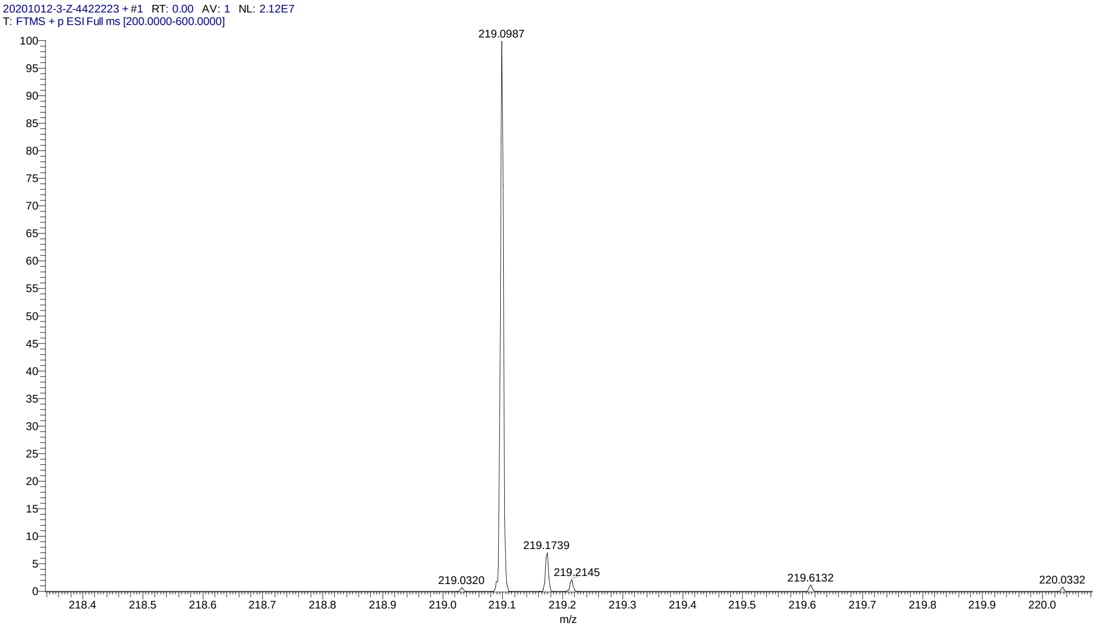

Figure S24. HR-ESI-MS spectrum of **3**

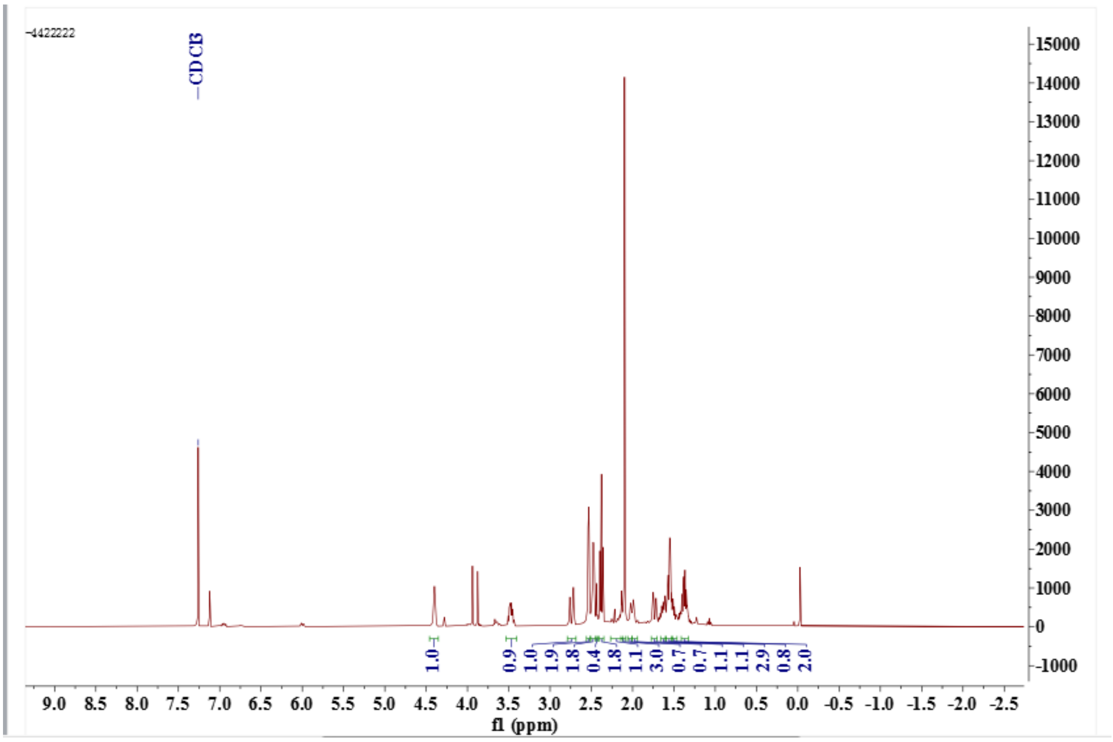

Figure S25. ^{1}H NMR spectrum of **4** in $CDCl_3$

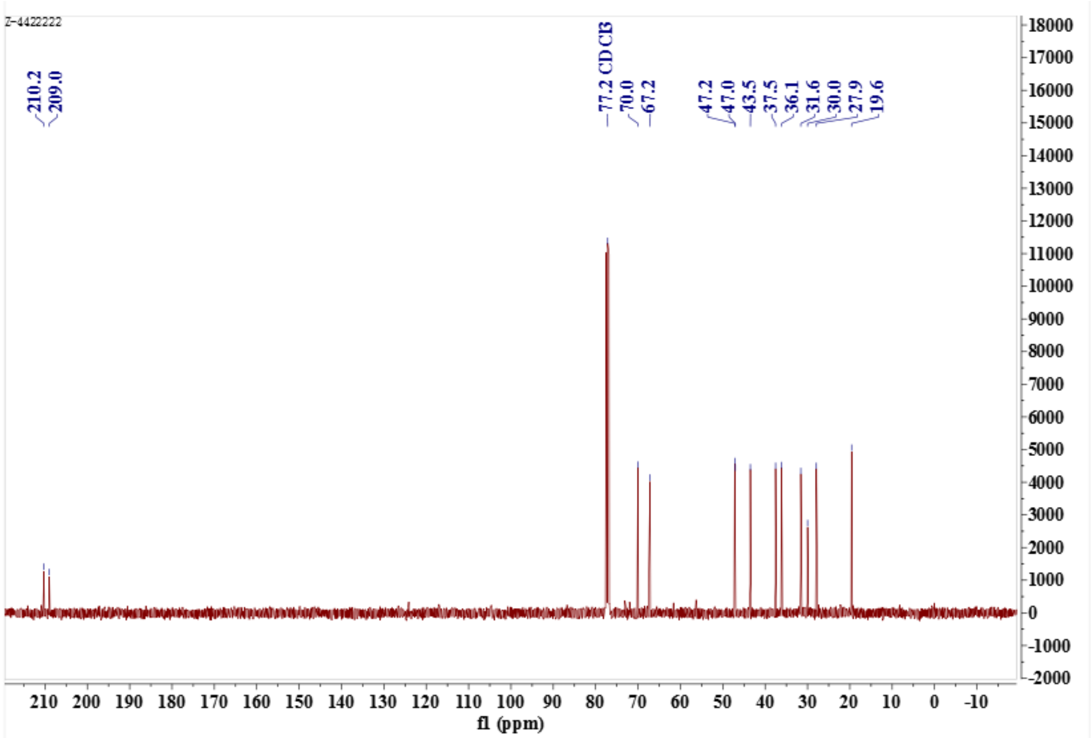

Figure S26. ^{13}C NMR spectrum of **4** in $CDCl_3$

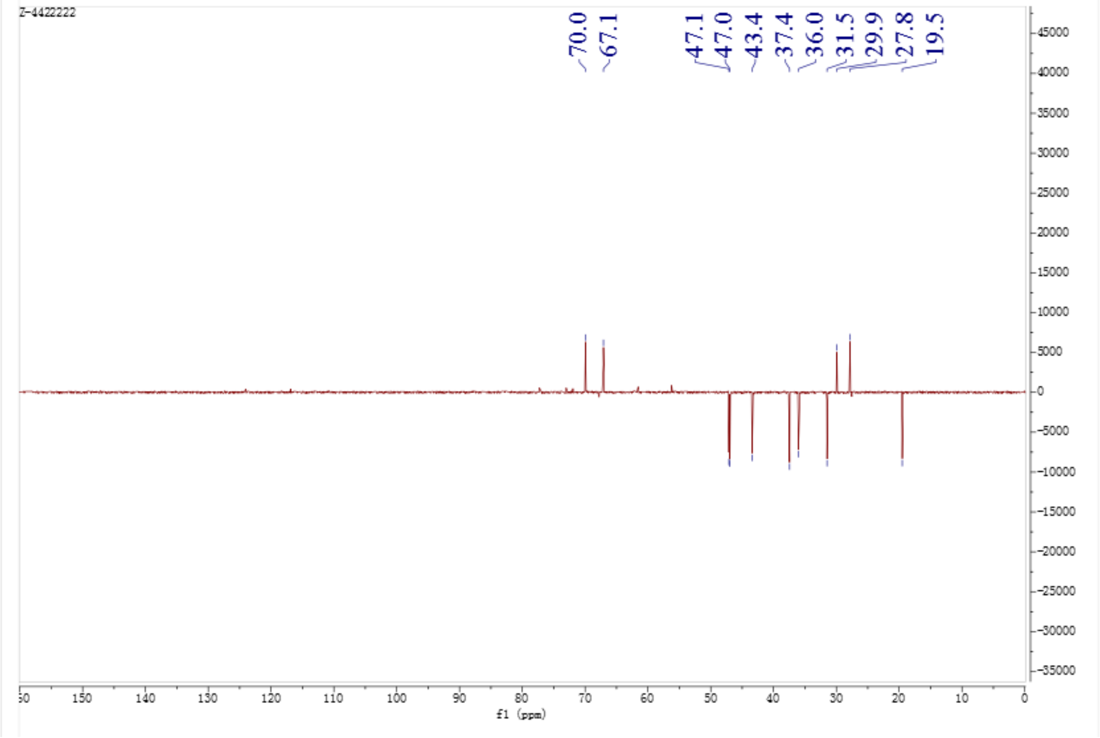

Figure S27. 135°-DEPT spectrum of **4** in $CDCl_3$

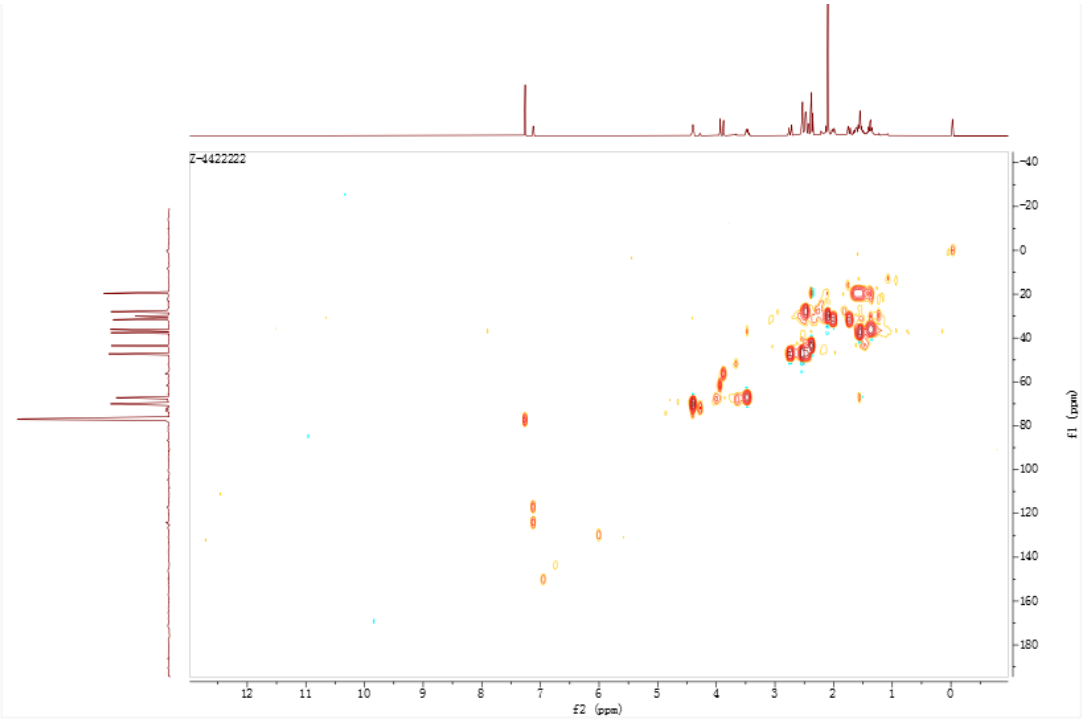

Figure S28. HMQC spectrum of **4** in $CDCl_3$

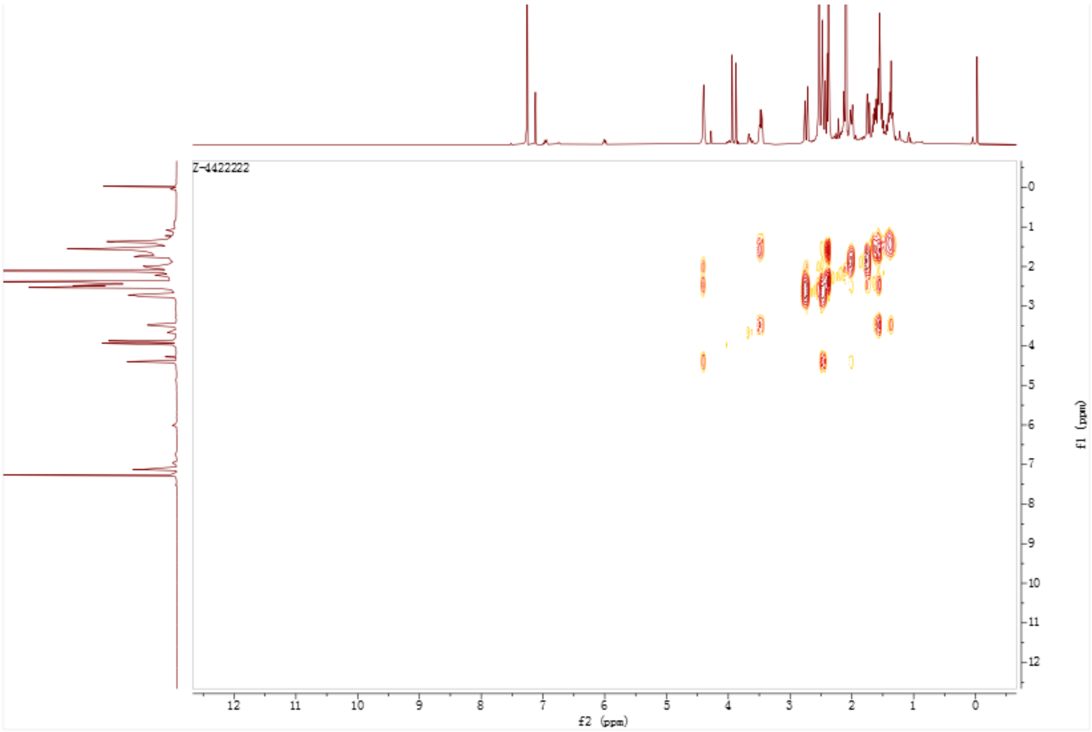

Figure S29. ^{1}H-^{1}H COSY spectrum of **4** in $CDCl_3$

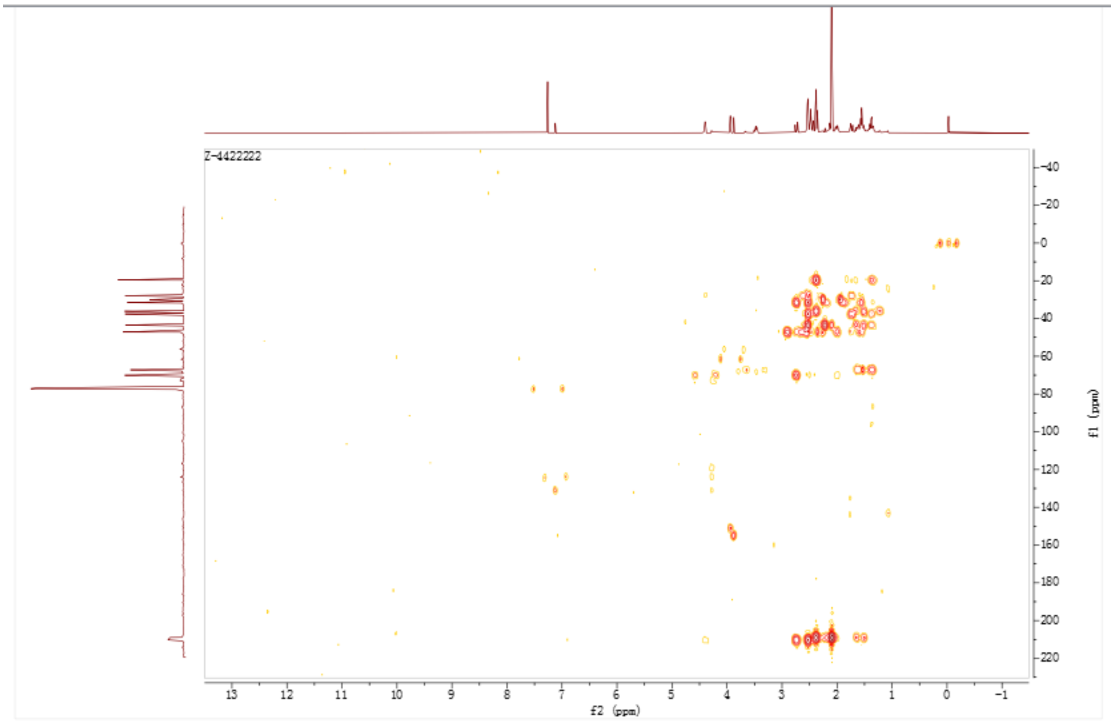

Figure S30. HMBC spectrum of **4** in $CDCl_3$

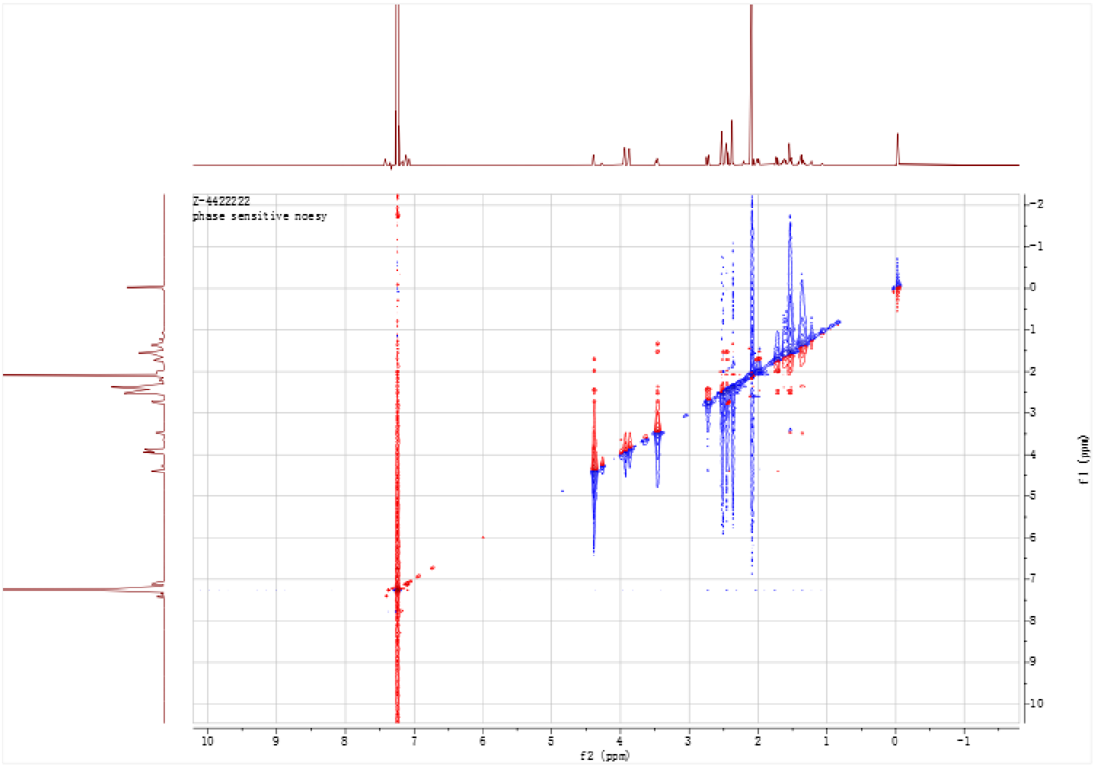

Figure S31. NOESY spectrum of **4** in $CDCl_3$

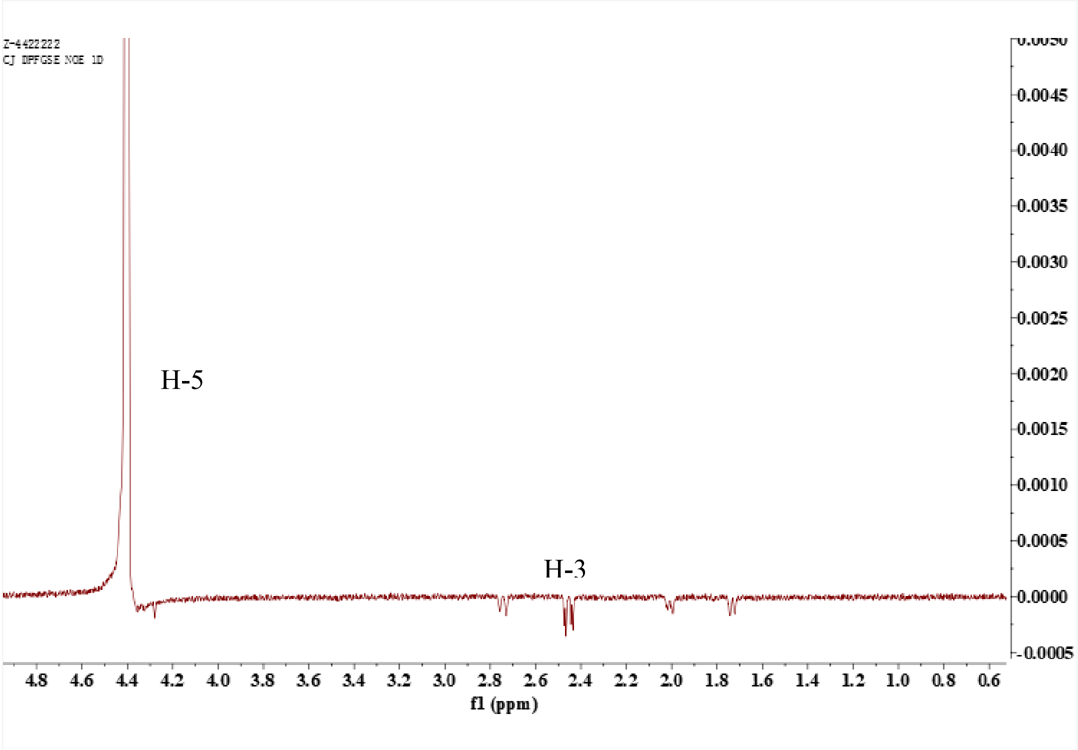

Figure S32. 1D NOE spectrum of **4** in $CDCl_3$

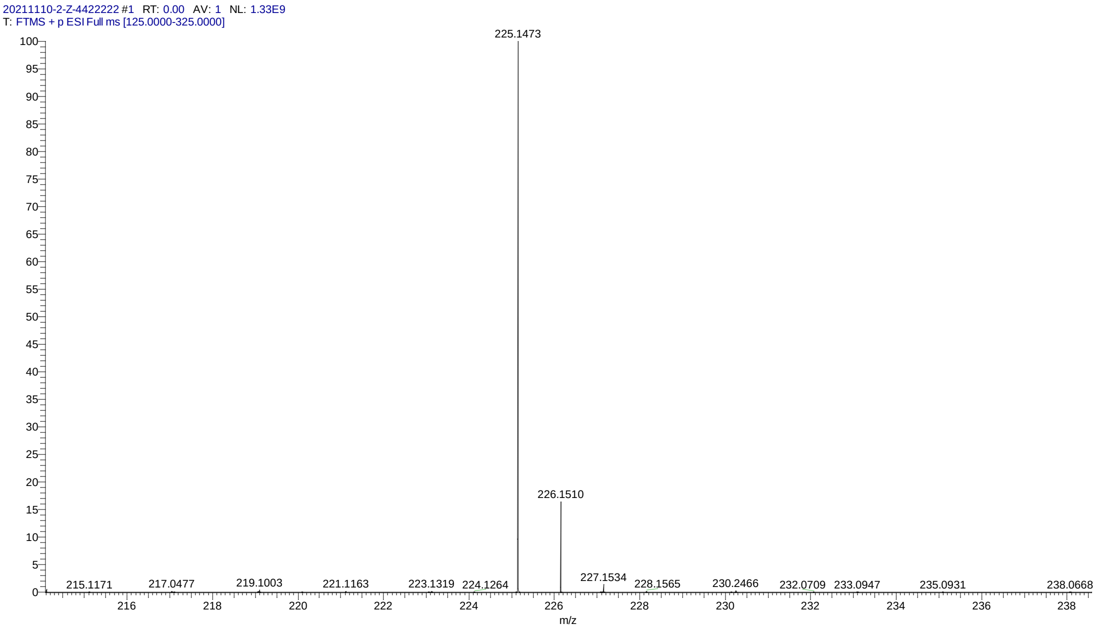

Figure S33. HR-ESI-MS spectrum of **4**

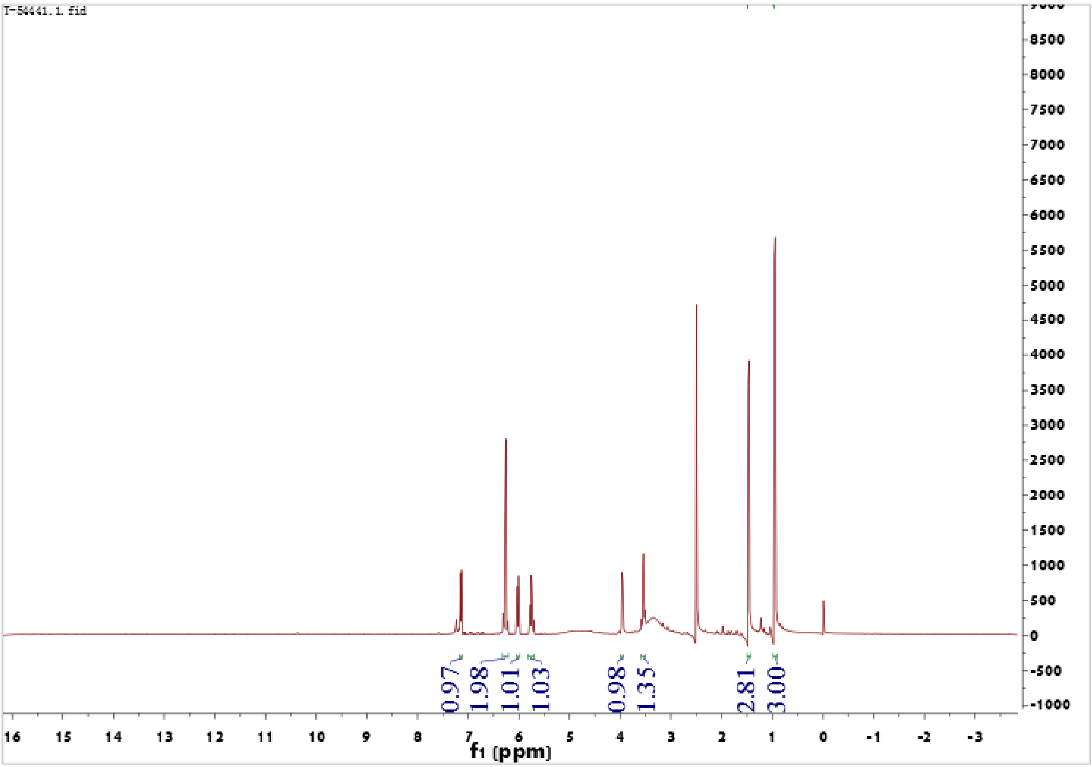

Figure S34. ^{1}H NMR spectrum of **5** in DMSO-d_6

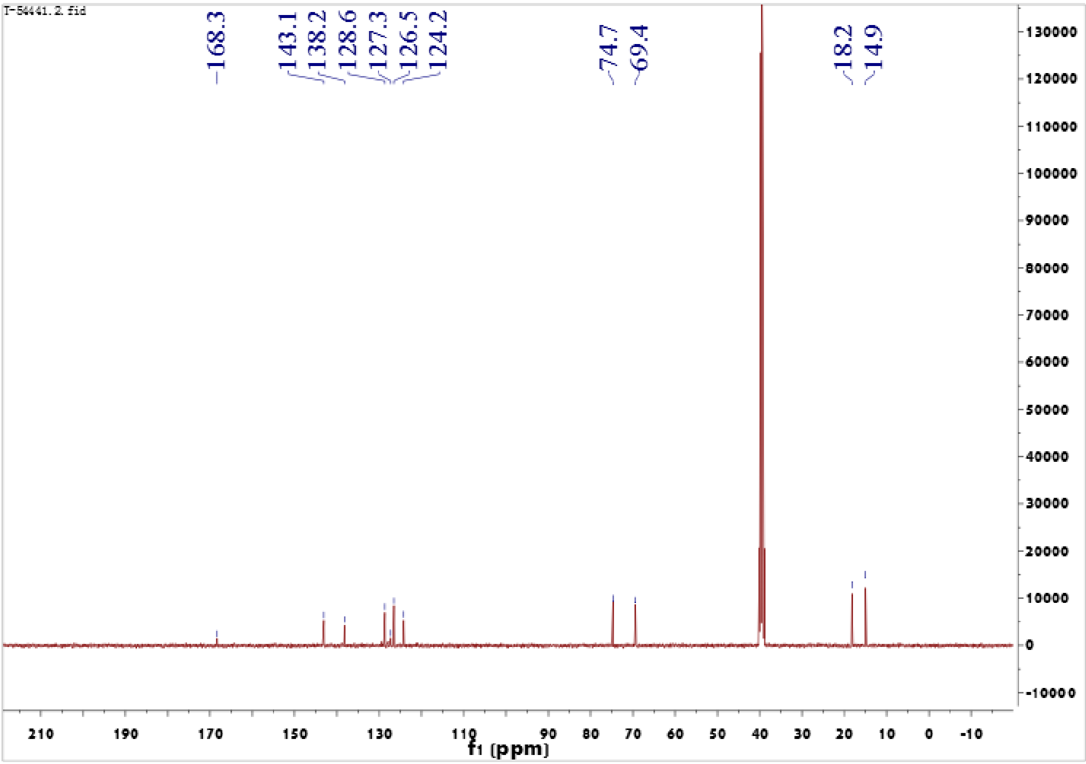

Figure S35. ^{13}C NMR spectrum of **5** in DMSO-d_6

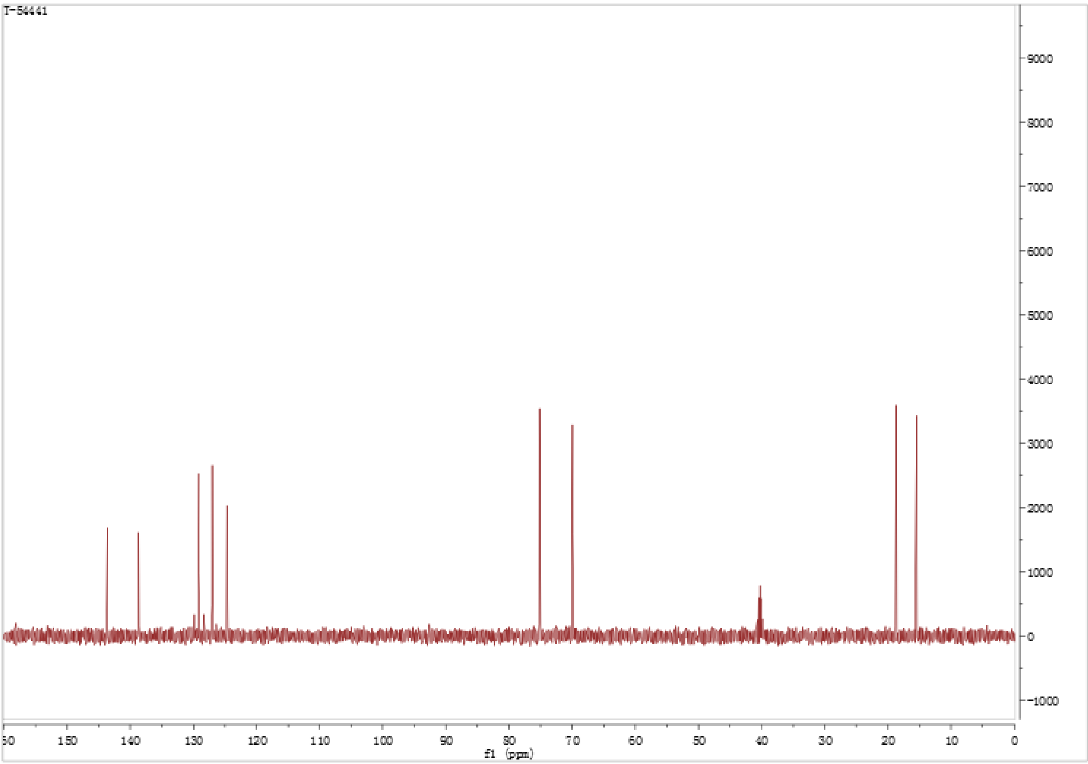

Figure S36. 135°-DEPT spectrum of **5** in DMSO-d_6

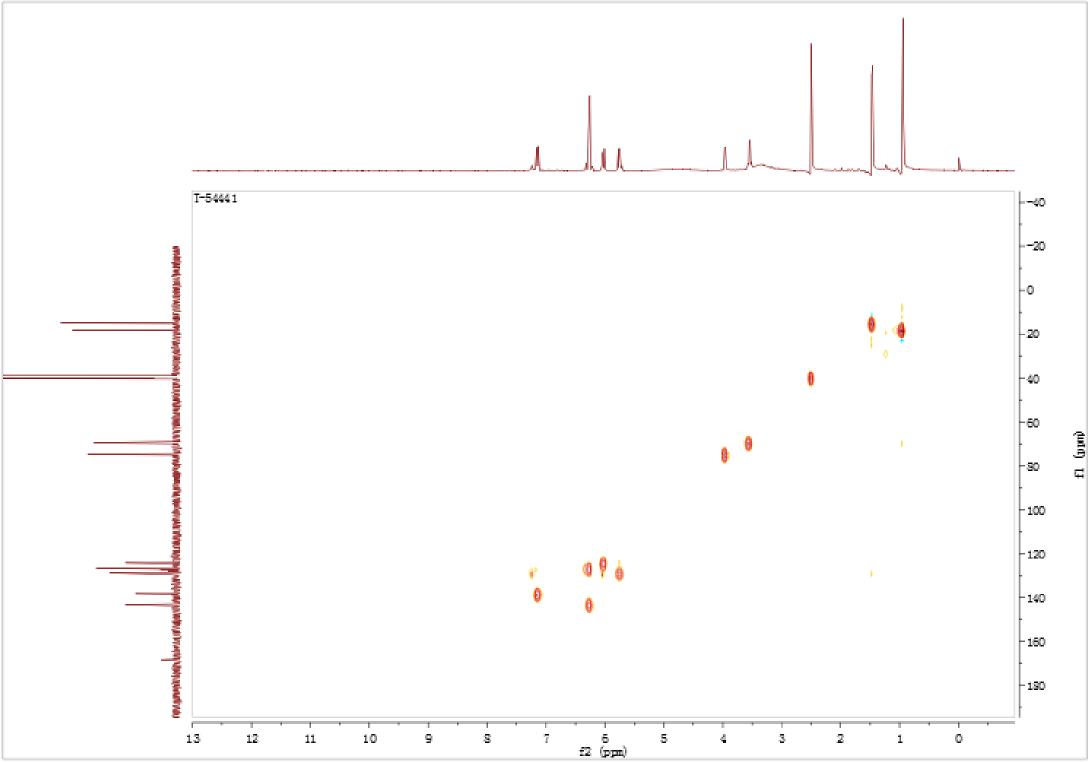

Figure S37. HMQC spectrum of **5** in DMSO-d_6

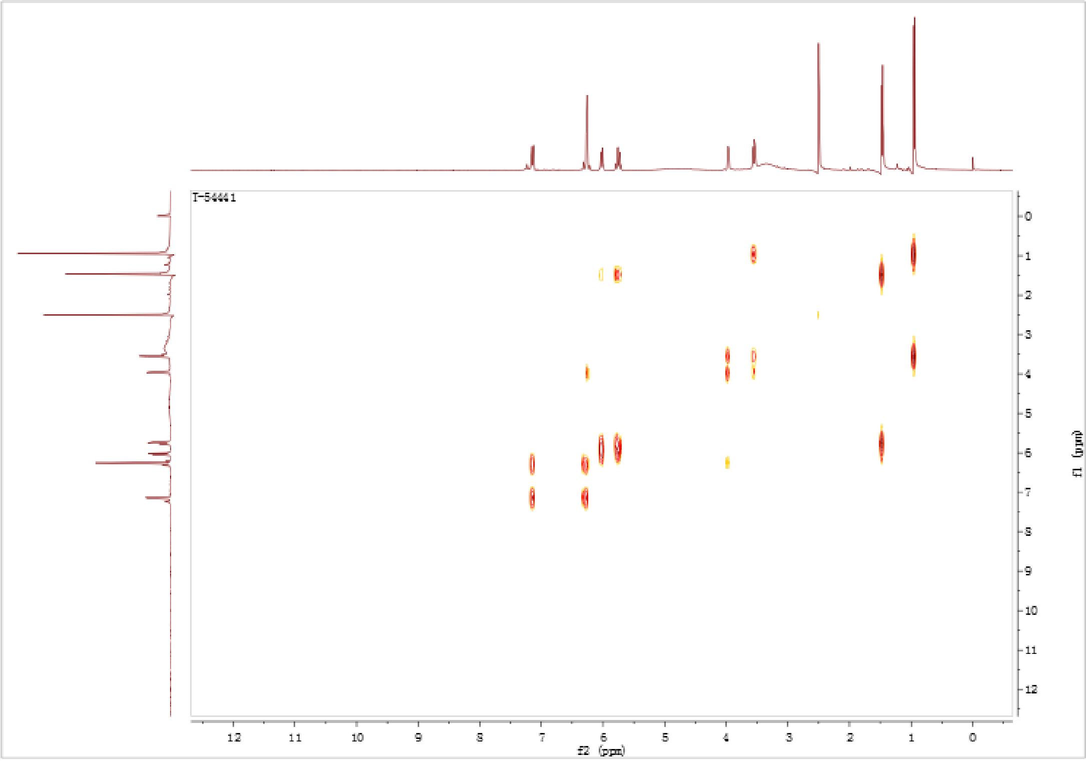

Figure S38. ^{1}H-^{1}H COSY spectrum of **5** in DMSO-d_6

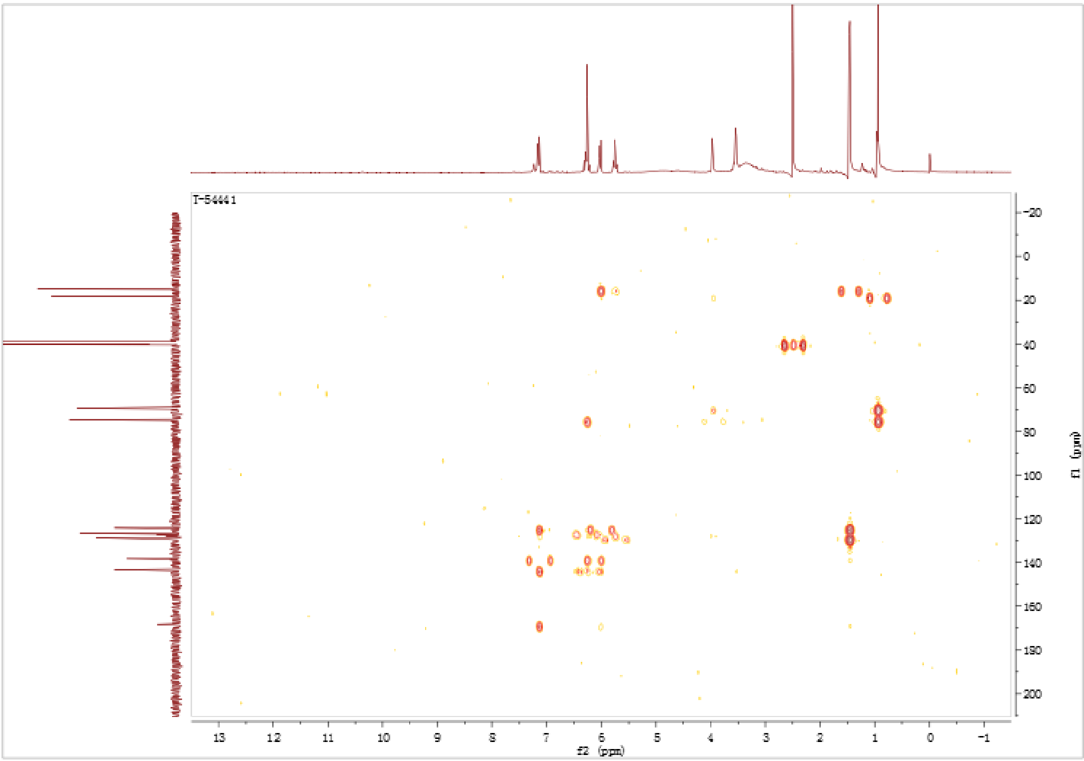

Figure S39. HMBC spectrum of **5** in DMSO-d_6

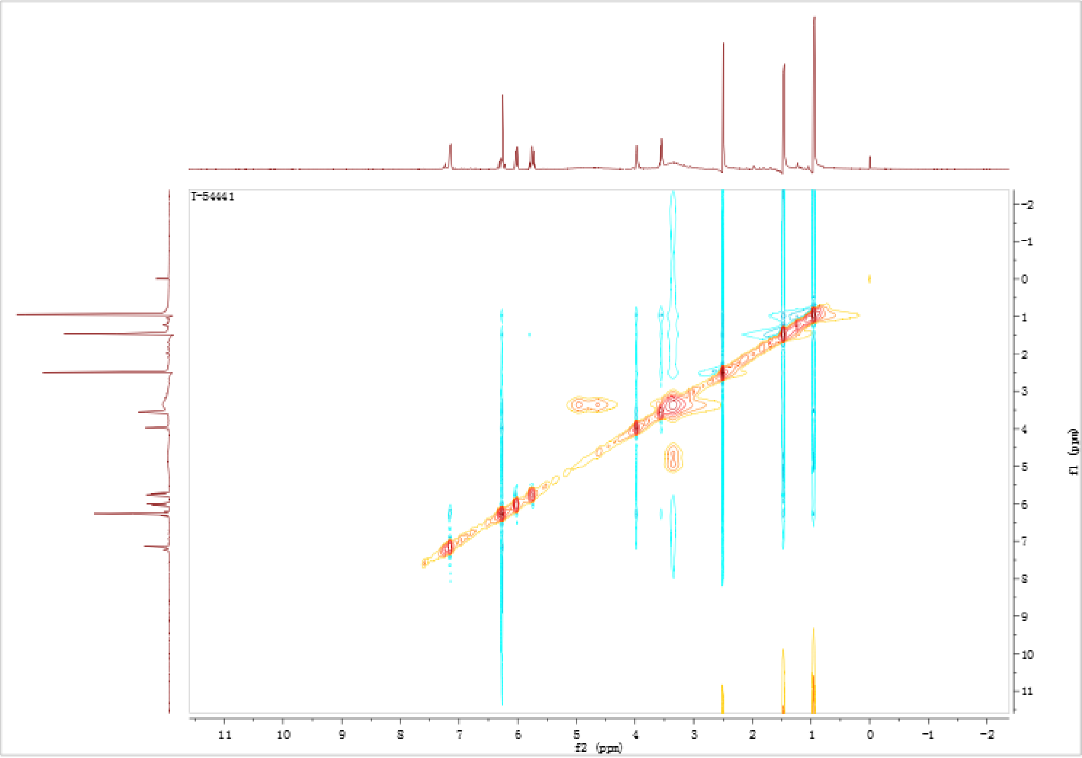

Figure S40. NOESY spectrum of **5** in DMSO-d_6

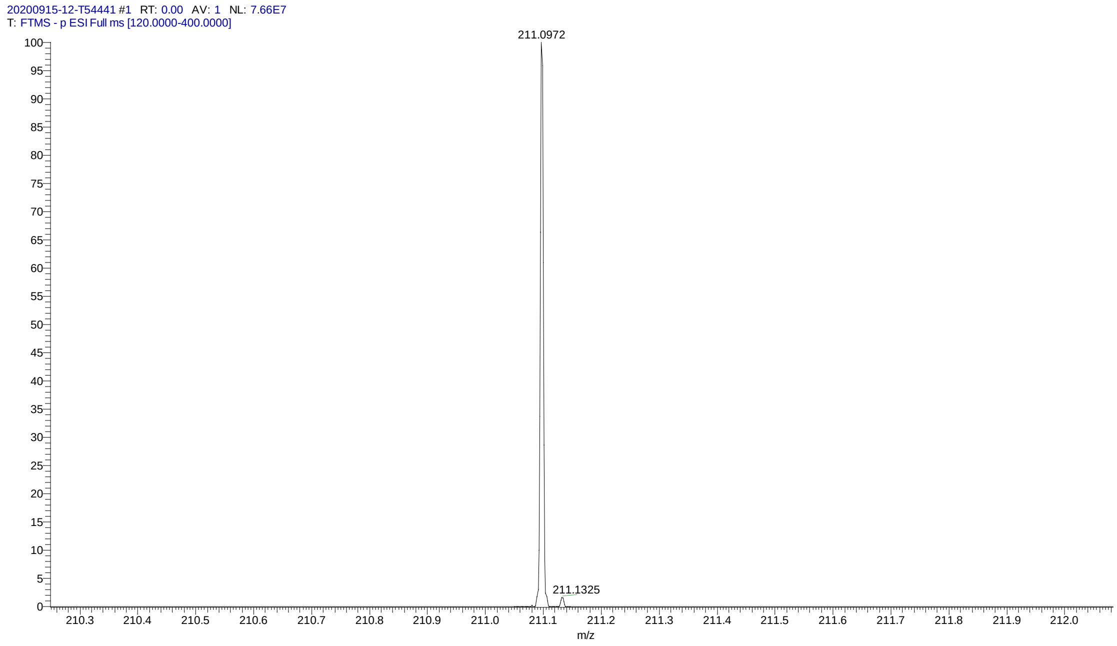

Figure S41. HR-ESI-MS spectrum of **5**

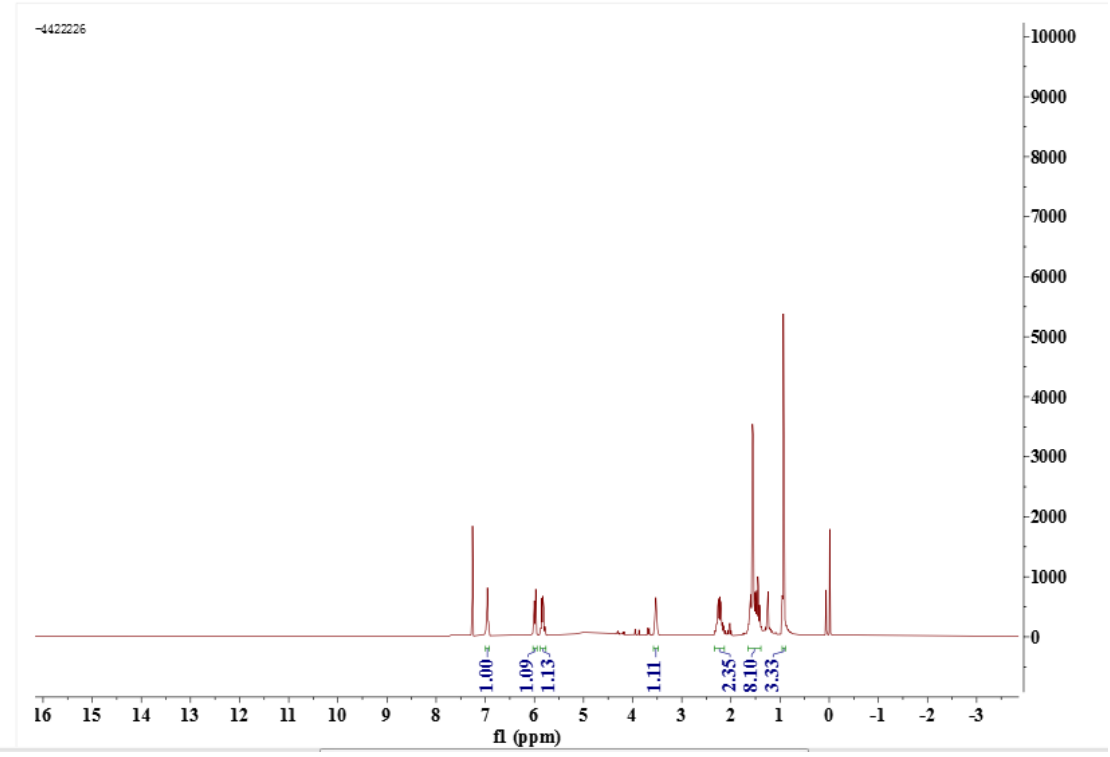

Figure S42. ^{1}H NMR spectrum of **6** in $CDCl_3$

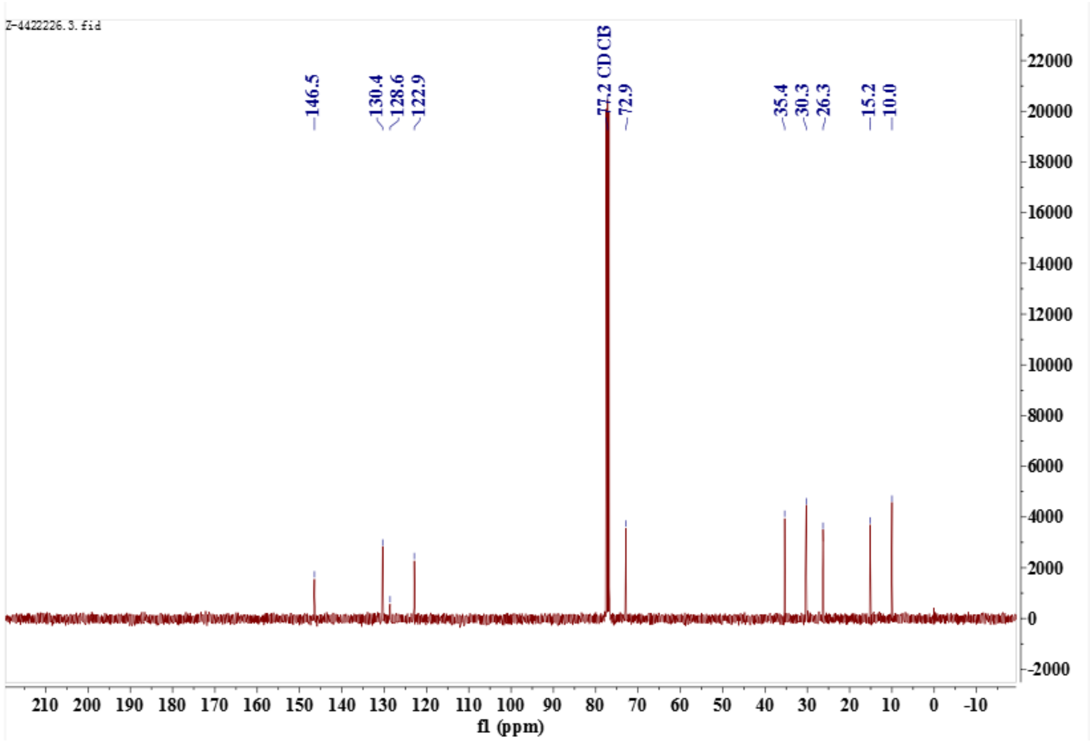

Figure S43. ^{13}C NMR spectrum of **6** in $CDCl_3$

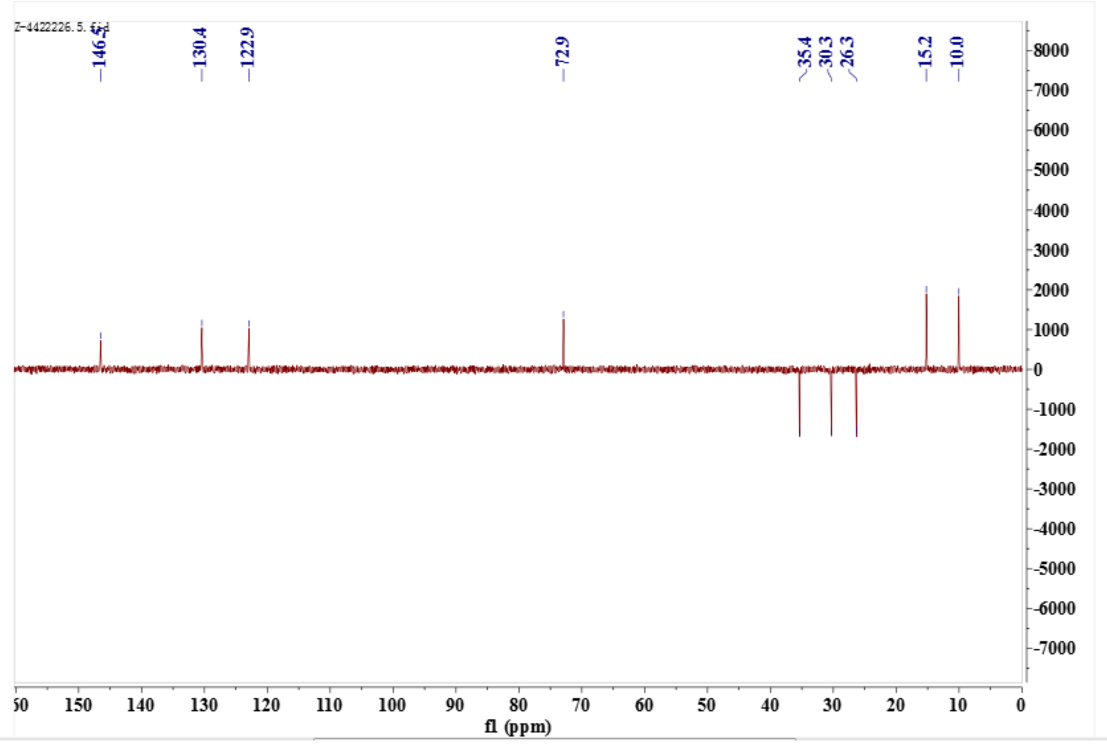

Figure S44. 135°-DEPT spectrum of **6** in $CDCl_3$

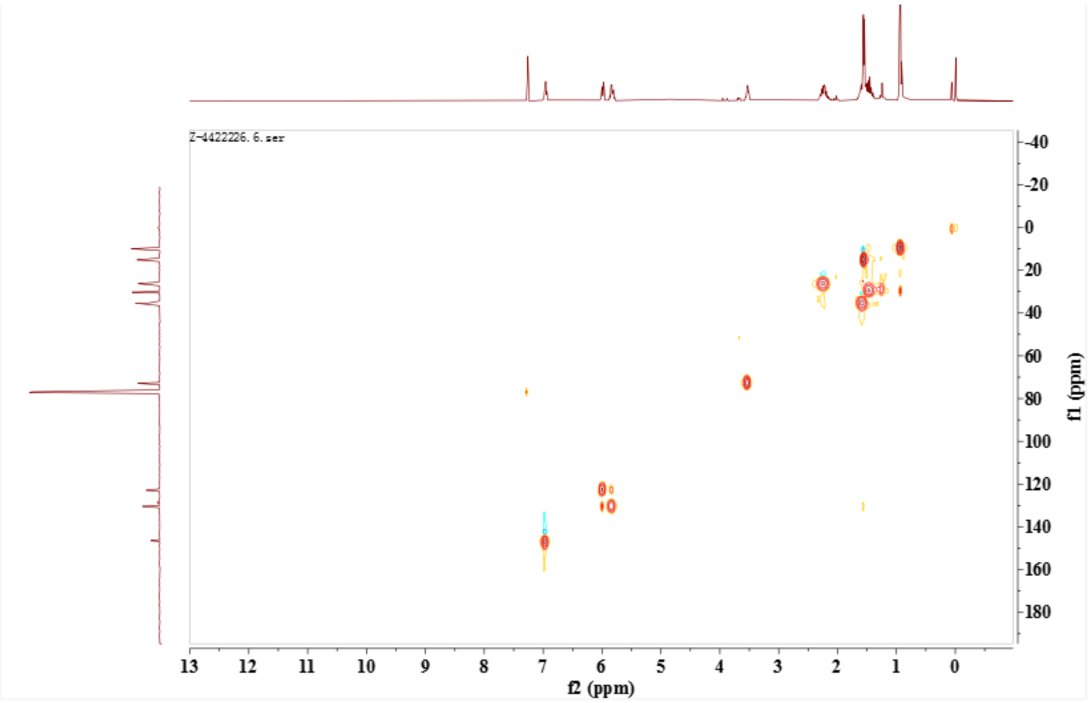

Figure S45. HMQC spectrum of **6** in $CDCl_3$

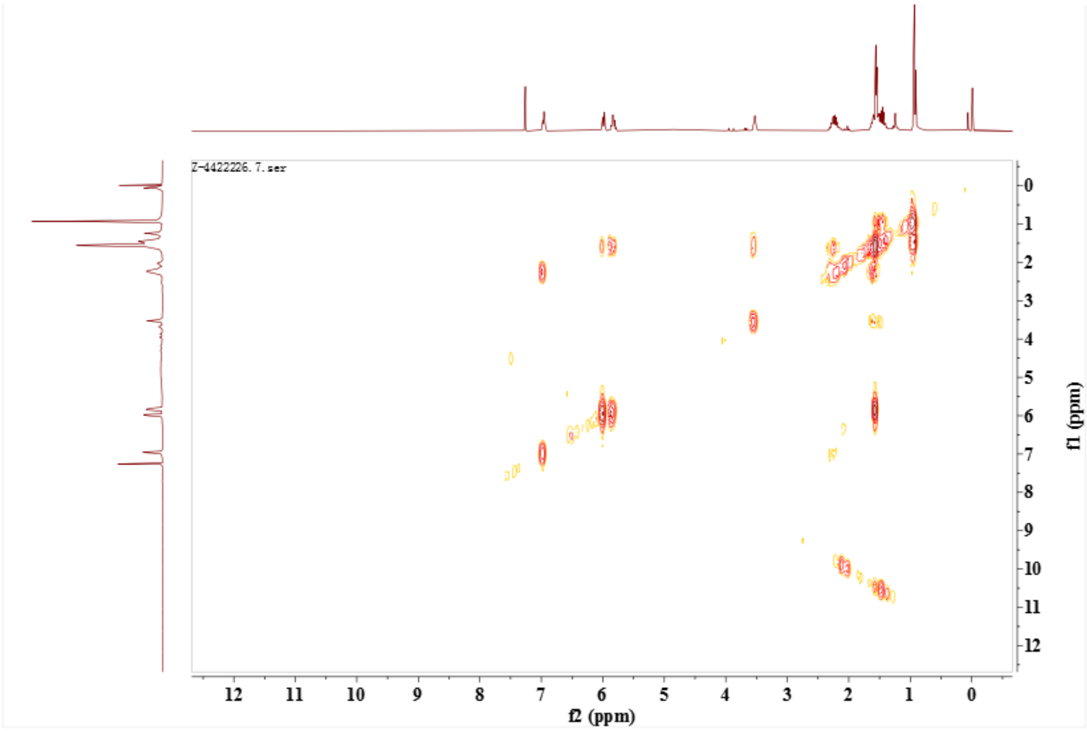

Figure S46. ^1H-^1H COSY spectrum of **6** in $CDCl_3$

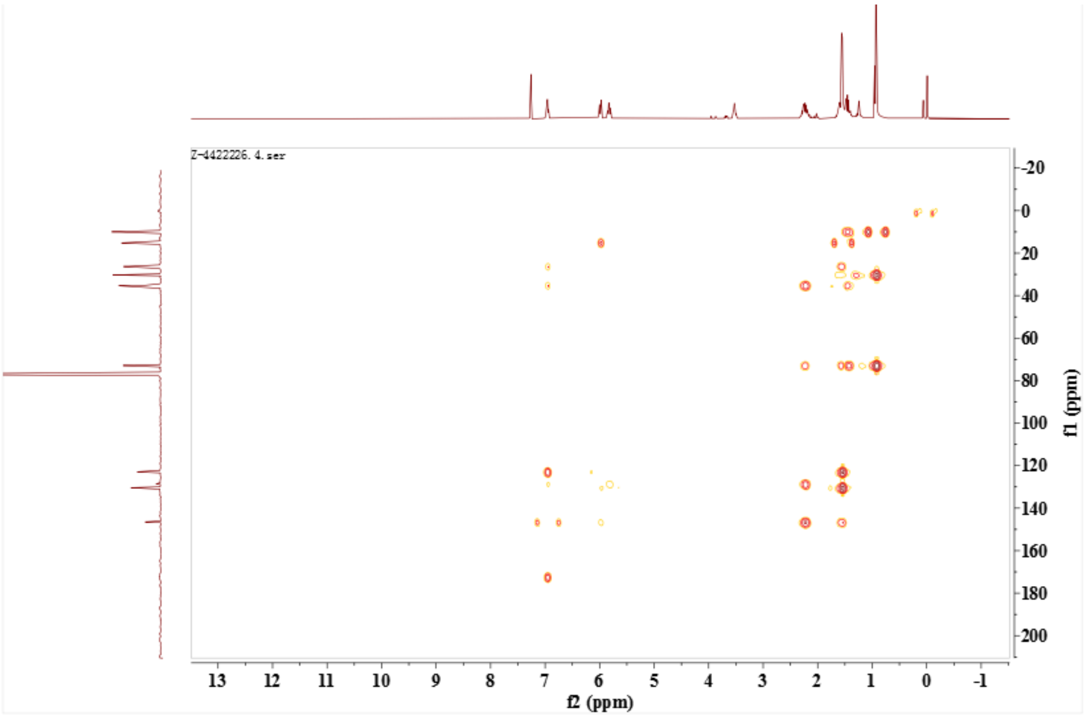

Figure S47. HMBC spectrum of **6** in $CDCl_3$

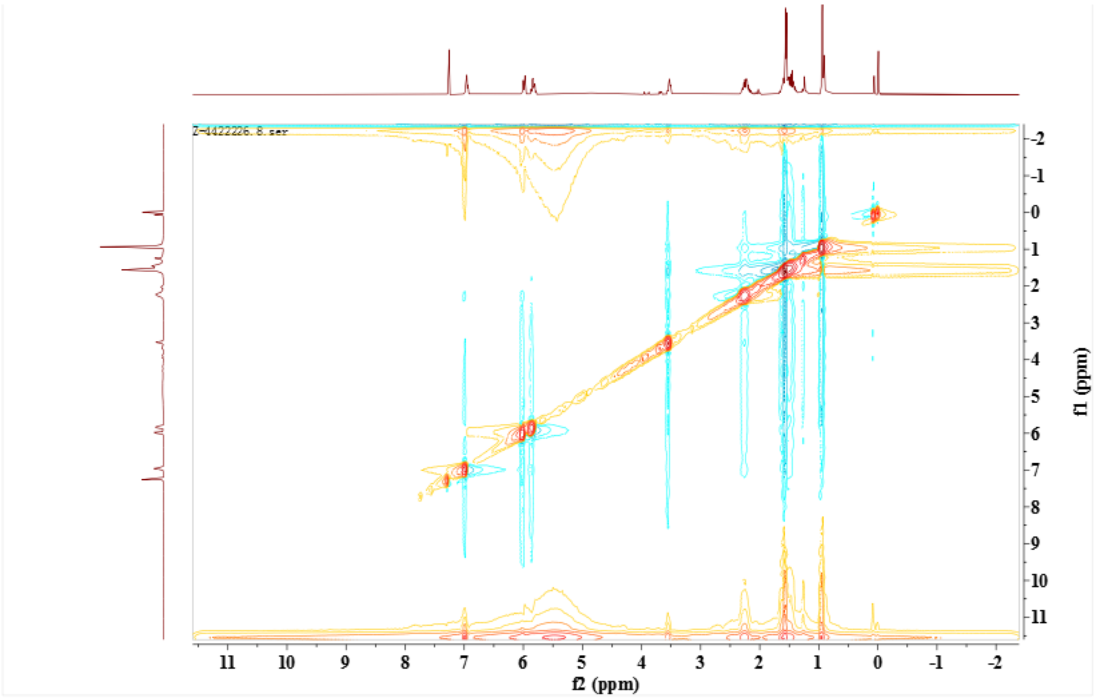

Figure S48. NOESY spectrum of **6** in $CDCl_3$

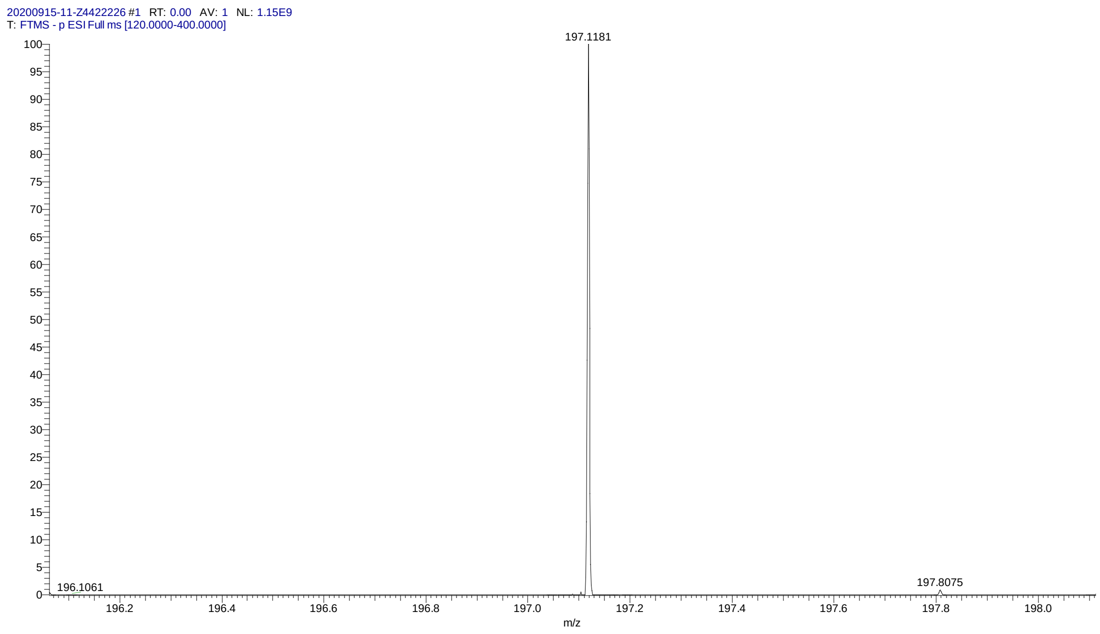

Figure S49. HR-ESI-MS spectrum of **6**

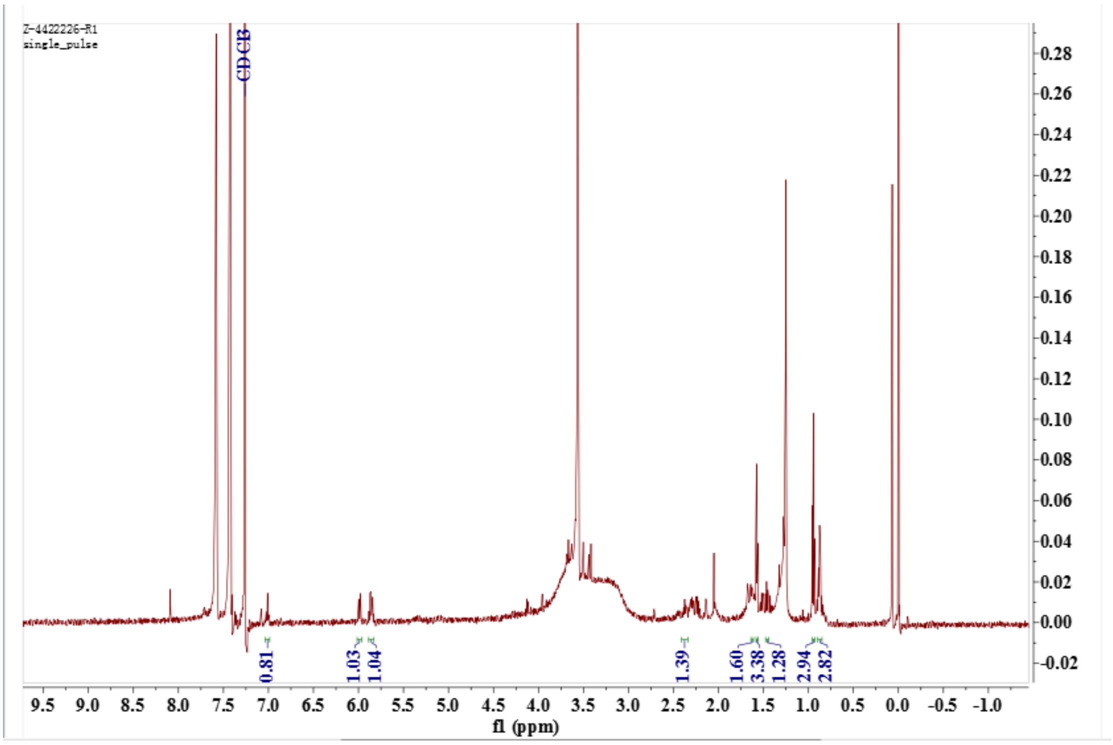

Figure S50. ^{1}H NMR ($CDCl_3$, 600 MHz) of *R*-MTPA ester of **6**

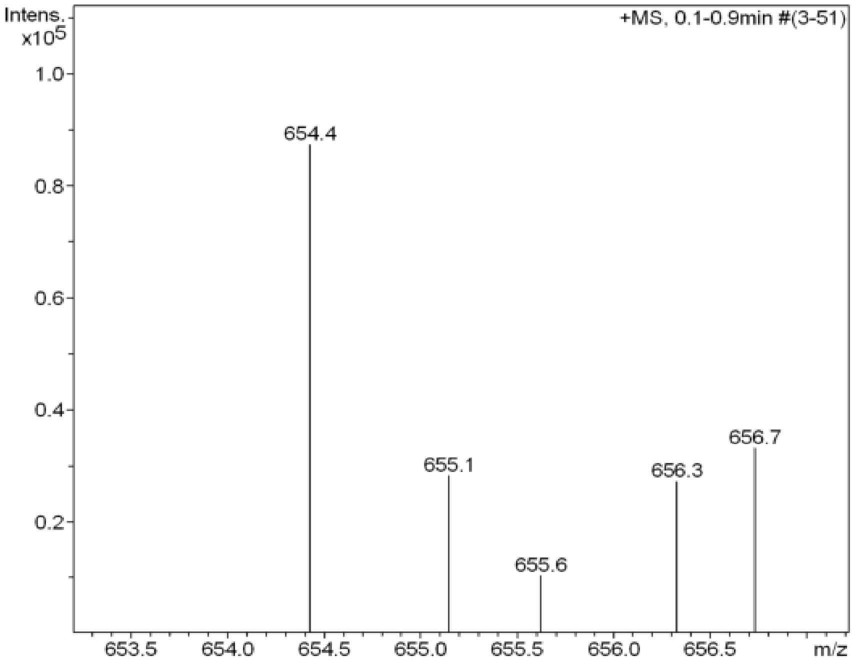

Figure S51. ESI-MS spectrum of *R*-MTPA ester of **6**

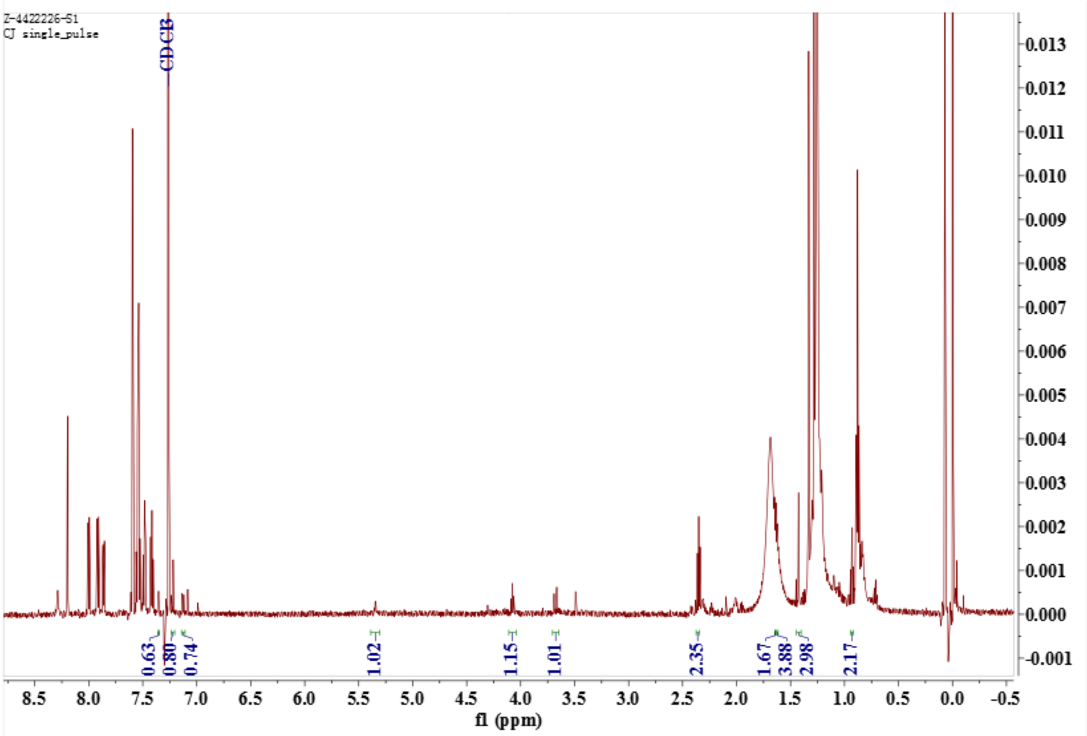

Figure S52. ^{1}H NMR ($CDCl_3$, 600 MHz) of *S*-MTPA ester of **6**

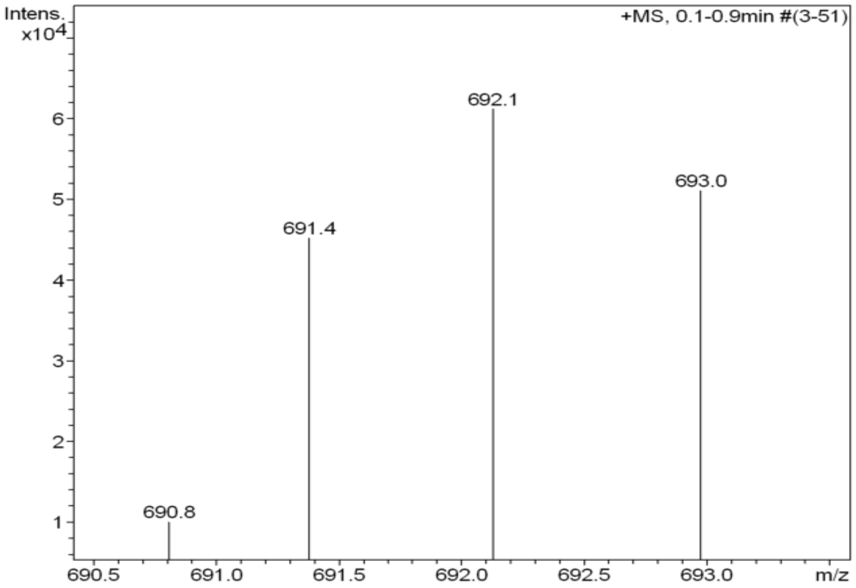

Figure S53. ESI-MS spectrum of *S*-MTPA ester of **6**

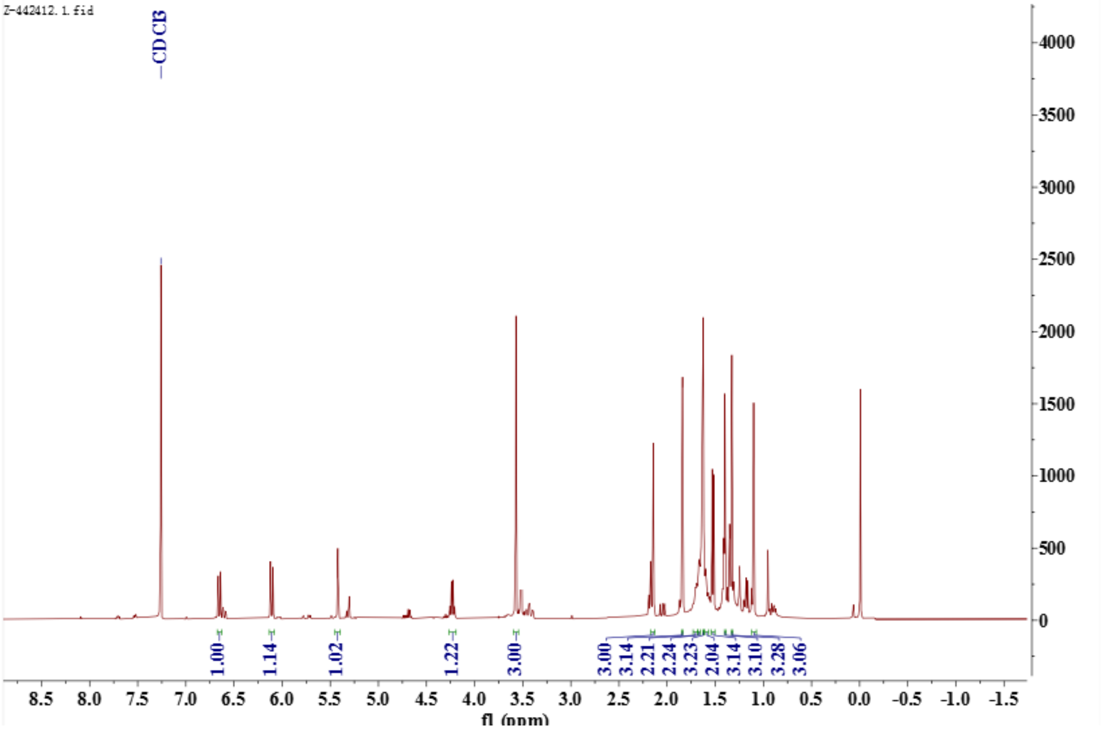

Figure S54. ^{1}H NMR spectrum of **7** in $CDCl_3$

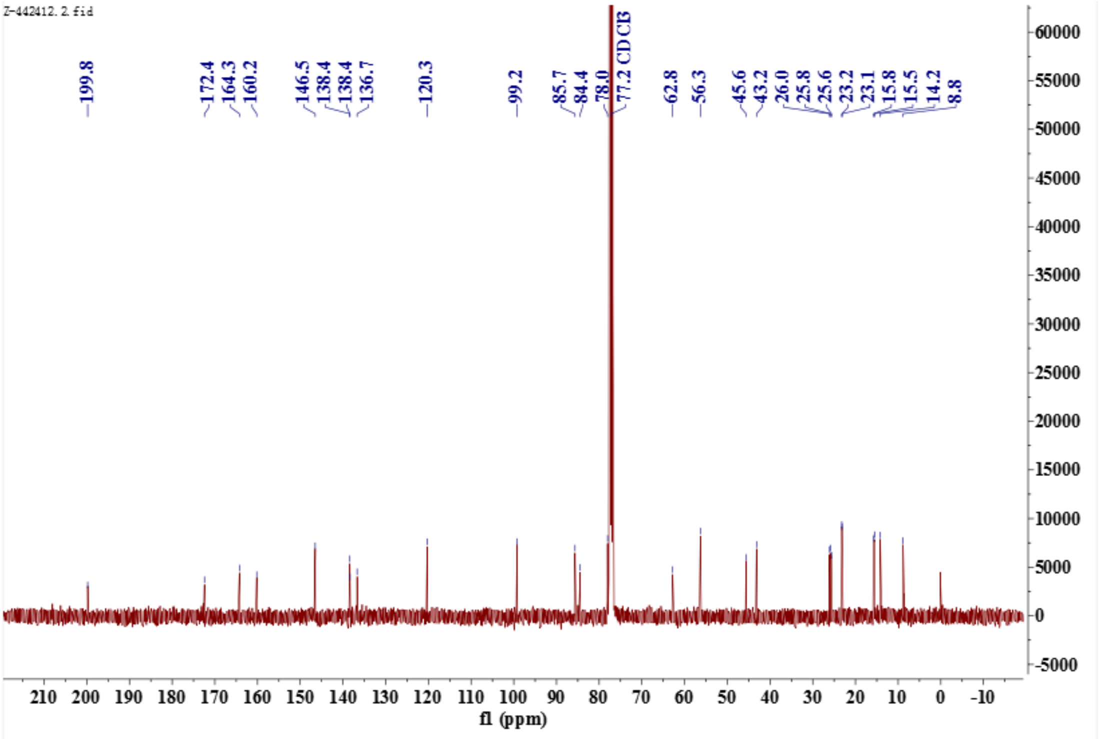

Figure S55. ^{13}C NMR spectrum of **7** in $CDCl_3$

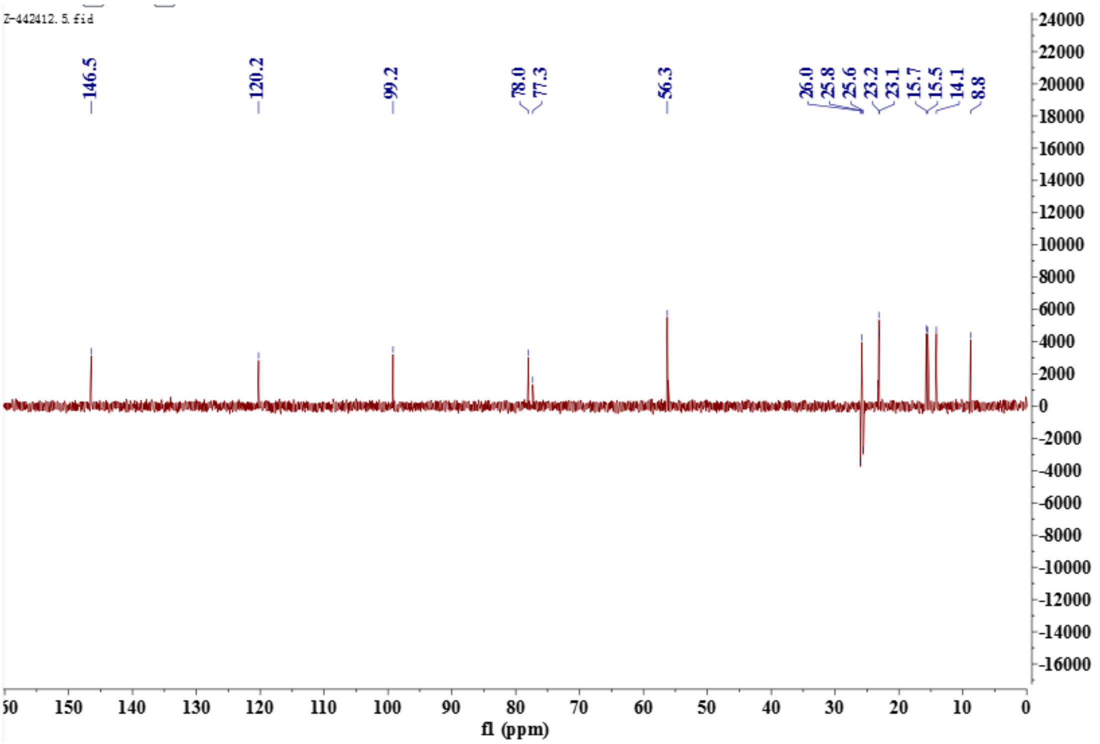

Figure S56. 135°-DEPT spectrum of **7** in $CDCl_3$

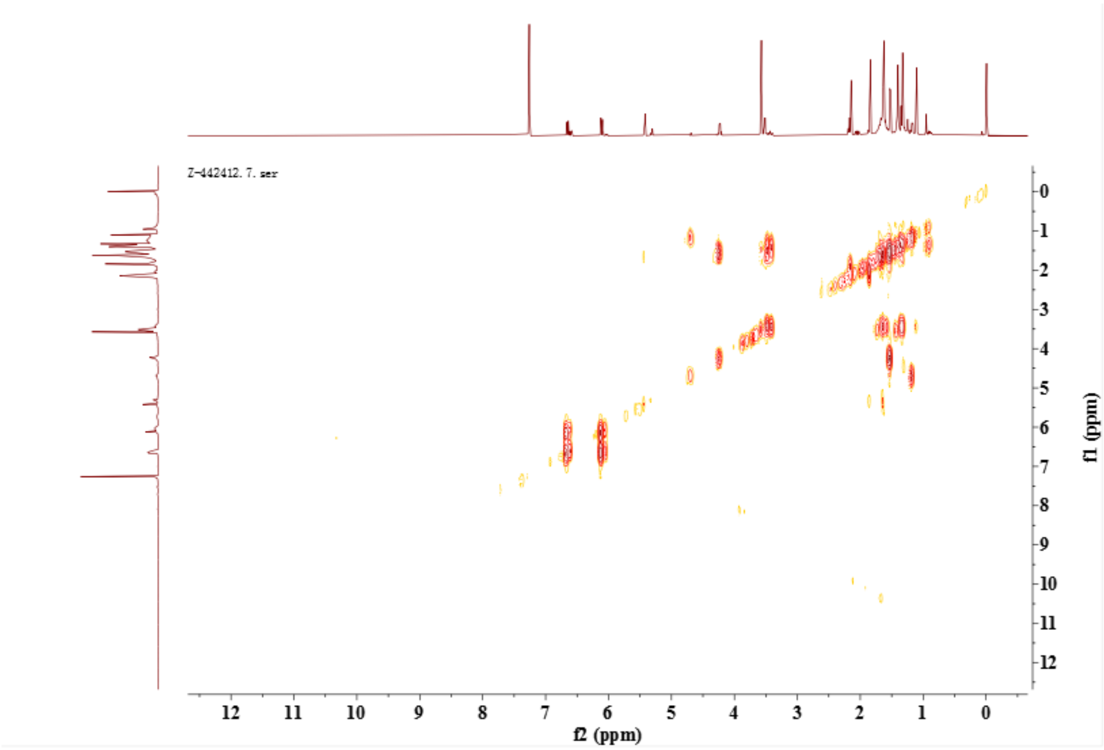

Figure S57. HMQC spectrum of **7** in $CDCl_3$

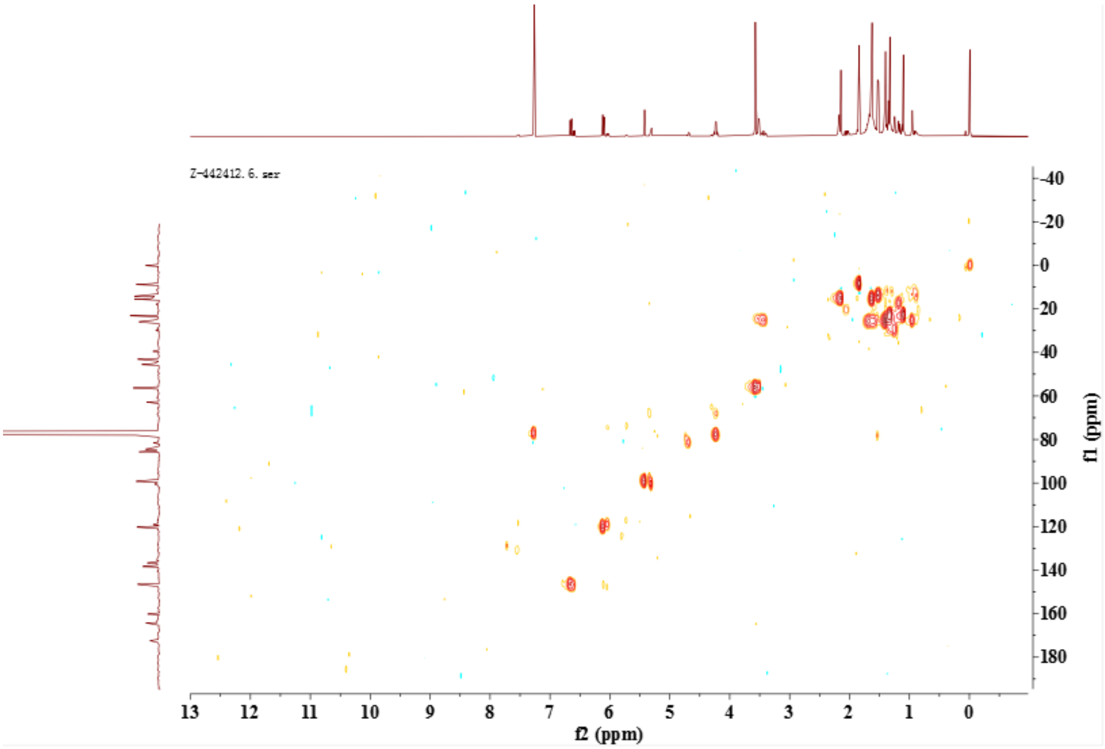

Figure S58. ^1H-^1H COSY spectrum of **7** in $CDCl_3$

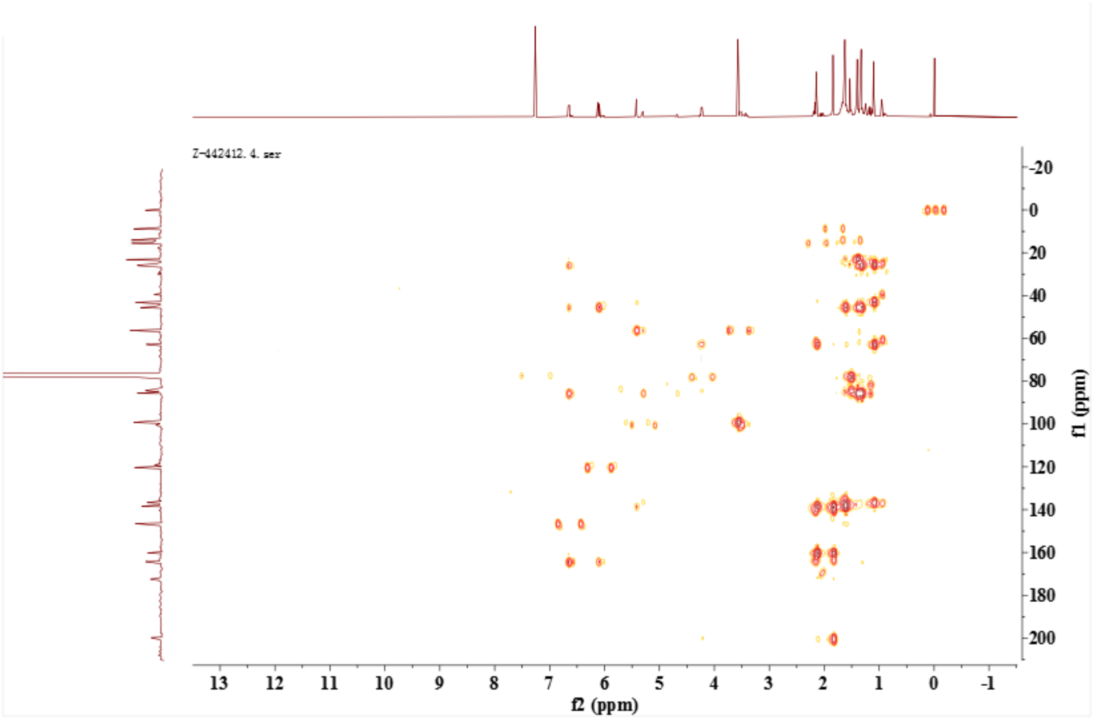

Figure S59. HMBC spectrum of **7** in $CDCl_3$

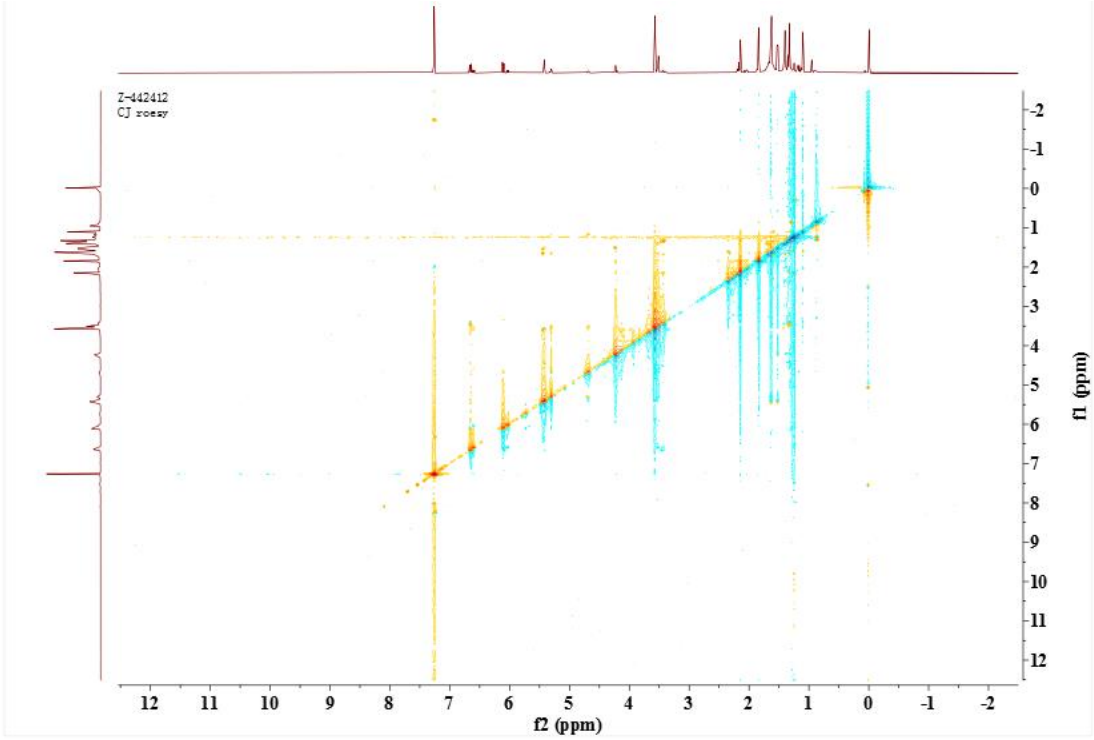

Figure S60. ROESY spectrum of **7** in $CDCl_3$

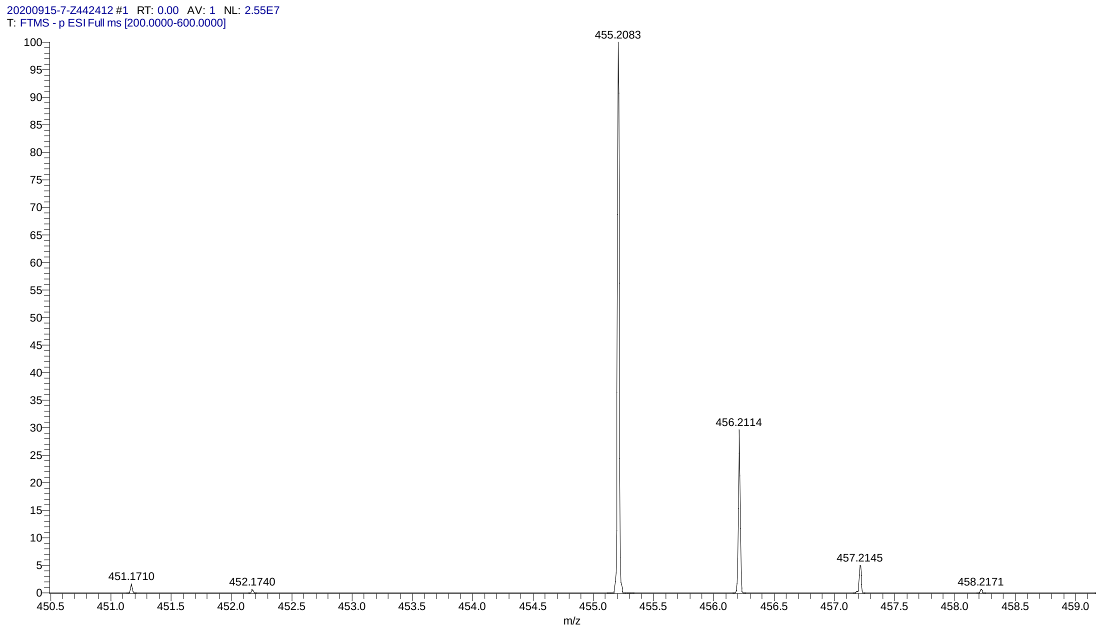

Figure S61. HR-ESI-MS spectrum of **7**
